# Photovoice Utilization for Research in Cancer Survivors: A Systematic Review

**DOI:** 10.1002/pon.70431

**Published:** 2026-03-18

**Authors:** Rebecca L. Hoover, Jingle Xu, Margaret Hammersla, Brian Garcia, Stephanie Betancur, Nathaniel Woodard

**Affiliations:** ^1^ Mark and Robyn Jones College of Nursing Montana State University Bozeman Montana USA; ^2^ University of North Carolina at Chapel Hill School of Nursing Chapel Hill North Carolina USA; ^3^ Yale University School of Nursing New Haven Connecticut USA; ^4^ University at Buffalo School of Public Health and Health Professions Buffalo New York USA

**Keywords:** cancer survivorship, participatory research, photovoice, visual methods

## Abstract

**Purpose:**

Photovoice integrates photography with narrative storytelling to examine lived experiences of participants. This review synthesizes the applications of photovoice in cancer survivorship research, focusing on study populations, emergent themes, and employed methods.

**Methods:**

We followed the Preferred Reporting Items for Systematic Reviews and Meta‐Analyses (PRISMA) guidelines for this review. We searched PubMed, CINAHL, and Scopus from database inception through April 12, 2025. Two reviewers independently screened 325 records in Covidence and extracted study characteristics, methods, key findings, and recommendations from the included studies. We used narrative synthesis to integrate the findings of the included studies. We appraised study quality using the Critical Appraisal Skills Program (CASP) Qualitative Checklist and excluded the studies rated weak.

**Results:**

Twenty‐six studies were included. Sample sizes ranged from 3 to 316; 20/26 studies enrolled 20 participants or fewer with male survivors were underrepresented (7/26 studies were female only). Findings were clustered into four domains: psychosocial and emotional experiences (26/26), systems of care and structural barriers (19/26), agency/expression/advocacy (23/26), and health behavior/lifestyle change (6/26). Studies rarely reported community dissemination, participant co‐analysis/member checking, or detailed ethical protocols.

**Conclusions:**

Photovoice is increasingly used to capture survivor perspectives, but the depth of participation and consistency of reporting vary. By centering survivor perspectives rarely captured in survivorship research, this study addresses a critical gap and generates insights to inform patient‐centered survivorship care. Future work should strengthen transparent methods reporting, ethical safeguards (including confidentiality and image ownership), inclusive recruitment strategies, and integration of photovoice into interventions and policy efforts.

## 1 Background

An increasing number of people are living with, through, and beyond cancer, underscoring the need to understand survivorship as a complex continuum that begins at diagnosis and extends across treatment and long‐term follow‐up. Globally, an estimated 53.5 million people were alive within five years of a cancer diagnosis in 2022 [1]. In the United States, there were an estimated 18.1 million cancer survivors in 2022, a figure projected to increase by more than 50% by 2040 [2]. This projected increase is due an aging and growing population, improvements in early detection, as well as advancements in treatment and survival [2]. Survivorship definitions have historically emphasized the post‐treatment period; however, contemporary frameworks describe survivorship as spanning diagnosis through short‐ and long‐term post‐treatment [2, 3]. For this review, a cancer survivor is defined broadly as an individual living with, through, or beyond cancer, regardless of treatment status.

Cancer survivors can experience persistent physical symptoms and late effects of therapy, psychological distress, changes in roles and identity, and economic and practical challenges such as employment, access to care, and financial toxicity [4, 5, 6, 7]. These experiences are shaped by demographic, clinical, and geographic contexts, contributing to inequities across cancer survivors. Understanding survivors' lived experiences is therefore essential for developing person‐centered care models and ensuring that survivorship programs and policies are inclusive of and responsive to diverse backgrounds and needs.

Developed by Wang and Burris (1997), photovoice is a participatory research method that combines participant‐generated photographs with written and/or spoken narratives to document and communicate lived experiences [8]. Grounded in community‐engaged research principles, photovoice can empower cancer survivors by enabling them to visualize and narrate their journeys [9, 10]. This process also facilitates emotional expression, connection, and resilience at both individual and community levels [9, 10].

Photovoice is well‐suited for engaging underrepresented individuals in research because it can be delivered in flexible formats (in person, online, or hybrid) and uses multimodal elicitation (i.e., combining visual images with written or spoken narratives), which can be adapted to survivors' needs and contexts [8]. For example, flexible delivery facilitates participation among groups dispersed over wide geographic areas, such as adolescent and young adult (AYA) survivors living in rural or frontier communities [11]. By centering perspectives and expertise of underrepresented survivors, photovoice advocates for inclusive, equity‐focused survivorship research [12].

The purpose of this systematic review is to synthesize the application of photovoice in current cancer survivorship studies. Specifically, we aim to (1) describe participating survivor populations and study procedures, (2) summarize key themes and outcomes, and (3) appraise methodological strengths, limitations, and directions for future research.

## 2 Methods

This review followed the Preferred Reporting Items for Systematic Reviews and Meta‐Analyses (PRISMA) [13] guidelines and was registered with PROSPERO (CRD42024616188). Researchers systematically reviewed studies to synthesize the use of photovoice among cancer survivors.

### 2.1 Search Strategy

The last search occurred in three databases, PubMed, CINAHL, and Scopus, on April 12, 2025. Search terms were developed in collaboration with a health sciences librarian and informed by a previous scoping review of photovoice. The strategy was iteratively refined through pilot searches and assessment of retrieved records to ensure comprehensive inclusion of qualitative and participatory methodologies. The finalized strategy is presented in Supporting Information S1: Appendix A1. To enhance completeness, the reference lists of all included studies and relevant reviews were manually screened; however, this did not yield any additional eligible studies. Using the defined search strategy (summarized in Supporting Information S1: Appendix A1), 325 titles and abstracts were independently screened by two reviewers, resulting in 57 full‐text articles reviewed for eligibility.

### 2.2 Study Selection

All search results were imported into Covidence for screening and duplicate removal [14]. Two independent reviewers assessed titles and abstracts based on predefined inclusion criteria. Studies were included if they involved participants with a cancer diagnosis, used photovoice as a primary research method, were published in a peer‐reviewed journal, and were written in or translated into English. Studies using photovoice in combination with other qualitative methods were also included. There were no restrictions on publication date, geographic location, or study duration. Studies were excluded if survivor‐specific results could not be distinguished from those of caregivers or healthcare professionals, were review articles or protocol papers, or had not undergone peer review (e.g., theses or dissertations). Both reviewers independently assessed the full‐text articles, and reasons for exclusion were documented. Full inclusion and exclusion criteria are summarized in Supporting Information S1: Appendix A2.

### 2.3 Data Extraction and Synthesis

Data were extracted from each study and entered into a standardized data table. Both reviewers independently completed the extraction process, and discrepancies were resolved via consensus. Extracted data included study details (e.g., first author, year, location), methodological characteristics, participant demographics, key findings (themes, subthemes, descriptions, and representative quotes), reported limitations, and study recommendations.

A narrative synthesis approach was used to integrate findings across the included studies, given heterogeneity in study aims, populations, and analytic approaches [15, 16]. This method is well‐suited for systematically summarizing qualitative and mixed‐methods evidence when meta‐analysis is not feasible due to variability in study designs and outcomes. The guidance by Popay et al. (2006) and Rodgers et al. (2009) for conducting and reporting narrative synthesis in systematic reviews was followed [15, 16]. Vote counting was used to synthesize study characteristics and methodologies. Two reviewers then applied thematic analysis to develop overarching thematic domains from the extracted key findings and identify recurring recommendations for future studies from the included studies.

### 2.4 Study Quality Assessment

The quality of the included studies was assessed using the Critical Appraisal Skills Program (CASP) Qualitative Checklist [17]. This checklist involved 10 items evaluating the validity (Item 1–6), rigor (Item 7–9), and relevance of studies (Item 10). Each study was independently appraised by two reviewers, and ratings were predefined based on the number of criteria met (e.g., strong = 8–10, moderate = 5–7, weak = 0–4). Conflicts were resolved through discussion between the two reviewers. Studies rated as “weak” were excluded from the final synthesis. Appraisal results are presented in Supporting Information S1: Appendix A3, with the checklist presented in Supporting Information S1: Appendix A4.

## 3 Results

**FIGURE 1:**
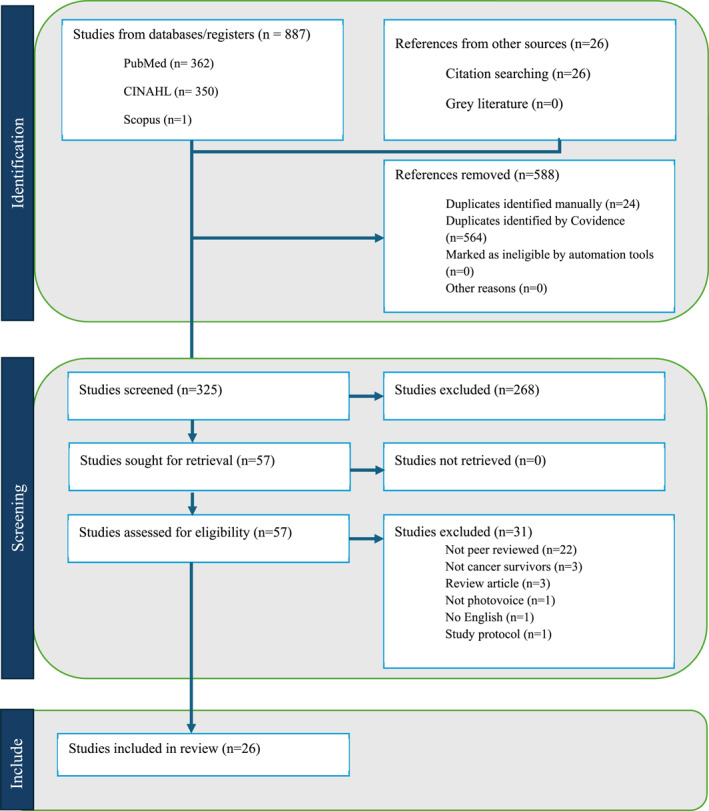
PRISMA diagram.

A total of 26 peer‐reviewed articles met the inclusion criteria and were included in the final analysis (see the PRISMA diagram in Figure 1). Excluded studies were non‐peer‐reviewed articles (*n* = 22), those that did not isolate outcomes of the cancer survivor population (*n* = 3), reviews (*n* = 3), not in English (*n* = 1), protocols (*n* = 1), and studies that did not report outcomes found via the photovoice method (*n* = 1). All 26 studies were rated as “moderate” or “strong” according to CASP appraisal criteria and were retained for synthesis.

Of the 26 included reports, 20 reported unique studies. In contrast, two research teams produced multiple publications from the same dataset, each addressing distinct outcomes or subpopulations. For example, Edwards et al. generated three articles on different research questions [18, 19, 20], two of which were derived from a single dataset. Similarly, Morrison et al. published three articles analyzing the same dataset to address different research questions related to returning to work among males and females [21, 22, 23]. Key study characteristics and findings are summarized in Table 1, with recommendations presented in Table 2. Full reporting of study details, including recruitment and eligibility criteria, study type, location, aims or research questions, and analytic methods, is provided in Supporting Information S1: Appendix A5. Participant demographics, primary results (themes and illustrative quotes), limitations, and recommendations are detailed in Supporting Information S1: Appendix A6.

**TABLE 1:** Key characteristics of reviewed studies employing photovoice in cancer research.

First author, year, study location	Participant sample demographics	Study focus	Photovoice methods	Limitations	Recommendations
Bates, M. J. 2018 [24] Blantyre, Malawi	*N* = 13 Age range: Not reported Gender: 50/50 mix of males and females *N* = 7 cancer survivors, *n* = 6 caregivers Setting: Outpatient Cancer diagnosis: Mixed diagnosis (Kaposi's sarcoma, brain, cervical, thyroid) Cancer treatment stage: Post treatment (mean = 42 months)	To explore how patients with advanced cancer and their caregivers define wellbeing and the role of palliative care in supporting quality of life.	One month study using digital camera to document “the story of my illness.” images were then selected in one‐on‐one sessions before the group sessions. Researchers took field notes during all sessions.	Finding specific to urban/peri‐urban Malawi. Long‐term relationships between palliative care staff and co‐researchers facilitated accountability and acceptance of the study, while also introducing some bias.	Train healthcare workers on culturally competent care for patients facing discrimination. Develop financial aid programs for low‐income families caring for cancer patients. Enhance support groups for caregivers and patients to reduce social isolation.
Bood, Z. M. 2022 [25] Netherlands	*N* = 12 Age range: 23–35 years old (mean = 31) Gender: 11 females, 1 male 100% cancer survivors Setting: Outpatient Cancer diagnosis: Mixed diagnosis (lymphoma, breast, ovarian, brain, testicular) Cancer treatment stage: Mixed treatment phases: *n* = 6 receiving curative treatment, *n* = 5 receiving palliative treatment, *n* = 1 did not specify	To explore how adolescents and young adult cancer patients and survivors experience life with cancer using visual storytelling techniques.	Two days of work with a photographer, the participants were given six domains to address, these photos were then turned into a book.	Primarily female participants. Participants self‐selected and had an interest in visual, creative expression	Integrate visual tools in AYA cancer care to help patients articulate experiences beyond verbal descriptions.
Capewell, C. 2020 [26] United Kingdom	*N* = 20 Age range: Not reported Gender: 100% female 100% cancer survivors Setting: Outpatient Cancer diagnosis: 100% breast cancer Cancer treatment stage: Not reported	To gain insight into how women perceive their experiences of diagnosis and treatment of breast cancer to better inform health care professionals.	One‐month total study time: participants meet with researchers for introduction to the study as well as a trial on photovoice using specific prompts. Then 2 weeks later the participants and researchers met again, with the session being audio recorded, the participant shared images and captions, allowing for theme development from a participant led discussion.	All female participants. No direct involvement of participants in data analysis.	Information gathered from similar studies can be used to improve patients experience with wait times, and education, and reduce anxiety around scheduling and waiting. Allows participants a place to express themselves and give insight to health care professionals who are taking care of the survivors.
Currin‐McCulloch, J. 2024 [27] Online in United States	*N* = 11 Age range: 28–44 (mean = 31) 100% female 100% cancer survivors Setting: Outpatient Cancer diagnosis: Not reported Cancer treatment stage: On active treatment or within 5 years of diagnosis	To evaluate the feasibility, acceptability, and benefit of an 8‐week synchronous meaning‐making photovoice group work intervention among a young adult cancer survivor sample.	Eight‐week intervention, with virtual weekly group sessions lasting 1.5 h. Participants received prompts prior to each group session, at the group sessions participants were encouraged to share their images and captions. There was an options participation in a photo exhibit at the end of the intervention	All female participants. Challenges with recruitment and engagement as the study took place during the 2020–2021 pandemic.	Virtual photovoice study allowed for more flexibility in sharing, and discussion sessions. Future studies should increase outreach to ensure heterogeneity in gender, race, and other demographics.
Ebrahimpour, F. 2021 [28] Tehran, Iran	*N* = 20 Age range: 6–12 years old Gender: 50/50 males and females 100% cancer survivors Setting: Inpatient Cancer diagnosis: Acute lymphoblastic leukemia, ewings sarcoma, lymphoma, and abdominal malignancy Cancer treatment stage: On active treatment	To explore how hospitalized children with cancer perceive hope and identify factors that foster hope in an oncology ward.	Single day study. Orientation as to how to use camera, then 15 min of photo taken around the hospital floor. Followed by 15–30 min of individual interviews to understand why those were the chosen photos.	Due to the age of the participants, a lengthy interview and/or discussion session was not feasible.	This was a creative intervention that children might already be doing (using a photo to document), this allowed for some space to process their experience of living with a cancer diagnosis.
Edwards, L. B. 2017a [19] Edwards, L. B. 2017b [20] Edwards, L. B. 2017 [29] South Africa	*N* = 316 Age: 8 months to 86 years old for cancer survivors, younger cancer survivors caregivers were used as proxy Gender: 138 males, 148 females *N* = 286 cancer survivors and family, *n* = 30 oncology workers Setting: Inpatient Cancer diagnosis: Breast cancer, leukemia, prostate cancer, cervical cancer, head and neck cancers, kidney cancer Cancer treatment stage: Not reported	To identify and describe grassroots cancer‐related challenges and thereby contribute to what is known about the experience of coping with cancer in context of South Africa.	Face to face interview with photo prompts given, no timeline reported, not repeated, no participant involvement in the analyses.	Under‐representation of end‐of‐life patients.	Training, mentorship, and emotional support systems for oncology workers. Promote patient empowerment and rights in the south african health‐care context. Additional research for “end of life” phase of cancer to increase public knowledge.
Georgievski, G. 2018 [30] Toronto, Ontario, Canada	*N* = 6 Age range: 13–18 years old Gender: 4 females, 2 males 100% cancer patients Setting: Inpatient Cancer diagnosis: Osteosarcoma, anaplastic large cell lymphoma, hodgkins lymphoma, acute myeloid leukemia, rhabdomyosarcoma Cancer treatment stage: On active treatment	To explore the psychosocial needs of adolescents undergoing active cancer treatment using photovoice.	7‐week, of weekly 90‐min sessions. Schedule of plan for weekly sessions. During each session participants shared photos/narratives, discussed the relation to that week's theme, and learned of the theme for the following week. Final project was a selection of the most impactful and representative photographs for a gallery exhibition.	Sample from a single hospital. Potential self‐selection bias.	Presented an opportunity to meet other teens with cancer and establish important relationships that extended beyond group. Make sure the setting fosters safety to allow for open and honest discussions of difficult and sensitive topics. Allows for teens with cancer to share their insight that can impact important practice and policy changes.
Hammond, C. 2016 [31] British Columbia, Ontario, and Quebec, Canada	*N* = 58 Age range: 27–81 years old, mean = 61 years Gender: 100% female *N* = 27 cancer survivors, *n* = 31 caregivers Setting: Outpatient Cancer diagnosis: Breast, colon, kidney, skin, non‐Hodgkin's, and lymphoma Cancer treatment stage: In survivorship	To develop and disseminate knowledge of first nations women's experiences with cancer survivorship, either as a cancer survivor or caregiver.	4‐week study, with 3 phases: 1‐Introduction/instructions (3 weeks to complete the art‐based activity), 2‐follow up individual interview, 3‐follow up group interview.	Limited to first nations communities. All female participants, limiting perspectives.	Developing culturally safe cancer care programs. Train healthcare professionals on historical trauma and first nations perspectives on cancer. Expansion of cancer support groups in first nation communities. Encourage open discussions about cancer through community‐led awareness programs.
Jellema, P. 2018 [32] Leuven, Belgium	*N* = 3 Age range: 37–67 years old Gender: 2 females, 1 male 100% cancer survivors Setting: Outpatient Cancer diagnosis: Mixed cancer types Cancer treatment stage: Mixed, on/off treatment	To identify common threads in how cancer care environment is experienced by cancer patients and spatial aspects that play a role in that experience	One‐on‐one interview in the participants home.	Lack of diverse sample. Reliance on participant willingness to take photos.	Facilitate peer interaction opportunities to enhance informal support in shared treatment areas. Ensuring that home‐based care feels secure, providing remote access to specialists.
Jellema, P. 2020 [33] Leuven, Belgium	*N* = 15 Age range: 28–71 years old Gender: 2 males, 3 females *N* = 5 cancer survivors, *n* = 5 caregivers, *n* = 5 care professionals in oncology departments Setting: Inpatient Cancer diagnosis: Not reported Cancer treatment stage: Not reported	To provide insights into the roles cancer care facilities play in the well‐being of people affected by cancer.	1‐ 30–90‐min interview with triad of survivor/caregiver/worker. 2‐ Two follow up interviews with the survivor.	Possible selection bias as participants were recruited from personal and professional networks. Heterogeneity of cancer diagnosis and situations of survivors make comparisons difficult. Survivors had to be healthy enough to participate in study, therefore no “very ill” survivor perspectives were included.	Create homelike spaces in hospitals to enhance emotional comfort. Increase flexibility and comfort for spaces within care facilities that significantly impact patient experiences.
Lopez, E. D. S., 2005 [34] Eastern North Carolina, United States	*N* = 13 Age range: 44–82 years old Gender: 100% female *N* = 12 cancer survivors, *n* = 1 mother who had recently lost her daughter to breast cancer Setting: Outpatient Cancer diagnosis: 100% breast cancer Cancer treatment stage: Not in active treatment	To record (in their own words) how the quality of life for 13 african american breast cancer survivors is affect by their social context. To develop a quality of life framework to inform interventions to address the social forces that drive long‐term concerns of rural african americans survivors.	Two training sessions followed by monthly 3 h meetings to discuss the photos/narratives from the previous months. Five assignments in total, took place over 7 months (participants choose to talk about 2 topics for 2 sessions).	Limited to females and breast cancer diagnosis. Lack of trust by survivors for healthcare providers and support programs.	Expansion of culturally tailored cancer support groups for african american women. Acknowledge racial concerns by survivors. Develop church/faith‐based cancer education programs.
Morrison, T. L. 2015 [22] Morrison, T. L. 2014a [23] Morrison, T. L 2014b [35] Ottawa, Ontario, Canada “Survivors experience return to work”	*N* = 20 Age range: 30–89 years old Gender: 10 females, 10 males 100% cancer survivors Setting: Outpatient Cancer diagnosis: Mixed cancer types; one study reported female‐only diagnosis: Breast, colorectal, pancreatic, hematologic Cancer treatment stage: Survivorship/not on active treatment	To elucidate the lived experiences of cancer survivors related to both their work return and maintenance. Studies compared the experiences of women only, men to women, and described the variable approaches used by participants to conceal or disclose.	Two interviews: 1 for explanation/introduction and 1 month later survivors produced 1–12 photographs at the interview that hold some personal meaning of work return/maintenance.	Participants were mostly professionals with high income. Not representative of survivors who were not able to return to work, had physically demanding work, or for whom return to work was necessary for survival.	Introduction of formal return to work policies that include flexible work hours. Expand access to mental health and peer support programs in workplaces.
Mosavel, M. 2010 [36] Cleveland, Ohio, United States	*N* = 17 Age range: 23–60 years old (mean = 45) Gender: 11 females, 6 males *N* = 7 cancer survivors, *n* = 10 caregivers Setting: Not reported Cancer diagnosis: Not reported Cancer treatment stage: Not reported	To understand the barriers and challenges that african american cancer survivors experience after being diagnosed with cancer.	Four sessions in two groups: First 2 session were educational; second 2 sessions were to discussion the photos taken. Each session lasted 3 h.	Primarily female participants. No reporting of specific cancer type or point along the cancer continuums.	Increase financial aid programs for african american cancer survivors. Develop culturally relevant survivorship support groups in urban black communities. Improve access to affordable wigs, makeup, and appearance‐related resources. Provide better education on post‐treatment care (e.g. nutrition, exercise, and mental health).
O'Callaghan, M. 2024 [37] Ireland	*N* = 8 Age range: 45–59 year old (mean = 51) Gender: 7 females, 1 male 100% cancer survivors Setting: Outpatient Cancer diagnosis: Breast, prostate Cancer treatment stage: At least 6 months post treatment	To use a visual methodology called photovoice to understand the significance of nutrition for Irish cancer survivors.	8 weeks of research. Three sessions: Introduction: 1 h workshop of the project, taking photos, ethics, visual literacy. (2 weeks to take photos) Individual interviews: 30–60 min to share photographs. (2 weeks to create captions) Group workshop: Introductions, shared photos/captions.	Primarily breast cancer, and female. Participants had higher levels of education than the general survivor population.	Encourage self‐directed nutrition education while promoting scientific accuracy. Acknowledge the emotional and social aspects of food in survivorship care.
Pailler, M. E. 2020 [38] Buffalo, New York, United States	*N* = 30 Age range: 17–36 years old (mean = 28) Gender: 23 female, 7 males 100% cancer survivor Setting: Outpatient Cancer diagnosis: Leukemia/lymphoma, thyroid, cervical, melanoma, and brain Cancer treatment stage: Not reported	To assess the feasibility and preliminary efficacy of the photographs of meaning program (POM‐AYA) for adolescent and young adult cancer patients and survivors.	10 weeks intervention via mobile application PixStori. Pre‐training about using PixStori app, instructions to take at least 2 photo and post them each week related to the weekly theme with audio or text narration. Participants have ability to view, like, and comment on others posts.	Primarily female participants	
Park, J. S. 2020 [39] Busan, South Korea	*N* = 9 Age range: 53–65 years old Gender: 100% female 100% cancer survivors Setting: Outpatient Cancer diagnosis: 100% breast cancer Cancer treatment stage: Post treatment	To develop a weight management strategy for breast cancer patient through in‐depth analyses of their experiences of weight management using photovoice after receiving tamoxifen in korea.	Two week study timeline: Education on protocol/methods and introduction of prompts (4 in total). At the end of 2 weeks the participants submitted and selected three photos for each prompt and shared an explanation.	Sole focus on survivors treated with tamoxifen, excluding those on other hormone therapies. Did not account for long‐term outcomes of weight management interventions.	Long‐term weight management support programs. Enhancing access to community fitness and nutrition programs tailored for survivors. Provide psychological support for stress‐induced eating behaviors. Encourage family involvement in weight management efforts.
Poudrier, J. 2009 [40] Saskatchewan, Canada	*N* = 12 Age: 42–75 years old Gender: 100% women 100% cancer survivors Setting: Outpatient Cancer diagnosis: 100% breast cancer Cancer treatment stage: At least 6 months post treatment	To explore and being to make visible aboriginal women's experiences with breast cancer using the qualitative research technique, photovoice.	6‐week study with three meetings: 1‐Education/information session on study purpose and photovoice methods. Participants borrowed digital cameras for several weeks to document what having breast cancer meant to them. 2‐Interview where women shared their photos, provided a description of what the photo was meant to convey, and choose several photos they felt were especially meaningful. 3‐A single day even with a sharing circle in the morning with just participants, and in the afternoon stakeholders from advocacy groups joined.	Only female and only breast cancer diagnosis were included. Limited to the aboriginal communities in Saskatchewan, Canada.	Train healthcare professionals in cultural competency to reduce racism and improve patient communication. Develop aboriginal‐specific breast cancer support groups to create safe spaces for survivors. Implement patient navigation programs led by indigenous healthcare workers.
Power, R. 2022 [41] Australia and other English‐speaking countries (United States, United Kingdom, New Zealand, and Canada)	*N* = 55 Age range: 15–92 Gender: 52% female, 33% male, and 15% different gender identity *N* = 45 cancer survivors, *N* = 10 caregivers Setting: Outpatient Cancer diagnosis: Mixed diagnosis Cancer treatment stage: Not reported	To address the gap in the research literature by examining subjective experiences of minority stress and social support among LGBTQI people with care and their carers, drawing on qualitative findings from the mixed method out with cancer study (CITE).	Participants were invited to submit 3–5 photographs that represented their experiences with cancer, which were then discussed in an individual interview.	Only English speaking countries were included during recruitment. Participants had been part of a larger study (out with cancer), and might have self‐selected to be included in the photovoice study.	Professional education and training to create environments in which LGBTQI patients and carers access care and support during cancer need to be culturally safe and inclusive. Inclusion of carers (partners and chosen family) in consultations and support is essential. Investment in peer‐led initiatives that provide connection and support to LGBTQI community. Reduce the impact of minority stress on well‐being and on interactions with in the healthcare system for the LGBTQI cancer population.
Wong, S. S. 2019 [42] Winston‐Salem, North Carolina, United States	*N* = 20 Age range: 40–79 years old Gender: 11 females, 9 males *N* = 13 cancer survivors, *n* = 7 caregivers Setting: Outpatient Cancer diagnosis: 100% primary pancreatic ductal adenocarcinoma Cancer treatment stage: Post treatment	To gain a richer understanding of the factors associated with psychological distress from patient and caregiver perspectives.	Two biweekly 90 min session in small group format (2–3 participants). First to educate/inform on study purpose and methods. Then participants took photos and brought them to small groups to share. At each session the participants came up with the prompt for the next session.	Sample may have better prognosis then general pancreatic cancer population. Lack of racial/ethnic diversity in participants.	Improve how prognosis is communicated to reduce emotional distress. Develop nutritional‐focused interventions to help patients manage weight loss and gastro‐intestinal symptoms. Provide caregiver support programs to prevent burn‐out and role strain. Create long‐term survivorship plans for patients with resectable tumors to address fear of recurrence. Encourage peer‐support programs for patients and caregivers to share coping strategies.
Wong, C. L. 2024 [12] Hong Kong, China	*N* = 34 Age range: Survivors 8–17 years old (mean = 14.5 years) Gender: Survivors 10 female, 7 male *N* = 17 survivors, *n* = 17 parents (mostly mothers) Setting: Outpatient Cancer diagnosis: Leukemia, neuroblastoma, rhabdomyosarcoma, wilms tumor, osteosarcoma, medulloblastoma, astrocytoma Cancer treatment stage: Not reported	To understand the experiences of cancer survivorship among pediatric and adolescent cancer survivors and their parents by employing a photovoice approach.	4‐week study, participants were instructed to take 10 photographs over the 4 weeks and write a narrative for each photo. Individual interviews were completed with each pair (survivor and parent).	Specific to Hong Kong. No further comparative analysis was conducted to explore any thematic differences based on age. Survivorship challenges and priorities could evolve and change overtime with perspective varying based on age.	Develop structured survivorship programs to address late effects and emotional well‐being. Promote peer mentorship programs to help new survivors navigate post‐treatment life. Integrate faith‐based and cultural support systems into survivorship care.
Yi, J. 2010 [10] Los Angeles, California, United States	*N* = 12 Age range: Survivors 18–39 years old Gender: Survivors 4 female, 2 male *N* = 6 survivors, *N* = 6 caregivers (5 parents, 1 spouse) Setting: Outpatient Cancer diagnosis: Acute lymphoblastic leukemia, osteosarcoma, brain tumor Cancer treatment stage: 5 years post treatment	To report on themes that young adult cancer survivors and family members (caregivers) identified as relevant to their experience of cancer survivorship.	A total of seven sessions were held every 20 weeks on Saturday mornings at Children's hospital Los Angeles. The first meeting consisted of an orientation to the project. Staff distributed digital cameras, instructed them in their use, and then directed them to take a self‐portrait. At the end of day 1, participants are asked to take photos over the next 2 weeks to capture “the impact of cancer on your family.” all subsequent topics for future photographs were generated by the participants themselves through group discussions during each of the subsequent sessions. Each of the next five sessions lasted 2 h. After the individual interviews, participants were divided into a YACS group and a family member group and participated in an hour‐long group discussion. At the end of the hour, each group reached consensus on a theme for the next photo‐documentation period.	Sample were relatively healthy volunteers and actively involved in the local community‐based agency's (PADRES) activities.	Develop structured programs for young adult cancer survivors to support career development and social reintegration. Provide caregiver‐focused interventions addressing stress and emotional exhaustion.
Yi, J. 2016 [43] Seoul, South Korea	*N* = 7 Age range: 20–27 years old (mean = 24 years old) Gender: 4 females, 3 males 100% cancer survivors Setting: Outpatient Cancer diagnosis: Acute lymphoblastic leukemia, Malignant lymphoma, Osteosarcoma, Neuroblastoma, Brain tumor, and ovarian cancer Cancer treatment stage: Completed cancer treatment	To investigate the impact of childhood cancer experiences on young adult long‐term survivors in korea.	5‐week study period with weekly session for 3 h at a time. 1‐ orientation and then a group discussion on cancer survivorship. At the end of the initial and four subsequent sessions, the group decided on the topic that they would take photos about in the coming week before the next session. 2‐ subsequent sessions, each participant was asked to select two or three key photos, which were projected onscreen in a group discussion (1.5–2 h long).	Participants were relatively well adjusted to society, not representative of all survivors. Focus on long‐term survivors, no information on those still on active treatment or in surveillance.	Education programs to combat cancer stigma in schools and workplaces. Support groups for young adult survivors to develop social skills. Career counseling and workplace advocacy to ensure fair treatment. Mental health services to address survivor self‐esteem and relationship concerns.

**TABLE 2:** Summary of recommendations from included photovoice studies (*n* = 26).

Domain	Key recommendations	Representative studies
Equity and access	Tailor photovoice to underserved populations; compensate participants; reduce participation barriers	Lopez (2005), Mosavel (2010), Bates (2018), Hammond (2016) [24, 31, 34, 36]
Methodological rigor	Use iterative coding, triangulation, co‐design; improve clarity in reporting	Jellema (2020), Currin McCulloch (2024), Morrison (2015) [21, 27, 33]
Participant voice and storytelling	Center survivor narratives in all research phases; incorporate visual data into analysis and dissemination	Bood (2022), Georgievski (2018), Wong (2019), O'Callaghan (2024) [25, 30, 37, 42]
Ethical standards	Standardize protocols for consent, confidentiality, and emotional safety	Morrison (2015), Pailler (2020) [23, 38]
Knowledge translation	Use exhibitions, community forums, and media to inform policy and practice; engage stakeholders early	Capewell (2020), Lopez (2005), Edwards (2017), Poudrier (2009) [20, 26, 34, 40]
Clinical integration	Embed photovoice in support groups, rural outreach, and psychosocial care; train providers in visual storytelling	Ebrahimpour (2021), Edwards (2018), Currin McCulloch (2024) [18, 27, 28]

### 3.1 Study Characteristics

The studies encompassed a diverse range of geographic settings; yet a disproportionate focus remains on high‐income countries. According to the 2024–2025 World Bank classifications [35], the majority of studies (*n* = 20) were conducted in high‐income countries, including: Canada (*n* = 6), the United States (*n* = 6), South Korea (*n* = 2), Belgium (*n* = 2), the United Kingdom (*n* = 1), Ireland (*n* = 1), the Netherlands (*n* = 1), and a multi‐country English‐speaking sample from high‐income countries (*n* = 1). Four studies were conducted in upper‐middle‐income countries: South Africa (*n* = 3) and China (*n* = 1). Two studies were conducted in lower‐middle‐income countries: Iran (*n* = 1) and Malawi (*n* = 1). No studies were identified from low‐income countries. Most studies employed only qualitative approaches (*n* = 25) while one was a mixed‐methods study. The majority of studies were conducted in outpatient (*n* = 19), while the remainder were inpatient (*n* = 6), or not reported (*n* = 1) settings. A larger number of studies were conducted in urban locations (*n* = 14) than in mixed urban and rural settings (*n* = 8), rural‐only settings (*n* = 2), or settings not specified (*n* = 2).

### 3.2 Participant Demographics

Sample sizes ranged from 3 to 316 participants, with 20 of the 26 studies enrolling 20 or fewer participants. Gender was reported in 25 of the 26 studies; seven studies included only female participants. Participant ages, reported in 24 studies, ranged from 8 to 92 years. Four studies focused solely on breast cancer; one study focused only on a type of pancreatic cancer. In contrast, 19 studies included various types of cancer, and three did not specify the type of cancer participants were diagnosed with.

The majority of the included studies did not restrict participation based on race or ethnicity (*n* = 22); two studies focused on African Americans with cancer, and two other studies focused on Aboriginal and First Nations cancer survivors. Most studies involved cancer survivors only (*n* = 14), followed by survivors and caregivers (*n* = 8), and survivors, caregivers, and healthcare providers (*n* = 4). Sixteen studies reported participants' cancer treatment status (post‐treatment *n* = 11, active treatment *n* = 2, mixed treatment stages *n* = 2).

### 3.3 Study Methodologies and Reporting

All studies employed photovoice as a core methodology, often supplemented by semi‐structured interviews (*n* = 11) and group discussions (*n* = 15). Thematic analysis was the most frequently used analytic method (*n* = 25), followed by iterative coding (*n* = 5), comparative analysis (*n* = 5), and collaborative interpretation (*n* = 5). Some studies enhanced methodological rigor through community validation (*n* = 3), triangulation (*n* = 3), or expert panel review (*n* = 1). Reporting formats included key themes (*n* = 12), visual data (*n* = 12), narrative descriptions (*n* = 10), participant quotes (*n* = 9), and combined qualitative and quantitative findings (*n* = 6).

### 3.4 Narrative Synthesis

Four overarching thematic domains illustrate how photovoice has been used to explore the cancer survivorship experience: (1) psychosocial and emotional experiences, (2) systems of care and structural barriers, (3) agency, expression, and advocacy, and (4) health behavior and lifestyle change. Table 3 summarizes subdomains, the number of studies, and a representative quote for each domain. The complete list of quotes is available in Supporting Information S1: Appendix A6. The four thematic domains reflect two complementary analytic aims: the first two themes synthesize common survivorship experiences reported across studies, while the latter two capture how Photovoice was operationalized as a method to facilitate agency, expression, and behavior change.

**TABLE 3:** Thematic summary of photovoice cancer research.

Category	Quotes
Theme 1: Psychosocial and emotional experiences (*n* = 26) Captures internal and interpersonal processes shaping emotional and social navigation of cancer; reflects existential, emotional, and relational impact.	
Identity disruption and redefinition (*n* = 26)	“You can't see anything from the outside. That's why they often cannot believe it, cannot comprehend it. […] sometimes I fantasize about being bald. Would people then realize that is this real?” Bood, ZM, 2022 [25]
Emotional and psychological distress (*n* = 20)	Upon learning of cancer diagnosis, “Am I going to die?” Capewell, C, 2020 [26]
Social support and isolation (*n* = 22)	“The people who happen to encourage us on some other things for example maybe at home….being free with you by talking to you and you are not supposed to be worried and this is not the end but the beginning….those are the category of people assisting you in your everyday like the everyday food and activities.” Bates, MJ, 2018 [24]
Spirituality and meaning‐making (*n* = 15)	“This is the praying room, here mothers pray for our health. It gives me a sense of hope that we will get better.” Ebrahimpour, F, 2021 [28]
Theme 2: Systems of care and structural barriers (*n* = 19) Includes experiences with healthcare access, navigation, and equity; highlights how external systems shape the cancer journey.	
Health care experiences and system barriers (*n* = 17)	“The clinic kept thinking it was worms or HIV and only have 3 months sent him to hospital where he was misdiagnosed with constipation, and eventually referred to the cancer hospital.” (PvM283, mother of a 4 year old son; acute lymphoblastic leukemia) Edward, L.B., 2017 (child study) [20]
Environment and place (*n* = 19)	“All the YACS had dreadful memories of cancer treatment. For example, *Roberto described his hospital days as ‘being in jail.*’ on the other hand, most of the survivors shared the stories of breaking hospital rules and going outside, and they remembered these events as ‘having fun’ and ‘feeling human again.’” Yi, J., 2010 [10]
Theme 3: Agency, expression, and advocacy (*n* = 23) Relates to empowerment, future orientation, and advocacy; illustrates reclaiming control and engaging through photovoice.	
Empowerment through expression (*n* = 3)	“Sharing my pictures and stories help me realize I wasn't alone.” Pailler, M.E, 2020 [38] “Because no matter what I can accomplish a difficult task… you know I have people who care about me and understand me. I say, ‘*Let it rain. I can handle it*.’” Yi, J. 2016 [43]
Return to normalcy and future orientation (*n* = 23)	“You know that look good, feel better thing?, you have to ascribe to it a little bit in that when you go in and people say, ‘*I think you look amazing’* and that kind of stuff and you ‘*You're looking great’* it makes you feel better. You release endorphins and then you just keep going.” Morrison, T.L., 2014 [22]
Theme 4: Health behavior and lifestyle change (*n* = 6) Reflects how daily practices like nutrition and exercise support healing and survivorship.	
Nutrition and lifestyle change (*n* = 6)	“I went back to doing what our parents ate, everything fresh and out of the ground ‐no added preservatives. We eat as natural as we can and the fewer processes that food goes through, the better it is for you.” O'Callaghan, N., 2024 [37]

#### 3.4.1 Psychosocial and Emotional Experiences (*n* = 26)

This domain encompasses four interrelated subthemes: identity disruption and redefinition (*n* = 26), emotional and psychological distress (*n* = 20), social support and isolation (*n* = 22), and spirituality and meaning‐making (*n* = 15). Across the literature, survivors described cancer as a rupture that reshaped not only how they viewed themselves, but also how they related to others and understood their lives moving forward. Through photography and narrative, survivors explored shifts in identity alongside experiences of grief, fear, and resilience, often grappling with the dissonance between their pre‐ and post‐cancer selves. These emotional processes were deeply embedded in social contexts: some images highlighted sources of connection and support, while others revealed isolation, loss of peer relationships, or a sense of being misunderstood by family and providers. Spirituality and meaning‐making emerged as mechanisms through which survivors sought coherence, whether through faith, reflection on mortality, or redefining purpose after treatment. Together, these subthemes illustrate that psychosocial adjustment in survivorship is not a singular process but a layered, relational experience that unfolds across emotional, social, and existential dimensions.

#### 3.4.2 Systems of Care and Structural Barriers (*n* = 19)

This domain involved subthemes of health care experiences and system barriers (*n* = 17) and environment and place (*n* = 19). Survivors described challenges navigating healthcare systems, including delays and misdiagnoses that often‐increased distress. For example, one parent recounted: *“The clinic kept thinking it was worms or HIV and only after 3 months sent him to hospital where he was misdiagnosed with constipation, and eventually referred to the cancer hospital”* [19]. Such challenges highlight the system‐level gaps in timely diagnosis and referral. Survivors also reflected on challenges relevant to hospital environments. One survivor described treatment as feeling “like being in jail,” and considered moments of “breaking hospital rules and going outside” as profoundly humanizing [10]. Together, these examples underscore how institutional practices, physical environments and policy together shape survivorship experiences, highlighting photovoice's unique capacity to capture these systemic barriers.

#### 3.4.3 Agency, Expression, and Advocacy (*n* = 23)

Empowerment through expression (*n* = 3) and return to normalcy and future orientation (*n* = 23) emerged as two related subthemes within this domain. Across studies, photovoice functioned as a mechanism through which survivors reframed their experiences and repositioned themselves as active agents rather than passive recipients of care. By selecting and interpreting their own images, survivors described gaining confidence in their ability to tell their stories and influence how their experiences were understood. One participant reported, “Because no matter what I can accomplish a difficult task… You know I have people who care about me and understand me. I say, ‘*Let it rain. I can handle it*.’” [43].

Photovoice also supported survivors in re‐envisioning life beyond treatment, often marking a psychological shift toward normalcy, goal‐setting, and hope for the future. Participants described how engaging in visual reflection helped them recognize personal growth and momentum—“*You know that look good, feel better thing? … It makes you feel better, you release endorphins and then you just keep going.”* [21] For some, this renewed sense of agency extended beyond individual reflection to advocacy, including raising awareness within their communities and contributing to research aimed at improving cancer care delivery [44]. For example, one study described survivors using Photovoice outputs to engage community stakeholders and raise awareness of gaps in survivorship services, illustrating the method's potential to support advocacy even when formal policy change was not evaluated. Together, these findings suggest that photovoice can support empowerment not only by validating survivor experiences, but by enabling forward‐looking meaning and action.

### 3.5 Health Behavior and Lifestyle Change (*n* = 6)

Six studies explored how photovoice promoted healthy behaviors and lifestyle changes during survivorship. Participants described improving diet and wellness practices, often tying these shifts to a renewed identity or motivation from cancer experiences. One survivor explained: *“I went back to doing what our parents ate, everything fresh and out of the ground—no added preservatives. We eat as natural as we can, and the fewer processes that food goes through, the better it is for you.”* [37] Photovoice not only helped document lifestyle changes but also helped survivors link these practices to healing and survivorship, reinforcing motivational mental power to sustain health behaviors after cancer treatment. However, there remains limited evidence on sustaining outcomes, underscoring the need for longitudinal evaluation of photovoice's impact on behavior changes.

### 3.6 Considerations When Using Photovoice in Cancer Survivorship Research

These studies suggest several key considerations for the use of photovoice in cancer survivorship research. One recurring theme was the need to enhance equity in research participation. Several studies have identified photovoice as an effective strategy for developing culturally relevant, survivor‐informed interventions, particularly for underrepresented populations [9, 24, 31, 36]. To enhance participation, researchers recommended minimizing logistical barriers (e.g., transportation, literacy, caregiving burdens), which was a strength of photovoice, and providing adequate compensation [9, 24, 31, 36].

Another consideration is to enhance methodological rigor and transparent reporting of photovoice procedures in future studies [21, 27, 33]. This includes clearly describing each step in the research process, from how participants were recruited to how photographs and narratives were collected, the iterative process of coding, and how triangulation and community validation or member checking occurred. This level of transparency strengthens the analytic credibility and allows for replicability of the research. Furthermore, survivors should be actively engaged throughout the entire research process, including defining research questions, shaping visual and narrative outputs, participating in data interpretation, and co‐creating dissemination strategies [21, 27, 33]. This orientation aligns with the participatory roots of photovoice, and is critical for understanding cancer survivors' complex life experiences.

Ethical considerations were also highlighted [21, 38]. Photovoice raises risks of unintended identity disclosure or emotionally triggering content, indicating the need for standardized ethical protocols tailored to this method. Creative dissemination avenues, such as photo exhibits, community dialogs, and policy briefs, can honor participants' voices while promoting system‐level impact and potential change [9, 19, 26, 40].

Finally, some studies proposed integrating photovoice with clinical survivorship practice [18, 27, 28]. This included using visual storytelling in support groups, survivorship care planning, rural outreach, and training oncology professionals in photo‐elicitation and narrative interpretation techniques. These applications position photovoice as more than a research method; they highlight its potential to advance the quality of care, health equity, and survivor‐centered systemic transformation [45, 46, 47, 48].

## 4 Discussion

This systematic review synthesizes the use of photovoice methods in cancer survivorship research, highlighting the significance of this method in capturing the stories told directly by survivors across the cancer continuum. We identified four thematic domains summarizing the key findings of the included studies: psychosocial and emotional experiences; systems of care and structural barriers; agency, expression, and advocacy; and health behavior and lifestyle change. Together, these themes highlight the strengths and limitations of photovoice in revealing survivors' real life after a cancer diagnosis and into survivorship [11, 44, 45].

### 4.1 Interpretation of Thematic Domains

#### 4.1.1 Psychosocial and Emotional Experiences

Findings across studies indicate that photovoice facilitates deeper reflection on the psychosocial and emotional dimensions of survivorship by allowing survivors to externalize and interpret experiences of identity change and psychological adjustment. Rather than merely documenting distress or resilience, photovoice supports meaning‐making through survivor‐led interpretation, positioning participants as active sense‐makers of their survivorship trajectories. This aligns with prior evidence demonstrating photovoice's utility in supporting emotional expression and coping, particularly among youth and individuals living with chronic illness [49, 50, 51, 52]. In this review, these processes appear to extend beyond individual reflection, suggesting photovoice's potential to generate emotionally grounded knowledge that is difficult to elicit through conventional qualitative approaches.

#### 4.1.2 Systems of Care and Structural Barriers

Photovoice consistently revealed survivor‐identified gaps in survivorship care, including fragmented systems, access barriers, and experiences of insensitive or dismissive care [53, 54]. These findings reinforce prior cancer‐focused reviews showing that photovoice is well‐suited to exposing structural inequities that remain underrepresented in clinical metrics and administrative data [49]. However, this body of work also highlights a critical limitation: few studies described mechanisms for translating photovoice‐generated insights into meaningful system‐level change [55, 56]. Without intentional integration into quality improvement, policy, or implementation efforts, photovoice risks remaining a diagnostic tool rather than a catalyst for reform. Future work should prioritize explicit pathways linking survivor‐generated evidence to decision‐making processes.

#### 4.1.3 Agency, Expression, and Advocacy

Across studies, photovoice was frequently associated with increased survivor agency, including reclaiming personal narratives, articulating future goals, and engaging in advocacy. These outcomes align with empowerment theory and mirror findings from prior reviews across diverse health contexts [11, 49, 52, 57, 58, 59]. However, empowerment was often framed as an individual‐level outcome, with limited attention to how survivor advocacy informed clinical practice, organizational change, or policy development. This gap suggests a need to shift photovoice research from demonstrating empowerment toward evaluating how empowered survivors can influence systems of care [60]. Embedding photovoice within participatory governance, clinical redesign, or policy translation frameworks may strengthen its impact beyond individual expression.

#### 4.1.4 Health Behavior and Lifestyle Change

Health behavior and lifestyle change emerged less frequently but represent an underdeveloped area of photovoice research in cancer survivorship. When examined, photovoice appeared to support survivors' reflection on daily routines, environmental constraints, and social influences shaping behaviors such as diet, physical activity, and self‐care [11, 45, 57, 61, 62]. These findings suggest that photovoice may complement traditional behavioral interventions by foregrounding contextual and structural determinants of health behaviors [49, 56]. However, few studies assessed sustained behavior change or integrated photovoice into longitudinal survivorship interventions, highlighting an opportunity for future research to examine photovoice as both an exploratory and intervention‐adjacent method.

### 4.2 Gaps in the Literature and Future Directions

The participant demographics in the reviewed studies highlight both the strengths and limitations of current photovoice literature. While studies spanned a wide age range, from infancy to older adulthood, most had small sample sizes (20 or fewer participants in 20 of the 26 studies). Although such numbers are methodologically consistent with photovoice and often sufficient to reach thematic saturation, smaller cohorts may still constrain the depth and consistency of analysis across diverse contexts [63]. Women and breast cancer survivors were disproportionately represented, whereas male survivors, individuals with less common cancer types, and those at varied treatment stages were often absent or underrepresented. Few studies included caregivers or healthcare providers, limiting opportunities to capture survivorship as a relational or systems‐level experience. These gaps point to a pressing need for greater inclusivity and transparency in future research designs.

Photovoice is an effective methodology for engaging marginalized or hard‐to‐reach groups, as its ability to blend visual and narrative formats enabled engagement across literacy levels and fostered rich emotional expression [18, 28]. However, methodological inconsistencies were typical in existing photovoice studies. Few studies discussed data saturation or analytic transparency, and the degree of participation varied widely. Some projects engaged participants in co‐analysis and community validation, while others remained largely researcher‐driven [9, 44]. Notably, very few studies involved participants in study design or interview guide development, despite this being a central part of participatory research. Other review studies also support these gaps, citing variability in sampling, analytic rigor, and participatory engagement [37, 40, 58].

Ethical rigor is central to photovoice in cancer survivorship because participant‐generated images can unintentionally reveal identities, sensitive health information, or third parties who did not consent to be photographed. Nonetheless, ethical protocols were reported inconsistently across studies. A dedicated ethics protocol should therefore be explicitly reported in future research, including (1) processes for informed and ongoing consent that distinguish consent to participate from consent to use, publish, and disseminate specific images and narratives; (2) safeguards for third‐party visibility (e.g., guidance on avoiding identifiable faces, obtaining written permission when others appear, and procedures for blurring/redaction when needed); and (3) clear agreements about image ownership, confidentiality, and advocacy use, including where and how photos may be shared (academic publications, social media, exhibitions, policy forums) and participants' right to withdraw selected images from dissemination. Because photovoice often aims to support advocacy, researchers should also address participant‐centered decision‐making about public display. Explicit ethical approaches can therefore strengthen transparency, protect autonomy and privacy, and align photovoice practice with its foundational participatory intent.

While these methodological and ethical limitations highlight inconsistencies, they may also reflect the practical challenges of implementing participatory methods. Limited resources, time constraints, and lack of training can hinder co‐analytic engagement or ethical rigor. Addressing these barriers will require investment in capacity‐building, such as training in community‐based research, institutional support for participatory approaches, and dedicated resources to ensure ethical, equitable implementation of photovoice studies.

Despite these limitations, expanding photovoice research into underrepresented survivorship populations offers a distinct methodological advantage by enabling survivors to *visually define* what survivorship means in their own contexts. However, photovoice remains underused within several important communities of note: adolescents and young adults (AYAs), older adults, LGBTQIA + individuals, racial and ethnic minorities, and people with disabilities continue to be underrepresented in this body of work [59, 60]. Research remains sparse in low‐resource and rural settings, despite photovoice's particular adaptability to such conditions. Finally, the absence of photovoice studies in low‐income countries reveals a significant geographic and economic gap in the current literature.

Unlike interview‐based approaches that rely primarily on retrospective verbal accounts, photovoice allows participants to capture everyday environments, relationships, and moments that shape survivorship but are often taken for granted or difficult to articulate. Among adolescents and young adults, photovoice can surface developmental disruptions and relational tensions related to cancer across school, home, and peer spaces, particularly during transitions between pediatric and adult systems of care. Among older adults, photovoice can illuminate how survivorship is negotiated alongside comorbidity, caregiving roles, and end‐of‐life meaning‐making within daily routines. In LGBTQIA+ and racially minoritized populations, the visual and participant‐controlled nature of photovoice creates space to document experiences of stigma, identity navigation, and resilience that may be silenced or constrained in traditional research settings, thereby generating context‐rich insights that are directly relevant to equity‐oriented care.

Furthermore, because survivorship experiences are shaped by geographic and policy environments [12], the current literature may overrepresent challenges more common in high‐income countries (HICs) while underreporting the structural barriers faced in lower‐resource settings. For example, while findings from HICs often focused on “return to work” and “identity”, the two studies conducted in lower‐middle‐income countries revealed distinct structural barriers, such as significant delays in diagnosis, misdiagnosis, and a lack of palliative care infrastructure. These disparities may also reflect barriers inherent to photo‐elicitation methods, including equipment costs, participants' availability, and digital literacy. To advance global health equity, future photovoice initiatives must prioritize low‐income countries, rural areas, and low‐resource settings to document locally driven strengths and structural barriers that HIC‐based care models may overlook.

### 4.3 Implications (Clinical and Research)

Photovoice is a participatory framework that allows clinicians, researchers, and healthcare systems to center survivors' voices in care planning, psychosocial support, and program development. When integrated into survivorship care, it can strengthen communication, support narrative‐based assessments, and encourage co‐creation of services. In clinical practice, photovoice can deepen support group discussions, inform survivorship care plans, and enhance provider training in empathy and patient‐centered care [11, 61].

The themes identified in this review point to several practical applications of photovoice. Survivors' frequent descriptions of identity disruption and emotional distress suggest that photovoice could be used as a reflective tool during psychosocial screening and counseling. Through visual storytelling, survivors can externalize complex emotions, providing clinicians with deeper insight into their experiences. Embedding photovoice into peer support groups, patient advisory boards, or co‐design initiatives can create meaningful opportunities for survivors to influence services through creative self‐representation [47].

Photovoice is well‐positioned to reach underserved communities, particularly in areas with limited access to healthcare or specialized healthcare. Highlighting survivor‐identified barriers and needs can guide quality improvement efforts and shape more responsive, equity‐driven care delivery. Additionally, using a sample from a specific community or area enables culturally tailored interventions, increasing acceptability and community trust.

At the systems and policy levels, Photovoice outputs, such as curated exhibits, community forums, and policy briefs, can enhance the dissemination of survivor perspectives and drive institutional change [12, 48]. To fully realize the potential of photovoice research, future studies should focus not only on generating survivor‐driven insights but also on developing strategies to embed those insights into programs, services, and system‐level reforms.

Recent work published after our final search date further reinforces the relevance of photovoice in survivorship research. For example, a 2025 photovoice study exploring women's body image following mastectomy [64] highlights the continued application of participatory visual methods to illuminate embodied, identity‐related survivorship experiences that are often underrepresented in traditional survey‐based research. Although published after the completion of our April 12, 2025 search window and therefore not included in the formal synthesis, this study underscores the ongoing expansion of photovoice methodologies within cancer survivorship scholarship and supports the trajectory identified in our review.

### 4.4 Study Limitations

This review was conducted using rigorous, transparent methods, including dual independent screening, narrative synthesis, and validated quality appraisal tools. Nonetheless, certain limitations should be acknowledged. Excluding non‐English and gray literature may have led to the omission of relevant studies. Additionally, heterogeneity in study design and variability in reporting complicated cross‐study comparisons limited the consistency with which outcomes could be synthesized.

## 5 Conclusion

Photovoice is a participatory method that captures the lived experiences of cancer survivors, particularly those of underrepresented individuals and communities. Across studies, survivors used photovoice to illuminate emotional and psychosocial challenges, structural barriers within systems of care, health behaviors, and pathways to empowerment and advocacy. Photovoice can translate survivor stories into actionable change, linking lived experience to systems‐level improvements and advancing survivorship care that is more responsive, equitable, and centered on underserved and marginalized communities. Future work should prioritize inclusive recruitment, rigorous participatory methodologies, transparent reporting, clear ethical protocols for visual and narrative data, and the evaluation of longer‐term outcomes.

## Funding

The authors have nothing to report.

## Conflicts of Interest

The authors declare no conflicts of interest.

## Supporting information


Supporting Information S1

## Data Availability

Data sharing not applicable to this article as no datasets were generated or analyzed during the current study.

## References

[1] World Health Organization . “Global Cancer Burden Growing, Amidst Mounting Need for Services,” (2024): [Internet], https://www.who.int/news/item/01‐02‐2024‐global‐cancer‐burden‐growing–amidst‐mounting‐need‐for‐services. PMC11115397 38438207

[2] National Cancer Institute . “Cancer Stat Facts: Cancer Survivorship,” (2022): [Internet], https://seer.cancer.gov/statfacts/html/survivor.html.

[3] National Cancer Institute . Division of Cancer Control and Population Sciences, Office of Cancer Survivorship (Definitions). [Internet] [cited Aug 21, 2025], https://cancercontrol.cancer.gov/ocs/definitions.

[4] M. Hewitt , S. Greenfield , and E. Stovall , eds., From Cancer Patient to Cancer Survivor: Lost in Transition (National Academies Press (US), 2006).

[5] D. K. Mayer , L. Nekhlyudov , C. F. Snyder , J. K. Merrill , D. S. Wollins , and L. N. Shulman , “American Society of Clinical Oncology Clinical Expert Statement on Cancer Survivorship Care Planning,” Journal of Oncology Practice 10, no. 6 (2014): 345–351, 10.1200/jop.2014.001321. 25316025

[6] L. Nekhlyudov , M. A. Mollica , P. B. Jacobsen , D. K. Mayer , L. N. Shulman , and A. M. Geiger , “Developing a Quality of Cancer Survivorship Care Framework: Implications for Clinical Care, Research, and Policy,” JNCI: Journal of the National Cancer Institute 111, no. 11 (November 2019): 1120–1130, 10.1093/jnci/djz089. 31095326 PMC6855988

[7] J. S. de Moor , A. B. Mariotto , C. Parry , et al., “Cancer Survivors in the United States: Prevalence Across the Survivorship Trajectory and Implications for Care,” Cancer Epidemiology, Biomarkers & Prevention 22, no. 4 (2013): 561–570, 10.1158/1055-9965.epi-12-1356. PMC3654837 23535024

[8] C. Wang and M. A. Burris , “Photovoice: Concept, Methodology, and Use for Participatory Needs Assessment,” Health Education & Behavior 24, no. 3 (1997): 369–387, 10.1177/109019819702400309. 9158980

[9] E. D. S. Lopez , E. Eng , E. Randall‐David , and N. Robinson , “Quality‐of‐Life Concerns of African American Breast Cancer Survivors Within Rural North Carolina,” Qualitative Health Research 15, no. 1 (2005): 99–115, 10.1177/1049732304270766. 15574718

[10] J. Yi , M. A. Kim , and S. H. Kim , “Health‐Related Quality of Life in Korean Young Adult Cancer Survivors: Effects of Age, Gender, and Cancer Site,” Qualitative Health Research 20, no. 8 (2010): 1070–1083, 10.1093/jjco/hyt187.

[11] C. Catalani and M. Minkler , “Photovoice: A Review of the Literature in Health and Public Health,” Health Education & Behavior 37, no. 3 (2010): 424–451, 10.1177/1090198109342084. 19797541

[12] C. L. Wong , H. Li , A. W. K. Leung , C. W. H. Chan , and Y. T. Cheung , “Understanding the Experience of Cancer Survivorship Among Pediatric and Adolescent Cancer Survivors and Their Parents Through Camera Lenses: A Photovoice Study,” Psycho‐Oncology 33, no. 9 (2024): e9306, 10.1002/pon.9306. 39191639

[13] M. J. Page , J. E. McKenzie , P. M. Bossuyt , et al., “The PRISMA 2020 Statement: An Updated Guideline for Reporting Systematic Reviews,” BMJ 372 (2021): n71, 10.1136/bmj.n71. 33782057 PMC8005924

[14] Covidence Systematic Review Software . Covidence (Version 2.0). [Computer software] [Internet], (Veritas Health Innovation, 2025), https://www.covidence.org/.

[15] M. Rodgers , A. Sowden , M. Petticrew , et al., “Testing Methodological Guidance on the Conduct of Narrative Synthesis in Systematic Reviews,” Evaluation 15, no. 1 (2009): 49–73, 10.1177/1356389008097871.

[16] J. Popay , H. Roberts , A. Sowden , et al., Guidance on the Conduct of Narrative Synthesis in Systematic Reviews: A Product from the ESRC Methods Programme (University of Lancaster, 2006).

[17] Critical Appraisal Skills Programme . CASP Qualitative Checklist [Internet], (2018), https://casp‐uk.net/casp‐tools‐checklists/.

[18] L. B. Edwards and L. E. Greeff , “Evidence‐Based Feedback About Emotional Cancer Challenges Experienced in South Africa: A Qualitative Analysis of 316 Photovoice Interviews,” Global Public Health 13, no. 10 (October 2018): 1409–1421, 10.1080/17441692.2017.1357187. 28776485

[19] L. B. Edwards and L. E. Greeff , “Exploring Grassroots Feedback About Cancer Challenges in South Africa: A Discussion of Themes Derived From Content Thematic Analysis of 316 Photo‐Narratives,” Pan African Medical Journal 28 (2017): 173, 10.11604/pamj.2017.28.173.11894. 29541319 PMC5847259

[20] L. B. Edwards and L. E. Greeff , “A Descriptive Qualitative Study of Childhood Cancer Challenges in South Africa: Thematic Analysis of 68 Photovoice Contributions,” South African Journal of Oncology 1, no. 1 (January 2017): 1–8, 10.4102/sajo.v1i0.14.

[21] T. L. Morrison and R. L. Thomas , “Comparing Men’s and Women’s Experiences of Work After Cancer: A Photovoice Study,” Supportive Care in Cancer 23, no. 10 (2015): 3015–3023, 10.1007/s00520-015-2670-4. 25739752

[22] T. L. Morrison and R. L. Thomas , “Survivors’ Experiences of Return to Work Following Cancer: A Photovoice Study,” Canadian Journal of Occupational Therapy 81, no. 3 (2014): 163–172, 10.1177/0008417414534398. 25154130

[23] T. L. Morrison and R. L. Thomas , “Cancer Survivors’ Concealment or Disclosure of Diagnosis: Implications for Return to Work,” Work Read Mass 52, no. 3 (2015): 643–655, 10.3233/wor-152120. 26409365

[24] M. J. Bates , T. Mphwatiwa , J. Ardrey , N. Desmond , L. W. Niessen , and S. B. Squire , “Household Concepts of Wellbeing and the Contribution of Palliative Care in the Context of Advanced Cancer: A Photovoice Study From Blantyre, Malawi,” PLoS One 13, no. 8 (2018): 1–18, 10.1371/journal.pone.0202490. PMC6104988 30133511

[25] Z. M. Bood , F. van Liemt , M. A. G. Sprangers , et al., “This is what Life With Cancer Looks Like: Exploring Experiences of Adolescent and Young Adults With Cancer Using Two Visual Approaches,” Supportive Care in Cancer 30, no. 4 (April 2022): 3353–3361, 10.1007/s00520-021-06775-9. 34988705 PMC8730754

[26] C. Capewell , S. Ralph , and M. Symonds , “Listening to Women’s Voices: Using an Adapted Photovoice Methodology to Access Their Emotional Responses to Diagnosis and Treatment of Breast Cancer,” Journal of Patient Experience 7, no. 6 (2020): 1316–1323, 10.1177/2374373520930463. 33457581 PMC7786681

[27] J. Currin‐McCulloch , D. Peterson , S. Kaushik , and J. Borrego , “Through the Lens: The Feasibility and Acceptability of an Online Meaning‐Making Photovoice Group Among Young Adult Cancer Survivors,” Social Work with Groups 47, no. 1 (2024): 47–63, 10.1080/01609513.2023.2212391.

[28] F. Ebrahimpour , J. Mirlashari , A. S. S. Hosseini , F. Zarani , and S. Thorne , “Symbols of Hope on Pediatric Oncology Ward: Children’s Perspective Using Photovoice,” Journal of Pediatric Oncology Nursing 38, no. 6 (2021): 385–398, 10.1177/10434542211041934. 34541954

[29] B. J. Zebrack , “Psychological, Social, and Behavioral Issues for Young Adults With Cancer,” supplement, Cancer 117, no. S10 (2011): 2289–2294, 10.1002/cncr.26056. 21523748

[30] G. Georgievski , W. Shama , S. Lucchetta , and M. Niepage , “Through Our Eyes: A Photovoice Intervention for Adolescents on Active Cancer Treatment,” Journal of Psychosocial Oncology 36, no. 6 (2018): 700–716, 10.1080/07347332.2018.1469564. 30372377

[31] C. Hammond , R. Thomas , W. Gifford , et al., “Cycles of Silence: First Nations Women Overcoming Social and Historical Barriers in Supportive Cancer Care,” Psycho‐Oncology 26, no. 2 (February 2017): 191–198, 10.1002/pon.4335. 27935147

[32] P. Jellema , M. Annemans , A. Heylighen , et al., “At Home in the Hospital and Hospitalised at Home: Exploring Experiences of Cancer Care Environments,” in Breaking down Barriers (Springer International Publishing AG, 2018), 215–226.

[33] P. Jellema , M. Annemans , and A. Heylighen , “The Roles of Cancer Care Facilities in Users’ Well‐Being,” Building Research & Information 48, no. 3 (April 2020): 254–268, 10.1080/09613218.2019.1620094.

[34] E. D. S. Lopez , E. Eng , E. Randall‐David , and N. Robinson , “Quality‐of‐Life Concerns of African American Breast Cancer Survivors Within Rural North Carolina: Blending the Techniques of Photovoice and Grounded Theory,” Qualitative Health Research 15, no. 1 (2005): 99–115, 10.1177/1049732304270766. 15574718

[35] World Bank . World Bank Country and Lending Groups – World Bank Data Help Desk (World Bank, 2024): [Internet], https://datahelpdesk.worldbank.org/knowledgebase/articles/906519‐world‐bank‐country‐and‐lending‐groups.

[36] M. Mosavel and K. D. Sanders , “Photovoice: A Needs Assessment of African American Cancer Survivors,” Journal of Psychosocial Oncology 28, no. 6 (2010): 630–643, 10.1080/07347332.2010.516809. 21058160

[37] N. O’Callaghan , P. Douglas , and L. Keaver , “Meaning of Nutrition for Cancer Survivors: A Photovoice Study,” BMJ Nutrition, Prevention & Health 7, no. 1 (2024): 112–118, 10.1136/bmjnph-2023-000822. PMC11221309 38966113

[38] M. E. Pailler , L. K. Beaupin , E. Brewer‐Spritzer , et al., “Reaching Adolescent and Young Adult Cancer Patients Through Social Media: Impact of the Photographs of Meaning Program,” Journal of Adolescent and Young Adult Oncology 9, no. 4 (2020): 508–513, 10.1089/jayao.2019.0140. 32255697 PMC7640743

[39] J. S. Park , J. W. Han , J. H. Choi , and K. C. Lee , “Photovoice‐Based Assessment of Weight Management Experiences of Breast Cancer Patients Treated With Tamoxifen,” International Journal of Environmental Research and Public Health 17, no. 12 (2020): 4359, 10.3390/ijerph17124359. 32570717 PMC7345025

[40] J. Poudrier and R. T. Mac‐Lean , ““We’ve Fallen into the Cracks”: Aboriginal Women’s Experiences With Breast Cancer Through Photovoice,” Nursing Inquiry 16, no. 4 (2009): 306–317, 10.1111/j.1440-1800.2009.00435.x. 19906281

[41] R. Power , J. M. Ussher , J. Perz , K. Allison , and A. J. Hawkey , ““Surviving Discrimination by Pulling Together”: LGBTQI Cancer Patient and Carer Experiences of Minority Stress and Social Support,” Frontiers in Oncology 12 (2022): 918016, 10.3389/fonc.2022.918016. 35814403 PMC9263127

[42] S. S. Wong , T. J. George , M. Godfrey , J. Le , D. B. Pereira , and T. J. George Jr , “Using Photography to Explore Psychological Distress in Patients With Pancreatic Cancer and Their Caregivers: A Qualitative Study,” Supportive Care in Cancer 27, no. 1 (2019): 321–328, 10.1007/s00520-018-4330-y. 29959574 PMC6289888

[43] J. Yi , M. A. Kim , and S. An , “The Experiences of Korean Young Adult Survivors of Childhood Cancer: A Photovoice Study,” Qualitative Health Research 26, no. 8 (2016): 1044–1054, 10.1177/1049732315599374. 26265716

[44] J. Yi and B. Zebrack , “Self‐Portraits of Families With Young Adult Cancer Survivors: Using Photovoice,” Journal of Psychosocial Oncology 28, no. 3 (May 2010): 219–243, 10.1080/07347331003678329. 20432114 PMC2862598

[45] T. A. Baker and C. C. Wang , “Photovoice: Use of a Participatory Action Research Method to Explore the Chronic Pain Experience in Older Adults,” Qualitative Health Research 16, no. 10 (2006): 1405–1413, 10.1177/1049732306294118. 17079801

[46] A. Lanoye , L. Cai , M. D. Thomson , and S. Hong , “Use of Photo Methods in Research Studies With Cancer Survivors and Their Caregivers: A Scoping Review,” Journal of Cancer Survivorship 18, no. 3 (June 2024): 698–709, 10.1007/s11764-022-01321-w. 36567405

[47] Y. Yang , “How to Conduct a Photovoice Systematic Review: Lessons Learned and Recommendations,” Qualitative Report 28, no. 4 (2023): 979–990, 10.46743/2160-3715/2023.5792.

[48] M. Quinn , N. Wright , M. Scherdt , et al., “A Descriptive Study of Policy and System‐Level Interventions to Address Cancer Survivorship Issues Across Six United States Health Systems,” Journal of Cancer Survivorship 18, no. 6 (2024): 2022–2032, 10.1007/s11764-023-01440-y. 37544977

[49] A. Lanoye , L. Cai , M. D. Thomson , and S. Hong , “Use of Photo Methods in Research Studies With Cancer Survivors and Their Caregivers,” Journal of Cancer Survivorship 18, no. 3 (2024): 698–709, 10.1007/s11764-022-01321-w. 36567405

[50] M. Niepage , G. Georgievski , W. Shama , and S. Lucchetta , “Exploring Adolescents' Cancer Journey Through Photovoice: A Narrative Synthesis,” Journal of Adolescent and Young Adult Oncology 7, no. 1 (2018): 15–21, 10.1089/jayao.2017.0073. 29022770

[51] T. A. R. Evangelista , M. D. R. Nunes , S. G. Machado , M. A. Santos , B. C. D. Olegário , and L. C. Nascimento , “Use of Photography in Research With Children and Adolescents With Chronic Condition: An Integrative Review,” Acta Paulista de Enfermagem 36 (2023): eAPE01994, https://acta‐ape.org/en/article/use‐of‐photography‐in‐research‐with‐children‐and‐adolescents‐with‐chronic‐conditions‐an‐integrative‐review/.

[52] M. Stephens , E. Keiller , M. Conneely , P. Heritage , M. Steffen , and V. J. Bird , “A Systematic Scoping Review of Photovoice Within Mental Health Research Involving Adolescents,” International Journal of Adolescence and Youth 28, no. 1 (2023), 10.1080/02673843.2023.2244043.

[53] M. Charlton , J. Schlichting , C. Chioreso , M. Ward , and P. Vikas , “Challenges of Rural Cancer Care in the United States,” Oncology Williston Park 29, no. 9 (2015): 633–640, https://www.cancernetwork.com/view/challenges‐rural‐cancer‐care‐united‐states. 26384798

[54] T. Onega , E. J. Duell , X. Shi , D. Wang , E. Demidenko , and D. Goodman , “Geographic Access to Cancer Care in the U.S,” Cancer 112, no. 4 (2008): 909–918, 10.1002/cncr.23229. 18189295

[55] K. Halvorsrud , O. Eylem , R. Mooney , M. Haarmans , and K. Bhui , “Identifying Evidence of the Effectiveness of Photovoice: A Systematic Review and Meta‐Analysis of the International Healthcare Literature,” Journal of Public Health (Oxford, England) 44, no. 3 (2022): 704–712, 10.1093/pubmed/fdab074. 33823022 PMC9424055

[56] L. Tang , J. Gu , and Z. Lin , “A Scoping Review of Photovoice for People Living With Diabetes,” BMC Public Health 25, no. 1 (2025): 540, 10.1186/s12889-025-21410-6. 39930389 PMC11808938

[57] C. Wang and M. A. Burris , “Photovoice: Concept, Methodology, and Use for Participatory Needs Assessment,” Health Education & Behavior 24, no. 3 (1997): 369–387, 10.1177/109019819702400309. 9158980

[58] K. Anderson , E. Elder‐Robinson , K. Howard , and G. Garvey , “A Systematic Methods Review of Photovoice Research With Indigenous Young People,” International Journal of Qualitative Methods 22 (2023), 10.1177/16094069231172076.

[59] D. Chinn and B. Balota , “A Systematic Review of Photovoice Research Methods With People With Intellectual Disabilities,” Journal of Applied Research in Intellectual Disabilities 36, no. 4 (2023): 725–738, 10.1111/jar.13106. 37062820

[60] B. Palibroda and others , A Practical Guide to Photovoice: Sharing Pictures, Telling Stories and Changing Communities (Prairie Women’s Health Centre of Excellence, 2009).

[61] N. E. Findholt , Y. L. Michael , M. M. Davis , and V. W. Brogoitti , “Environmental Influences on Children’s Physical Activity and Eating: A Qualitative Study Using Photovoice,” Health Promotion Practice 12, no. 5 (2011): 705–713, 10.4278/ajhp.100622-QUAL-210. 22040399

[62] K. Budig , J. Diez , P. Conde , M. Sastre , M. Hernán , and M. Franco , “Photovoice and Empowerment: Evaluating the Transformative Potential of a Participatory Action Research Project,” BMC Public Health 18, no. 1 (2018): 432, 10.1186/s12889-018-5335-7. 29609576 PMC5879794

[63] J. H. Rowland , E. E. Kent , L. P. Forsythe , et al., “Cancer Survivorship Research in Europe and the United States: Where Have We Been, Where Are We Going, and What Can We Learn From Each Other?,” supplement, Cancer 119, no. S11 (2013): 2094–2108, 10.1002/cncr.28060. 23695922 PMC3690309

[64] Y. Erden , H. C. Celik , and N. Karahurt , “Women’s Body Image After Mastectomy: A Photovoice Study,” Supportive Care in Cancer 33, no. 6 (2025): 501, 10.1007/s00520-025-09541-3. 40423833 PMC12116761

