# Supplementary material for: Photovoice Utilization for Research in Cancer Survivors: A Systematic Review
Source: Psychooncology. 2026 Mar 18;35(3):e70431. doi: 10.1002/pon.70431 (PMC12997390; doi:10.1002/pon.70431)
Supplement: Supplementary file 1 — Supporting Information S1 [file PON-35-e70431-s001.docx]

**Appendix A1**

**Search String**

| Concept | Search Terms * |
| --- | --- |
| Photovoice | Photovoice OR Photo Voice |
| Cancer | Cancer* OR oncology OR malignan*OR neoplasm OR tumor* OR tumour* OR leukemia* OR lymphom* OR melanom* OR sarcom* |
| Study Participants | Patient OR survivor |

**Appendix A2**

**Inclusion/Exclusion Criteria**

| Category | Inclusion | Exclusion |
| --- | --- | --- |
| Population | Cancer diagnosis | No cancer diagnosis  Only caregivers  Only healthcare workers  Not able to separate results of survivors from other participants |
| Geography | No limits | n/a |
| Time period | No limits | n/a |
| Settings | No limits | n/a |
| Research Methods | Photovoice methodology, photos and narratives | Only photos  Only narratives  Not able to separate out photovoice outcomes from other methodology used |
| Language | English, or translated into English | Not in English, or no English translation |
| Admissible evidence | Qualitative studies  Randomized control trials  Nonrandomized trials  Single group pre/post | Reviews (systematic or nonsystematic)  Editorials  Letters to the editor  Theses  Dissertations |

# **Appendix A3. CASP Qualitative Checklist: Short-Hand and Full Questions**

| **Shorthand** | **Full Question** |
| --- | --- |
| Q1: Qual. Approp. | Was there a clear statement of the aims of the research? |
| Q2: Aim Clear | Is a qualitative methodology appropriate? |
| Q3: Rigorous Design | Was the research design appropriate to address the aims of the research? |
| Q4: Data Collection | Was the recruitment strategy appropriate to the aims of the research? |
| Q5: Researcher Role | Was the data collected in a way that addressed the research issue? |
| Q6: Context Clear | Has the relationship between researcher and participants been adequately considered? |
| Q7: Reliable Methods | Have ethical issues been taken into consideration? |
| Q8: Rigorous Analysis | Was the data analysis sufficiently rigorous? |
| Q9: Rich Data | Is there a clear statement of findings? |
| Q10: Reliable Analysis | Is the research valuable? |
| Q11: Convincing Findings | Are the findings convincing and supported by the data? |
| Q12: Findings Relevant | Are the findings relevant to the study’s aims and context? |
| Q13: Conclusions | Were the conclusions clearly supported by the findings? |
| Q14: Ethics Reported | How clear and coherent is the reporting of ethics? |

| **Appendix A4: Appraisal Results with CASP Qualitative Checklist Items** | | | | | | | | | | | | | | | | | | | | | | | | | | | | |
| --- | --- | --- | --- | --- | --- | --- | --- | --- | --- | --- | --- | --- | --- | --- | --- | --- | --- | --- | --- | --- | --- | --- | --- | --- | --- | --- | --- | --- |
| **Author (Year)Title** | **1.Qual App** | | **2. Aim clear** | **3. Rigorous Design** | | **4. Data collection** | | | **5. Researcher role** | | **6. Context clear** | **7. Reliable Methods** | | | **8. Rigorous analysis** | | **9. Rich Data** | | **10. Reliable Analysis** | **11. Convincing Findings** | **12. Findings Relevant** | | | **13. Conclusions** | **14. Ethics Reported** | | | **Overall rating** |
| Bates_2019 | Appropriate | Clear | | | Defensible | | Appropriately | Clearly described | | Clear | | | Reliable | Rigorous | | Rich | | Reliable | | Convincing | | Relevant | Adequate | | | Appropriate | ++ | |
| Bood_2022 | Appropriate | Clear | | | Defensible | | Appropriately | Clearly described | | Clear | | | Reliable | Rigorous | | Rich | | Reliable | | Convincing | | Relevant | Adequate | | | Appropriate | ++ | |
| Capewell_2020 | Appropriate | Clear | | | Defensible | | Appropriately | Clearly described | | Clear | | | Reliable | Rigorous | | Rich | | Reliable | | Convincing | | Relevant | Adequate | | | Appropriate | ++ | |
| Curin_McCulloch_2024 | Appropriate | Clear | | | Defensible | | Appropriately | Unclear | | Clear | | | Reliable | Rigorous | | Rich | | Reliable | | Convincing | | Relevant | Adequate | | | Appropriate | ++ | |
| Ebrahimpour_2021 | Appropriate | Clear | | | Defensible | | Appropriately | Clearly described | | Clear | | | Reliable | Rigorous | | Rich | | Reliable | | Convincing | | Relevant | Adequate | | | Appropriate | ++ | |
| Edwards_2017_child | Appropriate | Clear | | | Defensible | | Appropriately | Clearly described | | Clear | | | Reliable | Rigorous | | Rich | | Reliable | | Convincing | | Relevant | Adequate | | | Appropriate | ++ | |
| Edwards_2018_grassroots | Appropriate | Clear | | | Defensible | | Appropriately | Clearly described | | Clear | | | Reliable | Rigorous | | Rich | | Reliable | | Convincing | | Relevant | Adequate | | | Appropriate | ++ | |
| Edwards_2018 | Appropriate | Clear | | | Defensible | | Appropriately | Clearly described | | Clear | | | Reliable | Rigorous | | Rich | | Reliable | | Convincing | | Relevant | Adequate | | | Appropriate | ++ | |
| Georgievski_2018 | Appropriate | Clear | | | Defensible | | Appropriately | Clearly described | | Clear | | | Reliable | Rigorous | | Rich | | Reliable | | Convincing | | Relevant | Adequate | | | Appropriate | ++ | |
| Hammond_2016 | Appropriate | Clear | | | Defensible | | Appropriately | Clearly described | | Clear | | | Reliable | Rigorous | | Rich | | Reliable | | Convincing | | Relevant | Adequate | | | Appropriate | ++ | |
| Jellema_2018 | Appropriate | Clear | | | Defensible | | Appropriately | Unclear | | Clear | | | Reliable | Rigorous | | Rich | | Reliable | | Convincing | | Relevant | Adequate | | | Appropriate | ++ | |
| Jellema_2020 | Appropriate | Clear | | | Defensible | | Appropriately | Unclear | | Clear | | | Reliable | Rigorous | | Rich | | Reliable | | Convincing | | Relevant | Adequate | | | Appropriate | ++ | |
| Lopez_2005 | Appropriate | Clear | | | Defensible | | Appropriately | Clearly described | | Clear | | | Reliable | Rigorous | | Rich | | Reliable | | Convincing | | Relevant | Adequate | | | Appropriate | ++ | |
| Morrison_2014 | Appropriate | Clear | | | Defensible | | Appropriately | Clearly described | | Clear | | | Reliable | Rigorous | | Rich | | Reliable | | Convincing | | Relevant | Adequate | | | Appropriate | ++ | |
| Morrison_2015_comparing | Appropriate | Clear | | | Defensible | | Appropriately | Clearly described | | Clear | | | Reliable | Rigorous | | Rich | | Reliable | | Convincing | | Relevant | Adequate | | | Appropriate | ++ | |
| Morrison_2015_concelement | Appropriate | Clear | | | Defensible | | Appropriately | Clearly described | | Clear | | | Reliable | Rigorous | | Rich | | Reliable | | Convincing | | Relevant | Adequate | | | Appropriate | ++ | |
| Mosavel_2010 | Appropriate | Clear | | | Defensible | | Appropriately | Clearly described | | Clear | | | Reliable | Rigorous | | Rich | | Reliable | | Convincing | | Relevant | Adequate | | | Appropriate | ++ | |
| O'Callaghan_2024 | Appropriate | Clear | | | Defensible | | Appropriately | Unclear | | Clear | | | Reliable | Rigorous | | Rich | | Reliable | | Convincing | | Relevant | Adequate | | | Appropriate | ++ | |
| Pailler_2020 | Appropriate | Clear | | | Defensible | | Appropriately | Unclear | | Clear | | | Reliable | Rigorous | | Rich | | Reliable | | Convincing | | Relevant | Adequate | | | Appropriate | ++ | |
| Park_2020 | Appropriate | Clear | | | Defensible | | Appropriately | Unclear | | Clear | | | Reliable | Rigorous | | Rich | | Reliable | | Convincing | | Relevant | Adequate | | | Appropriate | ++ | |
| Poudrier_2009 | Appropriate | Clear | | | Defensible | | Appropriately | Unclear | | Clear | | | Reliable | Rigorous | | Rich | | Reliable | | Convincing | | Relevant | Adequate | | | Appropriate | ++ | |
| Power_2022 | Appropriate | Clear | | | Defensible | | Appropriately | Clearly described | | Clear | | | Reliable | Rigorous | | Rich | | Reliable | | Convincing | | Relevant | Adequate | | | Appropriate | ++ | |
| Wong_2019 | Appropriate | Clear | | | Defensible | | Appropriately | Clearly described | | Clear | | | Reliable | Rigorous | | Rich | | Reliable | | Convincing | | Relevant | Adequate | | | Appropriate | ++ | |
| Wong_2024 | Appropriate | Clear | | | Defensible | | Appropriately | Clearly described | | Clear | | | Reliable | Rigorous | | Rich | | Reliable | | Convincing | | Relevant | Adequate | | | Appropriate | ++ | |
| Yi_2010 | Appropriate | Clear | | | Defensible | | Appropriately | Clearly described | | Clear | | | Reliable | Rigorous | | Rich | | Reliable | | Convincing | | Relevant | Adequate | | | Appropriate | ++ | |
| Yi_2019 | Appropriate | Clear | | | Defensible | | Appropriately | Clearly described | | Clear | | | Reliable | Rigorous | | Rich | | Reliable | | Convincing | | Relevant | Adequate | | | Appropriate | ++ | |

**Footnote:** Grayed-out boxes indicate a single CASP checklist item rated as "No" or "Unclear," suggesting a potential methodological limitation. However, since no study had more than one such rating, all studies were deemed to be of sufficient overall quality and were included in the review.

**Appendix A5: Photovoice Study Data n=26 studies**

| **First Author**    **Year Published**  **Title/Journal** | **Recruitment/Eligibility** | **Type of Study**    **Study Design, Procedures, Intervention (If applicable), Analysis methods** | **Location of Study**    **Aims/Research Questions** | **Method of Measurement and Analysis**    **Research Variables** |
| --- | --- | --- | --- | --- |
| Bates, M.J.    2018    Household Concepts of Wellbeing and the Contribution of Palliative Care in the Context of Advanced Cancer: A Photovoice Study from Blantyre, Malawi    PLOS ONE | All households in which a patient was receiving palliative care from Tiyanjane clinic for a diagnosis of advanced cancer within the catchment of Ndirande health centre were considered eligible. At the start of the study this comprised sixteen households, all of whom were approached in advance of the study by the community palliative care nurse. | Qualitative    Photovoice, qualitative coding using deductive thematic analysis    Participants are considered “co-researchers (i.e. patients and family caregivers)”. A two-stage consent process was used for co-researchers: at the start of the study (for participation) and towards the end of the study (relating to disclosure and sharing of photographic material).    First group meeting: given digital cameras and received basic training. Over one month, gathered images on “the story of my illness”. Images selected during one-to-one sessions before each group session and selected photos were printed as hard copy to be used for participatory analysis in each session. Researchers took field notes during the sessions. | Blantyre, Malawi    Urban and Peri-urban    Outpatient (not specified)    To explore how patients with advanced cancer and their caregivers define wellbeing and the role of palliative care in supporting their quality of life | Participatory analysis: occurred in 7/9 group sessions. First phase (5 sessions), researchers and co-researchers sorted through and group photos and name these categories. Then used SHOWeD prompt to further explore the categories. Towards the end of the first month through reviewing images and reading and re-reading categories aloud as a co-researcher group, categories were brought together into named themes.  Separate exercise (2 sessions): co-researchers individually selected photos to compose a short written piece (captioning) and photos are displayed in a photo exhibition at an advocacy event, that included local health and community leaders, at the end of the data collection period.    Secondary deductive analysis based on the themes named by co-researchers were done on transcripts |
| Bood, Z.M.    2022    This is What Life with Cancer Looks Like: Exploring Experiences of Adolescent and Young Adults with Cancer Using Two Visual Approaches    Supportive Care in Cancer | AYAs (18–35 years old) who had cancer, or were in remission from cancer, could apply to the photovoice project by sending an introductory text about themselves to the F\|FortFoundation. The project was promoted on social media, and on the website of the F\|FortFoundation and partner organizations | Qualitative    Used Rich pictures and photovoice    Six relevant domains were identified in a brainstorm session, organized by F\|FortFoundation. AYAs make one photo for each domain: (1) the essence: “This is the pure definition of me”; (2) the wrong gear: “I am pushing the accelerator pedal, but I am not moving. I cannot keep up with my peers”; (3) concerns about the future: “What will happen with me after this treatment?”; (4) The remedy: “What I need to be able to handle the toughest moments of my treatment”; (5) Forms of interaction: “I have to take control over the interactions between me and others.”; and (6) The end: “What does death actually mean and how will dying look like for me?.”    All participants worked with a photographer for two days. A week before meeting the photographer, the participant was interviewed by FvL about how the six domains played a role in his/her life. After the interview, FvL, together with the photographer, developed a mood board based on the interview answers as preparation for the photography days. On the first photography day, FvL, the photographer, and the participant discussed the mood board and determined composition and location for the photos. The rest of the two days were used to make six photos, one for each domain. Photos could be portraits, still lives, landscapes, or any other composition. Each photo was accompanied by a caption, written by the F\|FortFoundation and approved by the participants. F\|FortFoundation developed a book with all the photos. | Netherlands    No specified    Outpatient    To explore how AYA cancer patients and survivors experience life with cancer using visual storytelling techniques. | RPs and photos were first analyzed separately. Each photo was analyzed separately, by looking at what was in the photo and what was written in the caption. The first author, ZB, described what could be seen on the photo and then applied codes to the photo and the caption. After all photos were analyzed, the codes were clustered into subthemes and main themes. ZB discussed this process and the emerging themes with EH. Subsequently, ZB and EH compared the themes that emerged from the RPs to ones in the photos, explicitly looking for similar or different metaphors and visual motifs. |
| Capewell, C.    2020    Listening to Women’s Voices: Using an Adapted Photovoice Methodology to Access Their Emotional Responses to Diagnosis and Treatment of Breast Cancer    Journal of Patient Experience | Recruitment was through a snowballing technique using the researchers’ social networks. The selection criteria were women with a primary diagnosis of breast cancer | Qualitative  Two meetings were held with each group. At an initial meeting, the researchers briefed participants on the aim of the study, including discussing ethical issues. Participants were asked to create images of their experience and to write a short explanation of what the image represented to them (the photo and voice—words—elements of photovoice). Questions by the participants about the task led to examples being generated by them. An image of a letter was considered along with words to explain it. Unlike other photovoice projects, the participants were not provided with disposable cameras nor were specific questions set neither was the type and number of images/words proscribed. This was part of the adaptation of exploring the experience and trying to demonstrate the participant-led nature of the data generation. The format  participants produced of images/words was varied. Some chose to create a narrative of their experience illustrated with images, either hand-drawn or downloaded from the internet. Others provided a few pictures cut out of magazines or newspapers to which they gave captions.  There was a time lag of 2 weeks before a second meeting was held with the same 3 groups. The second meeting  was audio recorded. Each group began by participants being asked if anyone would like to share any of their images and captions. The women’s words led to the theme with a quote used which best articulated its concept. This adaptation of photovoice with a participant-led discussion meant the topics covered were those of importance and relevance to them. They tended to facilitate the group and include everyone with the researchers only asking for clarification, usually around treatment aspects and drug names. | United Kingdom    Urban    Outpatient    The research aim was: to gain insight into how women perceived their experiences of diagnosis and treatment of breast cancer to better inform health care professionals. The  research questions are:  1. What were the women’s experience of diagnosis and treatment?  2. What was perceived positively?  3. What was perceived negatively?  4. What recommendations would the women make to improve their experience? | Analysis based on the theoretical  underpinning of community-based participatory research. The photovoice path (2) was covered with all participants being involved in individual image/narrative construction then sharing and reflecting. Analysis followed thematic analysis. Those participants, who were willing to do so, reviewed the themes and relevant images. They endorsed the themes as relating to their experience and that the specific image related to their group discussion. The original caption of the  image reflected their experience. |
| Currin-McCulloch, J.    2024    Through the Lens: The Feasibility and Acceptability of an Online Meaning-Making Photovoice Group Among Young Adult Cancer Survivors    Social Work with Groups | young adults (18-45), who were diagnosed with cancer and on active treatment, or within 5 years of treatment completion. Participants needed to have access to the internet and the ability to take and share photographs Lastly, they had to be able to speak, read, and write English. The study’s exclusion criteria entailed participants who had non melanoma skin cancers, which seldom require extended exposure to cancer treatment and the associated mental and physical strain, or those with cognitive impairments | Qualitative    (a) individual pre-group orientations, (b) 8-week intervention, virtual group weekly session of 1.5 hours, weekly post of one photo with caption (c) optional participation in a photo exhibit where participants choose two photographs from their project to display along with other group members’ photographs, and (d) individual exit interviews  Participants choose to share one or two photographs with the group at each weekly group session. These were photographs that they took of themselves, objects, significant people in their lives, or images others took. Participants received prompts prior to each group session that aided their decision-making process in choosing which photos to take/share. Weekly topics included: “All about me;” “My life with cancer;” “What I value in life;” “My identity;” “Honoring my accomplishments;” “Fostering my resiliency;” “Creating my legacy;” and “Finding hope in uncertainty.” Members were also asked to create a brief (4–6 sentences) caption that would serve as a description of how the image addressed the weekly topic. | Online in the United States    Not specified    Outpatient    To evaluate the feasibility, acceptability, and benefit of an 8-week synchronous online meaning-making photovoice group work intervention among a YA cancer survivor sample | Dependent Variables:  Feasibility (the number of participants recruited, the time it took to reach the desired sample size, attendance at the 8-weekly sessions, retention for the entire 8-week intervention.)  Acceptability (The exit interviews incorporated a structured interview format to gather participants’ views about the acceptability of the intervention, including barriers and facilitators to participation, as well as their perceived benefits of group participation. Individual exit interviews took place one to 3 weeks following the last group session)    Descriptive statistics were utilized to calculate the number of group participants, the time required to achieve the desired participant sample, attendance at weekly sessions, and retention in the intervention. To summarize the YAs’ perspectives of the acceptability of the TTL intervention, the authors engaged applied thematic analysis methods. |
| Ebrahimpour, F.    2021    Symbols of Hope on Pediatric Oncology Ward: Children’s Perspective Using Photovoice    Journal of Pediatric Oncology | The inclusion criteria were: (1) children hospitalized with cancer; (2) six to 12 years of age; (3) ability to speak and understand Persian; (4) willingness to participate; and (5) ability to use the camera. The exclusion criteria were the child’s unwillingness to participate or a level of health that precluded participation. | Qualitative  children were taught how to use the camera (researcher’s phone, which had no SIM card in it). The children were then asked to act as collaborators or  detectives, investigate around the ward, and take photos of what gave them a sense of hope. There was no restriction on the number of photos or session length, and all children took photos without their caregivers’ help. On average, the duration was less than 15 min. . | Tehran, Iran    Urban    Inpatient    To explore how hospitalized children with cancer perceive hope and identify factors that foster hope in an oncology ward. | Shortly after taking the photos, semi-structured interviews were conducted in a private area. We used the SHOWeD approach. Four questions were asked about each photo: (1) Can you explain what this photo shows? (2) Why did you choose this scene? (3) What is the relationship between this photo and your sense of hope? (4) Is there anything else about this photo that you want to express? The interview sessions lasted 10-30 min. Parents were  not present during the interviews    A thematic analysis approach |
| Edwards, L. B.    2017    A Descriptive Qualitative Study of Childhood Cancer Challenges in South Africa    South African Journal of Oncology | A total of 58 parents and guardians of children with cancer, childhood cancer patients, and 10 paediatric oncology workers were selected via convenience sampling. | Qualitative    All participants were interviewed via face-to-face interviews using photovoice methodology. Two openended questions were asked (Question 1: What would you like to say about having cancer or about someone close to you who has had cancer? Question 2: Are there specific problems that you think need to be spoken about so that things can be improved?). Participants were invited to choose a photographic image that could represent their story; cameras were provided and participants were supported through the playful activity of taking a photograph that visually referred to their story. The photograph was then combined with the documented photo-narrative to form a set of advocacy materials.. | 41 different towns of  residence across South Africa representing 9 regions (Eastern Cape: 16; Free State: 4; Gauteng: 13; Kwazulu-Natal: 15; Lesotho: 2; Limpopo: 5; North West: 7; Northern Cape: 2; and Western Cape: 4).  Rural/Urban  In/outpatient  The aim of this study was to document and thematically analyse photovoice contributions of childhood cancer-related challenges in SA, to promote understanding of the childhood cancer care landscape and for photovoice contributions to be used for cancer advocacy | Content analysis and inductive thematic encoding |
| Edwards 2017  Exploring grassroots feedback about cancer challenges in South Africa: a discussion of themes derived from content thematic analysis of 316 photo-narratives | From 2014 to 2016, 286 cancer patients and family members (called patient participants) and 30 health care participants were selected using convenience sampling from tertiary oncology units and cancer interim homes across SA. | All participants were interviewed via face-to-face interviews using photovoice methodology and a semi-structured interview.  Responses to 2 open-ended research questions (i.e. 1. Did you experience cancer challenges, and if so what were they? 2. Are there any specific cancer challenges that you think need urgent advocacy?) were documented as colloquial narratives (called photo-narratives). Cameras were provided by the interviewers and participants were assisted in the taking, titling and explaining of the meaning of their photograph. The title, reason for choosing the photograph and the actual photographic image were then combined with the documented photo-narrative to become advocacy material. Advocacy material was then available for use in projects such as breast cancer exhibitions, as cancer information material and as evidence in stakeholder feedback.  Children with cancer were supported by parents in the interview process, or where children were too young, parents were interviewed on their behalf. | tertiary oncology units and cancer interim homes across South Africa    The purpose of the thematic analysis was to identify and describe grassroots cancer-related challenges and thereby contribute to what is known about the experience of coping with cancer in the SA context. | The photo-narratives were then subjected to inductive thematic coding [12] using 3 coders to promote convergence and inter-coder reliability [13]. The thematic analysis coded the raw photo-narratives into categories of similar reference and meaning, thereby creating 9 themes of cancer challenges and contributing to the objectives of the study. |
| Edwards, L.B.,    2018    Evidence-Based Feedback About Emotional Cancer Challenges Experienced in South Africa: A Qualitative Analysis of 316 Photovoice Interviews    Global Public Health | Convenience sampling was applied to referrals from interim care homes and oncology units in 9  cities in South Africa over a 20-month period. | Qualitative  A semi-structured questionnaire facilitated the interview process, which asked two open-ended research questions and one open-ended interview feedback question. Face-to-face interviews facilitated storytelling, discussion and the documenting of narratives. Participants took a photograph that supported the meaning of their story. Unlike more standard photovoice methods where photographs are used to elicit discussion (Chapman, Wu, & Zhu, 2016), the study’s research questions elicited the discussion and the participants chose photo-images to support, illustrate or emphasise their documented message or story. | South Africa  9 cities    In/outpatient    This research explores grassroots photovoice stories of cancer challenges in the South African context, and this paper focuses on the emotional challenges theme | thematically analysed from a phenomenological perspective |
| Georgievski, G.,    2018    Through Our Eyes: A Photovoice Intervention for Adolescents on Active Cancer Treatment    Journal of Psychosocial Oncology | Recruitment initially involved clinical patient database searches to identify adolescents, aged 13–18, who were on active cancer treatment or had completed treatment within the previous three months. | Qualitative    Weekly 90-minute sessions took place over seven weeks. Besides photography, participants were given the option of creating drawings. The first week was used to review group goals, deliver camera training, and establish a schedule. For the proceeding weeks, participants collaboratively selected a theme for which they would take photos using point-andshoot digital cameras. These themes included strengths, struggles, true friends, fears, and hopes. Participants were asked to select three to four pictures to present each week printed at the start of each session. During each session, participants presented their photos and discussed how they related to the theme of that week. The last session focused on reviewing participants’ journeys through the group process – reflecting on the impacts felt, lessons learned, and relationships fostered. Participants also selected the photos that they felt were the most impactful and representative of their experiences for a gallery exhibition. | Toronto, Ontario, Canada    Urban    Outpatient(because they were contacted by phone?)    To explore the psychosocial needs of adolescents undergoing active cancer treatment using Photovoice as an intervention. | narrative content analysis |
| Hammond, C.,    2016    Cycles of Silence: First Nations Women Overcoming Social and Historical Barriers in Supportive Cancer Care    Psycho-Oncology | Not specified | Qualitative  Once we have permission, data collection occurs over 4 phases: (1) an  initial sharing session, (2) arts‐based activities, (3) follow‐up interviews, and (4) a second sharing session. The initial sharing session includes cancer survivors and their caregivers, who are invited to share their stories and to talk about their major challenges, sources of support, and understandings of healing. Secondly, only survivors are invited to engage in optional arts‐based activities to reflect on and facilitate their storytelling; in particular, they are given cameras (if needed) and journals and invited to use them to communicate any significant aspect of their experiences with cancer. They are also offered follow‐up individual interviews to discuss the content of their photographs and journals, including questions about the meaning and process of engaging in creative activities. In the final phase, all participants (survivors and caregivers) come together in a second sharing session to discuss the main messaging or themes of people's stories and who should be targeted for dissemination of the project findings. All interview and sharing session data were audio‐recorded and transcribed | First Nations communities across British Columbia, Ontario, and Quebec, Canada    Rural    Outpatient    To develop and disseminate knowledge of First Nations women's experiences with cancer survivorship, either as a cancer survivor, a professional caregiver to someone with cancer (eg, nurse), or informal (family) caregiver. | we ask participants to interpret and thematize their own data, as is typical in a community‐based research project. Their analyses are recorded as part  of the interviews. All data (photos, journals, and transcripts) are further analyzed by 2 team members using standard qualitative techniques (line‐by‐line coding, cross‐case comparisons, and hierarchical arrangement of themes based on presence in the data) to produce an integrated list of themes and subthemes.  The team reviewed the organization of themes and examples for fidelity, coherence, and representation. We have also taken the summary to community champions and select participants (3‐4 per site) who expressed interest in contributing to the process to review and confirm the path of our analyses. |
| Jellema, P.    2018    At Home in the Hospital and Hospitalised at Home: Exploring Experiences of Cancer Care Environments    Breaking Down Barriers | participants who met predetermined selection criteria were approached through convenience sampling | Qualitative  After initial interview, the participants were asked to visually document their experiences of the CCE during (one of) their next appointments. To take photos, they were offered the choice of borrowing a device or using their own. A selection of the photos was emailed to the first author in preparation of a follow-up interview. This process was repeated two more times with Helen and Lisa.. | Leuven, Belgium    Urban    outpatient    To identify common  threads in how the CCE is experienced by cancer patients and spatial aspects that  play a role in that experience. | Participants agreed to be interviewed at their home. For various reasons, Walter was not able to take photographs. Our findings are based on an in-depth study of Lisa and Helen’s material supplemented with the analysis of Walter’s interview material. The analysis of interview transcriptions was done following the guidelines of the QUAGOL method based on the constant comparative method of grounded theory |
| Jellema, P.    2020    The Roles of Cancer Care Facilities in Users' Well-being    Building Research & Information | Five patients, five adult relatives, and five care professionals. The patients were undergoing or had recently undergone cancer treatments. The decisive criterion was that during their participation in the study, they continued to have appointments. To form the triad each patient suggested a relative and a care professional involved in their cancer care.  Because the intention was to provide insight into a variety of cancer care facilities, sampling was guided by the aim to involve patients in contrasting spatial situations rather than by the aim to reach saturation. Male and female participants were recruited, from different age groups, being treated in academic or general hospitals, referring to their treatment as taking place in one or multiple locations. A sequential approach allowed patients to be selected in relation to the characteristics of those already participating. Three participants were found by consulting the authors’ personal and professional networks, two by approaching head nurses of oncology departments.. | To conduct this research we adopted a combined approach that included interviews, photographs and walking. The study was informed by a constructionist paradigm, assuming a pluralist position and an understanding  that knowledge is constructed in the interchanges between people, objects and activities. combine photovoice with walking interviews, in order to generate more place-specific data and examine the relationship between place and health. The first took place at the patient’s home or other preferred location, semi-structured with the topics covering: hospital visits, past treatment, current routines, and appointments. In preparation of the second interview, patients were asked to document their experience of their cancer care environment. They were offered a camera on loan but chose to use personal devices. Patients emailed 4–6 photos to the researcher before the second interview, during which a printed copy supported them to communicate their perspective. The third interview consisted of a walking interview in (and around) a cancer care facility. ‘sites-of-interest’ were identified prior to the walking interview. The researcher carried a handheld audio recorder. Participants were asked for no one else to be present during interview. The patient was asked to guide the researcher during the walking interview, putting the latter in contact with the environment while in dialogue with the former. | Leuven, Belgium  Urban (should be urban since all facilities are general hospitals, but not sure)  In/outpatient?    This article provides insight into the roles cancer care facilities play in the well-being of people affected by cancer. It offers not only insights for architects, but also handholds for care professionals to reconsider their position and seek opportunities to empathize with and positively influence the experience of patients and relatives | Qualitative analysis using NVivo, iterative process of coding, expert panel review    Following each interview field notes were made and recordings transcribed verbatim. Field notes, interview transcriptions, and photographs were collected and sorted in NVivo (Version 11). A qualitative analysis was conducted (based roughly on the QUAGOL guide, see Dierckx de Casterlé, Gastmans, Bryon, & Denier,  2012) through an iterative process of memo-writing, code and category development, and discussions with  the research team. Insights under development were presented to an expert panel to enhance the trustworthiness of the analysis in terms of confirmability (Guba & Lincoln, 1994). The panel included (ex-)cancer patients, a relative, a nurse, and a technical director of a care facility with a personal experience of cancer. Also, the decision to work with triads may be seen to enhance the quality of the inquiry, by adding elements of ‘ontological and educative authenticity: referring to how personal constructions of reality are added and built upon by others and how participants develop appreciation and understanding of each other’s constructions. |
| Lopez, E.D.S,    2005    Quality-of-Life Concerns of African American Breast Cancer Survivors Within Rural North Carolina: Blending the Techniques of Photovoice and Grounded Theory    Qualitative Health Research | Although each county has its own hospital, cancer patients and survivors often travel 35 to 100 miles to other counties for specialized care, support services, and cancer-related products for African Americans.  We used a purposive sampling strategy to recruit women who met the following criteria: (a) had completed their initial treatment, (b) were willing to take photographs about their survivorship, and (c) were open to sharing them with a small group of survivors. We also attempted to recruit women who varied on characteristics that could influence the survivorship experience (e.g., education, age, time since diagnosis, insurance coverage, and type of treatment). | One of two training sessions to learn about the project and what participation would entail. The women also practiced using their cameras by taking pictures of each other while they role-played using the acknowledgment form they would be required to use to gain written permission prior to taking a person’s photograph. the women developed photo “assignments” to help them to narrow in on the specific aspects of their survivorship they wanted to explore while alerting them to “everyday” experiences that they might have taken for granted. As a group, the women decided that their first photo assignment would be “Take at least 6 pictures that represent information I wish I would have had as a survivor.” The women took a month to complete each photo assignment, at which time we all reconvened for 3-hour photo discussion sessions. Our photo discussions typically began by our reviewing and discussing the themes that emerged from grounded theory analysis of previous sessions. We then conducted a show-and-tell activity, in which each participant presented her photographs and explained how they related to the photo assignment. The group then chose one or two photographs to discuss in depth, guided by the sixstep inductive questioning technique SHOWED: (a) What do you See in this photograph? (b) What is Happening in the photograph? (c) How does this relate to Our lives? (d) Why do these issues exist? (e) How can we become Empowered by our new social understanding? And (f) What can we Do to address these issues? At the completion of each photo discussion, as a group, the women decided on the next photography assignment to complete and scheduled the next photo discussion session. After five photo assignment–photo discussion sequences (which occurred over a 7-month period, because on two occasions, participants opted to continue discussing the same assignment for two sessions), we started hearing the same information repeated almost verbatim. | Eastern North Carolina, USA    Rural counties (populations less than 30,000, and are 45 to 62% African American)    Outpatient    we draw from our experiences conducting this project to describe how we blended the participatory action research method photovoice with the data collection and analysis techniques of grounded theory. We further present the findings that emerged when the participants, 13 African American breast cancer survivors, (a) recorded with their own eyes and explained in their own words how their QOL affects and is affected by their social  context and (b) developed a QOL conceptual framework to inform interventions to address the social forces that drive long-term concerns of rural African American  survivors. | the tapes transcribed verbatim to become the “raw data” for grounded theory analysis using the text analysis software, ATLAS/ti 4.2. our grounded theory analysis involved breaking down the data analytically by specific events, incidents, and actions and giving them conceptual labels. We then coded data within and across the different transcripts and speakers using the constant comparison method so that we could group related concepts into categories. We began developing our framework as conceptual relationships among the categories became evident and completed the theory when we were able to unify all categories around what we found to be the central analytical ideas represented in the data. we held a series of “final” findings meetings, during which the women reviewed the final themes and conceptual framework. We conducted these meetings until the women felt the findings credibly depicted what they wanted others to understand about their survivorship experiences. |
| Morrison, T.L.,    2014    Survivors’ Experiences of Return to Work Following Cancer: A Photovoice Study    Canadian Journal of Occupational Therapy | purposive sampling was used to solicit participation of 10 cancer survivors. An e-mail blast including a poster describing the study was distributed widely through professional and personal contacts. Posters were also displayed at various professional locations, including physiotherapy clinics. Interested participants contacted the first author directly. This conversation also permitted the verification that the participant met the inclusion criteria: (a) working at the time of cancer diagnosis, (b) 18 years of age or older, (c) able to provide consent, (d) able to communicate fluently in English, and (e) interested and felt able to take photographs reflective of their experiences and participate in two audio-recorded interviews. | Following consent, the following were completed: (a) a demographic survey, (b) an interview concerning the participant’s experience of cancer and health impacts as well as the participant’s worker role and related social considerations, (c) an orientation to photovoice methods using examples from another study, (d) training concerning the use of the digital camera should it have been loaned (participants had the option of using their own camera), (e) a review of ethics/safety of photography and the use of a thirdparty release form for other identifiable individuals captured in photographs, and (f) instructions to take approximately 10  photographs that hold some personal meaning of work return/maintenance following cancer prior to a second interview. Ten photographs were suggested to ensure that data remained manageable. Participants produced a range of 1 to 12 photographs. The second interview occurred 1 month later, during which the participants discussed the intended meaning of each photograph. | Ottawa, Ontario, Canada    Urban??    Outpatient??    this current study  endeavours to elucidate, in a provocative manner, the lived experiences of cancer survivors related to both their work return and maintenance | During the second interview, participants selected, contextualized, and codified their photographs using the following commonly used, root-cause questioning identified by the mnemonic SHOWED. A layered approach to data analysis was undertaken composed of preview, review, cross-photo comparison, and theorizing. The generated photographs were inserted into the second transcript for each participant, allowing the participant’s own words and attributed meaning to be closely tied to each photograph. Using NVivo 10, photographs and highlighted text were coded into nodes representing similar or repeating ideas (e.g., work’s representation of normalcy). Some photographs and text were coded to more than one node reflective of the number of ideas presented. Related nodes were grouped together to create preliminary themes |
| Morrison, T.L.,    2015    Comparing Men’s and Women’s Experiences of Work After Cancer: A Photovoice Study    Supportive Care in Cancer | Same as above    Additional efforts were necessary to recruit men including: disseminating information through cancer support agencies and local trade unions as well as presenting at cancer support groups involving men. Eight months of recruitment efforts were necessary to enroll 10 male participants. | same as above | Ottawa, Ontario, Canada    Urban??    Outpatient??    The findings of the current manuscript emphasize the findings stemming from the men, presented in contrast to findings derived from the women. | Same as above |
| Morrison, T. L.    2015    Cancer Survivors’ Concealment or Disclosure of Diagnosis: Implications for Return to Work    Work | Snowball sampling was used to solicit participation of cancer survivors who had been working at the time of diagnosis. Recruitment same as above. | same as above | Ottawa, Ontario, Canada    Urban??    Outpatient??    The purpose of this article is to describe the variable approaches used by participants to a recent study exploring the experiences of cancer survivors’ work return and maintenance. | Same as above |
| Mosavel, M.,    2010    Photovoice A Needs Assessment of African American Cancer Survivors    Journal of Psychosocial Oncology | Cancer survivors and caregivers were recruited primarily by word of mouth. However, an instrumental part of the recruitment for participants was done with the assistance of the community partner agency, Northeast Ohio Neighborhood Health Services (NEON). Potential participants had to either be cancer patients at some point or have cared for someone with cancer. | Four sessions were held: two of the sessions provided participants with training to take the pictures, and the other two were used to discuss the  pictures that were taken. Cancer survivors and caregivers were combined in a group, and there was an average of 10 in each group. Each session lasted approximately 3 hours, and refreshments  were provided. We primarily reviewed the ethics of picture taking, the importance of obtaining consent to photograph people, types of pictures to take, and how to capture their thoughts with a picture. . In the first session, the two groups also identified the major categories of needs for cancer survivors. In the next planning session we reviewed and addressed the challenges of taking their first pictures and did several role-plays or scenarios as well as planning for picture taking. The photos were given to the participants at the beginning of the meeting, and they were asked to categorize their photos, if possible, according to the theme sheet developed in the first meeting. The rest of the meeting was dedicated to discussing the pictures. | Cleveland, Ohio  USA    Urban  neighborhoods, of which 90% or more of the residents are African American.    Outpatient??    The goal of this research was to understand the barriers and challenges that African American cancer survivors experience after being diagnosed with cancer. | Three coders reviewed the transcripts to further distill the already defined categories and to explore if any new themes emerged. All three coders were cancer survivors. Extensive training was provided.  The data analysis group met five different times, and each meeting lasted approximately 3 hours. However, not all of the meeting time was entirely devoted to data analysis. The meetings continued with the coders sharing their impressions, categories, and notes from the transcripts. |
| O’Callaghan, N.    2024    Meaning of Nutrition for Cancer Survivors: A Photovoice Study    BMJ Nutrition, Prevention and Health | Inclusion criteria included cancer survivors (aged ≥18 years) who had completed active cancer treatment (≥6 months) and were living across Ireland. Participants had to own a smartphone.  For recruitment, the study was circulated on social media and throughout cancer networks across Ireland. Of the eight participants who responded, five responded from a more extensive quantitative study,  while three responded to online recruitment calls such  as e-newsletters from associated patient groups or social media platforms. | The photovoice project was conducted in four distinct phases, occurring sequentially over 8weeks in autumn 2021, and conducted online.  Introductory 1-hour workshops: overview of the project, Photovoice’s ‘four fs’ (frame, focus, follow through and flash) to taking better photographs, Exercises in visual literacy and photo analysis, ethical issues, Participants were then given a photography assignment titled ‘The meaning of nutrition for Irish cancer survivors’ and asked to take a minimum of five shots each using their smartphone during the following 2weeks  Individual interviews: 30-60min, participants were asked to share all photographs that they captured. This interview provided an opportunity with the facilitator to caption their selected pictures for the final workshop 2weeks later. Where required, the ‘SHOWED’ mnemonic, was used to discuss and caption each photograph  Final group workshop: Each participant introduced themselves to the group, discussed their cancer diagnosis and previous treatments received and explained their current perspective on nutrition, then presented their photos and captions to the broader group (20 min each). A miniexhibition of all photographs on a virtual platform. The group discuss the pictures collectively and then clustered the photographs into similar groupings using colour coding. participants discuss and identify potential themes represented by these clusters until the cohort had agreed  on the selected photographs. The group discussed theme names. Shortlisted images and captions were circulated by email post-workshop to ensure they had  been accurately captured. | Ireland    Urban/Rural???    Outpatient???    Thus, this study aims to use a visual methodology called photovoice to understand the significance of nutrition for Irish cancer survivors | In the final group workshop, participants acted as co-researchers to inductively develop six analytical themes representing the meaning of nutrition for them. These were then deductively applied by the facilitator to the rest of the data (eg, interviews and photos). This is a grounded practice known as ‘live coding’, aiming to maximise the researcher’s interaction with the data and ensure analytical rigour. It involves manual coding while simultaneously listening to the audio-recording of the workshop. Braun and Clarke’s six-step thematic analysis was conducted to determine if any additional themes arose during the discussions and Microsoft Excel was used for data organisation and mapping. No additional  themes were found in this process. |
| Pailler, M.E.,    2020    Reaching Adolescent and Young Adult Cancer Patients Through Social Media: Impact of the Photographs of Meaning Program    Journal of Adolescent and Young Adult Oncology | Eligibility criteria  included AYA cancer patients and survivors who were between 15 and 34 years of age at the time of diagnosis, diagnosed within 3 years, English speaking and who had received  treatment for their disease (e.g., chemotherapy, radiation,  surgery).    participants were excluded if they did not possess a smart phone, tablet, or computer with camera and Wi-Fi capability,  or a valid email address. Patients with significant cognitive  impairments, dementia, or untreated severe and persistent  mental illness were also excluded from participation. | Mixed Method Exploratory Cohort Study  Single-group intervention study?  10w Intervention via a mobile application PixStori, pre-training about setting up user profile and make Pixstories, at least two photos posted per week according to the weekly themes (introduced via email or text messages). Audio or text narration to describe the posted photos. Pts can view, like, and comment on each other’s posts.  Repeated-measures ANOVA (depression, quality of life, spiritual well-being), thematic analysis | Buffalo, New York, USA    Urban/Rural not specified    Outpatient not specified    To assess the feasibility and preliminary efficacy of the Photographs of Meaning Program (POM-AYA) for AYA cancer patients and survivors. | Self-administered questionnaire (baseline, 10w, 2mo after intervention)    Post-intervention phone interview    Dependent Variables:  Changes in depressive symptoms (measured using Beck Depression Inventory-II)  Changes in quality of life (QoL) (measured using PedsQL-YA)  Changes in spiritual well-being (measured using FACIT-Sp)  Participant satisfaction: satisfaction survey and semi-structured interview about the most helpful aspects of the intervention and aspects they would have changed or added |
| Park, J.S.,    2020    Photovoice-Based Assessment of Weight Management Experiences of Breast Cancer Patients Treated with Tamoxifen    International Journal of Environmental Research and Public Health | The participants of this study were nine breast cancer patients who were treated with tamoxifen at Kosin University Hospital and Saegyero Hospital in Busan Metropolitan City, Korea.  Specific criteria for selecting participants for the study were as follows: (1) those who had undergone surgery or chemotherapy or radiation therapy after diagnosis of breast cancer, (2) those who had been administered tamoxifen hormone medication for hormone therapy, (3) persons who, at the time of the study, did not have a recurrence or metastasis of breast cancer, (4) those who comprehended the purpose and method of research and who agreed to participate, (5) and those who were not administered psychiatric medications | Education: introduced the purpose and the content of the study as well as the method and the process of photovoice,  educated pt on how to write their own opinions on photography and photos, handouts and detailed information regarding photography, participants, methods, research methods, processes, and research ethics was provided, four themes of photography: (1) What does weight management represent for me? (2) What helps me manage my weight in my life? (3) What comes in the way of weight management in my life? (4) What do I need to do in my life to control my weight?  Photos were taken and submitted during 6–19 June 2019, for about two weeks, the participants were then asked to select three photos for each subject followed by an explanation.  The machines used by participants  in this study for photography were cell phones | Busan, South Korea    Urban    Outpatient    Therefore, this study intends to provide basic data to help develop a weight management strategy for breast cancer patients through in-depth analyses of their experiences of weight management using photovoice after receiving tamoxifen in Korea. | Primary data analysis: The researchers analyzed the photos received and participants’ opinions about these photos to derive the key themes.  Group discussion by subject: to confirm whether the researchers analyzed the photos and the interpretation of the photos accurately. The discussion was conducted with the SHOWeD.  Group discussion by topic: Participants discussed the submitted photos in two groups. The group discussion by topic required participants to select one of the pictures they had submitted that they thought was most representative of their thoughts on each topic. This was followed by comprehensive discussion.  Comprehensive discussion: participants assign a title for the photos selected and then pin them all on a large blackboard. This allowed participants to look at the photos on display together and to group and categorize the relevant factors by topic. On viewing the pictures divided according to topics, participants named each category of factors related to weight management among breast cancer patients until they arrived at a consensus.  Secondary data analysis by researchers |
| Poudrier, J.    2009    We’ve Fallen into the Cracks: Aboriginal Women’s Experiences with Breast Cancer Through Photovoice    Nursing Inquiry | Aboriginal organizations, universities, a cancer centre and Breast Cancer Action Saskatchewan distributed information about the research. A Me´tis elder, who wishes to remain anonymous, also provided guidance during the study design and wrote a newspaper article in a local  Aboriginal publication in support of the study  Recruitment criteria were (i) 19 years of age or older, (ii) completed active breast cancer treatment at least 6 months before our study, (iii) be able to provide informed consent, (iv) reside in Saskatchewan, and (v) identify as Aboriginal. | At the first meeting, participants were asked to share their story of having had cancer and these stories were digitally audiotaped. Participants then borrowed the digital cameras for several weeks. The women were asked to take any number of pictures their experience of breast cancer meant to them, with the suggestion that 12–15 photographs would be sufficient  During the second digitally recorded interview, the women were asked to discuss the photos and to provide a description of what the photo was meant to convey. They were also asked to select and discuss several photos that they felt were especially meaningful to them  In a following 1-day event, the women came together in a morning sharing circle to share their photographs and stories in a safe and comfortable environment. In the afternoon, key stakeholders from advocacy groups were invited to join us in a workshop to  discuss forming new networks this new group of women. | Saskatchewan, Canada  Three of the women lived in urban areas in Saskatchewan while the remainder lived in rural towns or in First Nations communities    Outpatient  Our main objective was to explore and to begin to make visible Aboriginal women’s experiences with breast cancer using the qualitative research technique, photovoice. | The participating women provided the initial and most important interpretations of the photos and discussed key themes extensively during the second interviews. The women were asked to describe the meaning of the photographs and to choose those which were most significant to them. Extensive team discussions, as well as review of the transcripts and photos then guided the interpretive processes. Our theoretical framework, in the domains of feminist epistemology with a focus on visibility and power, provided filters through which we collectively interpreted and thematized the stories and photos. independent coding by the three researchers, second, with extensive team discussions, and finally, with the Aboriginal women in a sharing circle following the individual interviews. Finally, participants were asked to provide feedback on this research process. |
| Power 2022  “Surviving Discrimination by Pulling Together”: LGBTQI Cancer Patient and Carer Experiences of Minority Stress and Social Support | Participants were eligible for this study if they: (a) had been diagnosed with cancer, had undergone a medical intervention related to cancer risk or had cared for someone with cancer; (b) they or the person they cared for identified as LGBTQI, and (c) were at least 15 years old. Participants were recruited through cancer and LGBTQI community organizations, including the study partner organizations, social media (Facebook, Twitter, Instagram), cancer research databases (Register 4, ANZUP), cancer support groups and LGBTQI community events. Snowball sampling was also used | Survey, interview, Forty-five patients and ten carers completed an additional photovoice activity.  Photovoice involves participants taking photographs that visualise elements within an individual’s life pertinent to a particular phenomenon (62). Situated within an action-research model, photovoice methods facilitate involvement and empowerment of research participants and have been described as an innovative way of working with marginalised people, including LGBTQI communities (63, 64). Participants were invited to submit three to five photographs that represented their experiences with cancer, which were then discussed in a second interview. Written and visual instructions were provided to participants to aid in the photovoicep process. Participants used their devices (smartphones, digital cameras) to take photographs and electronically submit them to the research team. The photographs were used as the basis for subsequent discussions to understand more about participants’ experiences of cancer and often eliciting in-depth descriptions of specific events. Participants were asked questions such as “Could you please talk me through these photos and explain what they mean to you?” and “How does this photo capture your cancer experience?”. | Australia and other English-speaking countries such as the USA, UK, New Zealand, and Canada  The present analysis aimed to address this gap in the research literature by examining subjective experiences of minority stress and social support among LGBTQI people with cancer and their carers, drawing on the qualitative findings from the mixed method Out with Cancer study (27, 53–55). | Thematic analysis was used to analyse the open-ended survey, interview and photovoice data, as an appropriate method to capture richness across multiple data types |
| Wong, S.S.    2019    Using Photography to Explore Psychological Distress in Patients with Pancreatic Cancer and Their Caregivers: A Qualitative Study    Supportive Care in Cancer | Inclusion criteria encompassed male and female patients diagnosed with primary pancreatic ductal adenocarcinoma in the past five years and their self-identified primary caregiver. Patient refusal did not exclude caregiver participation and caregiver refusal did not exclude patient participation. Exclusion criteria encompassed age less than 18 years, insufficient English, and poor performance on the Mini-Mental State Examination (score ≤ 23).  Medical records were screened between August 2014 and June 2016 for eligibility. Eligible participants interested in research opportunities were approached at their medical appointment. | All participants received a training orientation to Photovoice methods, tips on digital camera use, and ethics of photography. Two biweekly 90 minute group discussions were held for each participant on the focus question: What do you want others to know about what it is like living with pancreatic cancer or caregiving for someone with pancreatic cancer? Topics were self-generated by participants. Discussions were held in small groups of 2–3 people. Participants were instructed to bring three photos corresponding to their selected topic, including a title and brief narrative about the photograph. At the first group discussion, we asked two main questions: 1. Why did you take this picture and how does it relate to the topic? 2. How are the other group members’ experience similar or different? The SHOWED technique [15] was also used to further contextualize the photographs. At the end of the first group discussion, we asked participants to identify a second topic for their next photo assignment and repeated the same procedures listed above for the second group discussion. All sessions were audio-recorded for analyses. | Winston-Salem, North Carolina, USA    Urban    Outpatient    The purpose of this study is to gain a richer understanding of the factors associated with psychological distress from patient and caregiver perspectives | Group discussions were analyzed using thematic analysis. Key phrases were highlighted and coded into nodes representing similar ideas; text that reflected multiple ideas were coded to more than one node. Related nodes were grouped together to create preliminary themes, which were then reviewed with the participants to ensure accuracy. Ongoing data analysis and enrollment of participants continued until theme saturation was reached for patient and caregiver groups. Theme saturation was determined when no new information emerged from transcripts. Analysis team meetings from multiple disciplines (psychology, oncology, sociology) were then held to review and refine categorization of themes. Pseudonyms were assigned to conceal identity of the participants. |
| Wong, C.L,    2024    Understanding the Experience of Cancer Survivorship Among Pediatric and Adolescent Cancer Survivors and Their Parents Through Camera Lenses: A Photovoice Study    Psycho-Oncology | Participants were recruited from the Long-term Follow-up Clinic at one of the largest public hospitals in Hong Kong. Eligible survivors of pediatric cancer and their parents were invited to participate in this study. The inclusion criteria were as follows: (1) Hong Kong Chinese pediatric cancer survivors; (2) aged between 9 and 18 years at the time of study participation. Both survivors and parents were required to have proficiency in speaking Mandarin/Cantonese and reading Chinese and could use digital cameras or smartphones with a camera. The refusal of participation by the survivors did not automatically exclude the parents from participating, and vice versa. However, survivors and parents with identified cognitive and learning problems were excluded. | Participants were asked to use their cameras to take 10 photos reflecting the experience of cancer survivorship in the coming 4 weeks, and wrote a narrative on each photo by answering the following prompts:  What does cancer mean to you?  How did you feel during cancer survivorship?  The principal investigator arranged individual interviews with both the survivors of pediatric cancer and their parents after they shared 10 photos. The interview followed the PHOTO technique by asking participants to describe the photos using five standard questions: (1) Please describe your photo; (2) What is happening in this photo; (3) Why did you take this photo; (4) What does this image tell us? and (5) How can this image provide an opportunity to improve cancer survivorship? Each participant was given a photo album containing all the photos taken by them as an incentive after completing this study. | Hong Kong, China    Urban    Outpatient???    The present study aimed to understand the experiences of cancer survivorship among pediatric and adolescent cancer survivors and their parents by employing a photovoice approach. | Thematic analysis was employed as a methodological approach to analyze the photos, narratives, and interviews. Initial codes were systematically generated by identifying interesting features throughout the entire dataset. These codes were then organized into potential themes, and the relevance of these themes to the coded extracts and the entire dataset was examined, resulting in the generation of a thematic “map” of the analysis. member checking was performed, allowing participants to review and provide feedback on the interpretation of their data. |
| Yi, J.,    2010    Self-Portraits of Families with Young Adult Cancer Survivors: Using Photovoice    Journal of Psychosocial Oncology | PADRES program staff identified six YACS who were diagnosed with and treated for a pediatric malignancy prior to age 21, were off treatment for more than 5 years, and were between ages 18 and 39 at the time of study. Six YACS were recruited and asked to bring a family member | A total of seven sessions were held every 20 weeks on Saturday mornings at Children’s Hospital Los Angeles. The first meeting consisted of an orientation to the project. Staff distributed digital cameras, instructed them in their use, and then directed them to take a  self-portrait. At the end of Day 1, participants are asked to take photos over the next 2 weeks to capture “the impact of cancer on your family.” All subsequent topics for future photographs were generated by the participants themselves through group discussions during each of the subsequent sessions. Each of the next five sessions lasted 2 hours. Upon arrival, project staff conducted a 10-minute individual interview to select one or two photos. Participants provided captions for these photos. The interviewers asked the following questions to stimulate discussion of the photographs: What are you seeing in this picture?; What is happening in this picture?; How do you feel about this situation?; and Why does this happen? (Wallerstein, 1987). After the individual interviews, participants were divided into a YACS group and a family member group and participated in an hour long group discussion. At the end of the hour, each group reached consensus on a theme for the next photo-documentation period | Los Angeles, California, USA    Urban    Outpatient    The specific aim of the study is to report on  themes that YACS and family members identified as relevant to their experience of cancer survivorship. | We used the themes generated by participants as an organizing framework or template. Because the YACS group and the family group developed separate lists of themes, we analyzed the transcripts of the two groups separately. Also, because the participants were supposed to take photographs and have discussions about the group theme on each day, we analyzed the transcripts  from each day separately. |
| Yi, J.,    2016    The Experiences of Korean Young Adult Survivors of Childhood Cancer: A Photovoice Study    Qualitative Health Research | We recruited seven young adult childhood cancer survivors diagnosed from birth to 19 years of age and currently between 19 and 29 years old who had completed cancer treatment. we purposefully recruited those interested in sharing their cancer experiences and advocating for their community of cancer survivors. We excluded those currently in cancer Treatment. The announcements were posted on cancer survivor advocacy foundation and support group websites between September 2013 and October 2013. | The study was conducted on five consecutive Saturday mornings. Each session took about 3 hours. The first session consisted of an orientation and then a group discussion on cancer survivorship. In the orientation, the photo-taking process was discussed and the participants were instructed to take 10 to 15 pictures with their cell phones or digital cameras that they felt captured the weekly topic for each week’s session as selected by the group (but not to take photos of individuals due to privacy concerns). At the end of the initial and four subsequent sessions, the group decided on the topic that they would take photos about in the coming week before the next session. Between sessions, a member of the research team, contacted the participants to check on their progress. In subsequent sessions, each participant was asked to select two or three key photos, which were projected onscreen in a group discussion (1.5–2 hours long). the participants presented and shared their photographs with childhood cancer survivors from other Asian countries at the 7^th^ ICCCPO Asia Meeting in April 2014. | Seoul, South Korea  Urban  Outpatient  This is the first qualitative study using Photovoice to investigate the impact of childhood cancer experience on young adult long-term survivors in Korea. | Data analysis was simultaneously conducted while the weekly sessions occurred. The weekly themes determined by the participants became major themes of the study findings. The research team members open-coded each session’s transcript to find subthemes under the major theme by repeatedly reading the transcripts. Field notes were also used to capture the subthemes that were important, but did not otherwise stand out in the written  transcripts. As previously noted, the participants selected each session’s theme, and the constant comparative method (Strauss & Corbin, 1990) was used when analyzing session subthemes. |

**Appendix A6: Photovoice Study Data: Results n=26 studies**

| **First Author**  **Year** | **Participants**  **No. and Demographics** | **Results (Themes, Subthemes, Descriptions, Sample Quotes)** | **Limitations** | **Recommendations** |
| --- | --- | --- | --- | --- |
| Bates, M.J.  2018 | N=13    Total: 13 participants  6 <40 years, 7 > 40 years; Gender Breakdown: 6 male, 7 female  Role Breakdown: 6 cancer patients, 7 family caregivers  Religious Affiliation: Majority Christian (10), one Muslim (1), other (1)  Cancer Types:  Kaposi’s Sarcoma (5 cases, all HIV+)  Brain, cervical, and thyroid cancer (3 cases, one HIV+)  Treatment: average 42 months receiving palliative care (9-120 months) | 1. Things That Make Us Happy  The ability to work or perform daily chores (such as walking, cooking and cleaning) was considered important for what was ‘a good day’; Seeing improvement in the patient brought happiness.  Example Quotes:  *“We should be able to walk like that granny when we have woken up better, walking so that the legs should be strong.”*  “Here this lady is washing her clothes. She woke up strong this day that she could wash on her own”.  *“. . .even though some of us are sick from this disease we are working because of the medicine that we are receiving from Tiyanjane, they give us medicine but seeing that we are able to walk and we are working knowing that our family should be ok because of the medicine that we are given”*  2. Courage Givers  Family caregivers, neighbors, children, and religious groups provided emotional strength.  Prayers and religious gatherings played an important role in supporting patients.  Example Quotes:  *“The people who happen to encourage us on some other things for example maybe at home. . .being free with you by talking to you that you are not supposed to be worried this is not the end but the beginning . . .those are the category of people assisting in your everyday life like the everyday food and activities.”*  *“I can see some carrying bibles. . .It’s not that your life has come to an end just because you*  *have been diagnosed with cancer so those people comes and happen to give you courage*  *with the words from the bible”.*   s, “when you go there they encourage you and gives you enough courage that . . . although I have been diagnosed with cancer it doesn’t mean that I will die today, no I will be alive as long as I follow what the Tiyanjane people are saying”.  3. Discrimination  Discrimination was described as being due to cancer patients being thought of as being infectious and/or ‘prematurely dead’  Example Quotes:  “My wife left me when I first got sick of cancer, but my child is happy now”  *“There are neighbors who insult cancer patients, saying, ‘That one will die soon; there will be a funeral at that house, he should just*  *die’. And some spit when they see me failing to walk, so (I) am asking that these neighbours should not speak these things.’”*  *“If they are discriminating you on some other things. . .while they have eaten good food but giving you things that ‘just give him a little something to eat’ then the disease continues.”*  4. Cancer as an Illness  Co-researchers highlighted the worries, shock and concerns caused to patients and families when they hear the news that they have a diagnosis of cancer  *“I feel messages concerning cancer are not widespread. . .in the villages it is very difficult to reach out because some people have no radios. So if the country or the government can take part by using different ways to disseminate the message of cancer so (we) should know this problem earlier before it gets worse”,*  5. The help that you get from the hospital  Palliative care services (Tiyanjane) were reported to provide counselling and medication which assisted patients to return to work.  Patients and family caregivers were supported to stay together and care for one another by reducing discrimination  Transport to clinics was a major barrier, leading to delays in care.  Example Quotes:  “I now work properly on my business and all my household chores after receiving medication from Tiyanjane”.  *“The children also getting closer to the patient. . .not being far away because of the counsel from Tiyanjane. Here, without Tiyanjane, our guardians taking care (for us patients) would have been far from us”*  *““In my case I had to travel up and down for more than three weeks in order to be assisted. I did that because my guardian assisted”.”* | Small sample size, limiting generalizability.  No info was given about data saturation    Findings specific to urban/peri-urban Malawi, may not apply to rural settings.    Patients who can take photos are relatively healthy    Long-term relationships between palliative care staff and co-researchers facilitated accountability and acceptability of the process, though is likely to have introduced some bias. | Co-researchers  (patients and family caregivers) played a central role in the analysis approach    Balancing safety, trust and study acceptability for co-researchers whilst eliminating bias was challenging.    They identified a role for themselves in carrying cancer messages to their communities. This reflects the potential for ‘enhanced community engagement in action and advocacy’ discussed in previous Photovoice projects  and would be an important area for future work.    Improve access to palliative care at local health centers to reduce travel burdens and to reduce stigma and discrimination through counselling of patients and families and by modelling close contact in clinical settings and through visiting patients at home.    Train healthcare workers on culturally competent care for patients facing discrimination.    Develop financial aid programs for low-income families caring for cancer patients.    Enhance support groups for caregivers and patients to reduce social isolation. |
| Bood, Z.M. | N=12    Gender Breakdown: 11 female, 1 male  Age Range: 23–35 years old (Mean = 31, SD=3.6)  Cancer Types:  Lymphoma, breast, ovarian, brain, testicular cancer  Treatment Status:  6 receiving curative treatment  5 receiving palliative treatment  1 did not specify | Concerns for the Future  Fear and uncertainty about relationships, education, work, finances, and housing.  Her concerns surrounding work was also the topic of one of her photos. On the photograph, she sits at a white table with the mask of the radiation therapy on her face (Fig. 2b). The photo is accompanied by the caption: “At job interviews they will come up with excuses for not hiring me. I already want to shout it out: Look at me, look at my skills, and do not look for traces of my disease.”  Struggles with (Re)defining Identity  Perception of others: A 25-year-old female was afraid others thought she was lazy. A photo from the photovoice shows a building with scaffolds in opaque covering (Fig. 4a) — a metaphor for her working on her personal development, but people not being able to see this from the outside. A second photo of this participant pictures herself with a hairnet on, her back to the camera, and holding herself (Fig. 4b), accompanied by the following text: “You can’t see anything from the outside. That’s why they often cannot believe it, cannot comprehend it. […] Sometimes I fantasize about being bald. Would people then realize that this is real? (But thank god I still have my hair).”  Changing appearance and self-image: The other AYA had a photo made in which she is sitting in a chair with just a tank top and big boots on, showing part of her body (Fig. 5b). The caption of the photo shows that she had a hard time accepting her changed body: “Hair loss, amputation, weight gain; I am trying to accept myself and I know I can be proud, but it often does not feel like that. I worked hard to love my body, to form it to my image of a hot woman. Now I lost all footing.”  Sexual orientation  One of the participants (32-year-old female) had a photo made to express her struggles with experimenting with her sexual orientation during the cancer treatment (Fig. 5c). The photo shows ribbons in the colors of the rainbow that are tangled, and is accompanied by the caption: “I was ready for it, creating an account on Tinder, to figure out if I like women or men, maybe something in between. Now it’s way too complicated. Do I use an old photo or a photo with me without hair? The fear of rejection paralyzes me.”    Comparison of Rich Pictures vs. Photovoice  Both methods captured similar themes, but some different subthemes emerged: For instance, while in both RPs and photos the effects of the cancer and treatment on the body and body-image were mentioned, the uncertainty surrounding being able to become pregnant was only depicted in the RPs. Furthermore, the difficulty of dating was also depicted in both visual tools, but the impact of the cancer on experimenting with one’s sexual orientation was only visualized in the photovoice. We will expand on the two main themes with examples from the RPs and photos. | primarily female participants.    Selection bias: Participants were self-selected and interested in creative expression.  Photovoice and RPs were treated as two completely separate elements, while integrating them might have been even more beneficial for gaining insight into the experiences of the AYAs.    only one male participated in our study    Time and energy-intensive methods may exclude those with advanced illness or severe fatigue. | Expand visual storytelling interventions for AYAs to facilitate emotional processing and self-expression.    Improve psychosocial support services addressing fertility, body image, and career concerns.    Develop peer support programs for AYAs to combat social isolation.    Educate healthcare providers on the importance of visual tools in patient communication.    Integrate visual tools in AYA cancer care to help patients articulate experiences beyond verbal descriptions. |
| Capewell, C. | N=20    100% female, 100% white with breast cancer, various time since diagnosis | 1. Waiting  Slow time pass during the diagnostic and treatment process increased anxiety and emotional distress.  Time is distorted by the waiting, leading to heightened uncertainty about survival.  2. Fear/Shock  The sudden impact of diagnosis was described as devastating. Many women never expected they would get breast cancer. *“Wham, I wasn’t expecting that.”*  The diagnosis led to consideration of their mortality. Pat’s response was: “And the first thing out of my mouth was, ‘Am I going to die?’” This led to solidarity among participants as they acknowledged their shared experiences and emotions. They felt uncertainty about the future, leading to hesitation about any forward planning. Ivy said, “I wouldn’t book anything because I thought that I might not live to doit.”  3. Lack of Control  Feeling powerless in decision-making about treatment. Scheduling dictated their lives, “ ... it’s like being in limbo, because you’ve lost control. You’ve lost control, that’s what I felt. That really for twelve months I’d lost control.” In the women’s normal role, they  are directing their families and households, thus they felt their self-identity was challenged.    4. Communication and Practicalities  Inadequate communication from medical professionals increased distress. Helpful gestures from health workers made a big difference. Ivy identified that, “information is what gives you control.” Iris highlighted that, “The information in the booklets is sometimes  out of date and not always right.”    5. Body Image Changes  Hair loss, weight changes, and scars had lasting psychological effects. The women appreciated advice on appropriate wigs, makeup, and the use of scarves. Having the opportunity to share their experiences and their emotional impact gave them a sense of community.  *“I’d just wake up in the morning and the pillow would be full of hair. Or any gust of wind and your hair floats away”*    6. Specialist vs. Nonspecialist Centers  Specialist breast cancer centers provided better support and information.  *Specialist Breast Cancer nurses were particularly valued: “She would write things on the front of leaflets and make it*  *personal” (Faye). This contrasted with, “in the local hospital there is not a breast cancer ward, so you go onto a mixed ward. I got most upset because some nurses they thought I was in for a breast reduction” (Lucy).*  7. Ongoing Support Needs  Support groups and peer networks were invaluable for emotional well-being.  *“Talking to others who had experienced a diagnosis and treatment of breast cancer was valued, there was a lady who used to ring me up. She had had breast cancer and it was like peer support ... and she had had some training on how to talk to people that was really amazing and so supportive (Ivy)”* | Small sample size (n=20), no mention of data saturation, limited diversity (all white women). Findings may not apply to different racial, socioeconomic, or cultural groups.    No direct involvement of participants in data analysis. | The concerns raised by Ocloo and Matthews (4) as to how  patients can be actively involved in the implementation of  In dissemination activities with cancer support organizations and specialist centers, as the material produced for the exhibition was both visual and verbal, provided access to the participants expressing themselves and giving insight to professionals.    Improve communication training for oncology staff and general practitioners.    Increase access to breast cancer specialists for personalized support.    Provide better patient education on what to expect, particularly regarding post-surgical recovery.    Two sisters who participated had been  diagnosed with breast cancer about the same time. They felt the research allowed them to share things not previously discussed or shared with each other. The opportunities to  participate in activities and conversations with others who had had the same diagnosis were seen to be valuable and  important. Expand self-help groups may provide effective psychological support both during and after the end of medical intervention.    Reduce wait times and improve scheduling systems to minimize anxiety. |
| Currin_McCulloch, J. | N=11    Age Range: 28-44 years old (Mean age: 31.36)  Diagnosis age: 20-37 (mean 27.45)  Gender Breakdown: 100% women  Race/Ethnicity:  100% non-Hispanic  9(81.82%) White  1(9.09%) Black or African American  1(9.09%) American Indian/Alaskan Native  Cancer Type: Not specified  Relationship Status:  5(45.45%) married  5(45.45%) single  1(9.09%) engaged  Education Level:  Some college 2 (18.18)  College graduate 3 (27.27)  Some graduate school 1 (9.09)  Completed graduate school 4 (37.37)  Doctorate degree (PhD) 1 (9.09) | 1. Group logistics: Enhancing Unstructured Connections  ways to enhance photo sharing time: (1) “maybe have another 1/2 hour because it seemed rushed” (P3); (2) “have a set time for each member to talk so you can hear about everyone’s photos” (P8); and (3) “I did not like there was a cap on photo submissions; some respected it and some did not ... Having a timer would have been helpful” (P6).    2. Photo sharing: Representing a Feeling with a Photo  the ability to tell their cancer story through photos as a refreshing medium: “[this was an] interesting way to think about your journey by sharing a photo to represent a feeling” (P6). Another explained, “I noticed that I learned a new tool on how to share my story. . .Photo sharing is a great way to document a story through prompts” (P4). Lastly, a member explained, “I appreciated how it made me think in a nonverbal way. It helped me connect to feelings in a deeper manner and it allowed me to connect better.”    3. Virtual format: A Convenient Way to Talk with YAs Around the Country  members relished the chance to meet peers from across the country: “these options [to have support from around the county] is valuable” (P5). The COVID-19 pandemic and members’ compromised immune systems created barriers for their participation in social activities: The online platform reduced worries about “getting someone else or myself sick” (P1). As busy people with jobs and families, the Zoom format offered more time to be present within their lives and eliminated transportation barriers. Although the online platform offered many benefits, members felt that a downside of Zoom was not being able to see their peers in person and tehcnical challenges.    4. Session topics: Thinking About What I Want to Do with My Future  They enjoyed the first session as they were able to share several facets about themselves which set a good foundation for the rest of the sessions. Participants shared that the legacy session caused them to share fears about death, a topic not readily addressed within their intimate circles: “Hearing everyone’s experiences and legacy stood out because in a way it made me sad some [members] are not going to have a huge future that others might have. Their quantity of life may not be that much left. It stood out it, just made me sad” They longed for more informal sharing of both positive and negative happenings in their lives. Photo prompts limited their ability to talk about negative things, “you  don’t feel great or when things suck” (P5). Frustration with knowing each groups’ topic until one week ahead.  5. Connections of Curiosity  Photos created “connections of curiosity in seeking to understand people better and learn more about them” (P4). Meeting with other YAs provided a sense of normalcy and  universality in experiences as a YA survivor: “I was surprised by some people’s  stories because I had not heard that point of view before. [In the group] I did  not feel I was an ‘other.’ I feel othered because of cancer, because of my hair,  and having people say I lost my hair” (P8).The group was seen as “positive and  encouraging” (P3) and members viewed their peers “as a resource or support if needed” (P1). When they attended cancer support programs, they often felt different than other members: “I think, because I had only spoken with breast cancer patients, that it was neat to learn we have similarities because of our age, even though we have different cancers” (P5).  Midway through the group, members expressed an interest in connecting  outside of the group. Two members commented on how they would have liked to have more of a connection outside of the group, although another member shared that they were overcommitted, and additional communication would cause stress. | Small sample size (n=11), all female participants.    Limited racial and socioeconomic diversity.    Challenges with recruitment and engagement.  Study took place during COVID-19, which may have influenced participation and outcomes. | Provide more flexibility in sharing and discussion time during sessions.    Increase outreach efforts to diverse racial and socioeconomic groups.    Integrate Photovoice into broader YA cancer support programs. |
| Ebrahimpour, F. | N=20    Age Range: 6–12 years old  Gender Breakdown: Equal number of boys and girls  Cancer Types:  Acute Lymphoblastic Leukemia (ALL) 16 Ewing’s Sarcoma 1 Lymphoma 2  Abdominal malignancy 1 | 1. Emotional Connectedness with Nursing Staff  A nurse that not only provides care, but also plays with the child: “Mrs… gives me a sense of hope. Because sometimes she plays with me, for example, when she comes to my room, she looks at my dolls and toys, and we play dolls together, or sometimes we paint together.”(6-year-old girl with leukemia)  A nurse who uses friendly, instead of formal, interactions or who hears and understands: “There are nurses who are kind and listen to me. For example, when they inject the medicine, I tell them to inject it slowly, so I experience less pain. And they listen to me.”(7-year-old girl with leukemia)    2. The Playroom as a Means to Soften the Hospital Space  An opportunity to interact with friends and get rid of bored feelings: “The playroom makes me happy because I can see my friends, but it’s not open every day. I like to go there whenever I don’t need to receive medication.”  Opportunity to forget the disease for a while: “I feel happy in the playing room, and I forget my disease. ”  The play is a symbol of life: “The playroom is the only hope and joy for children. Without it, we will be dying.”    3. Presence of a Parent  Mother’s love seals of reassurance and hopeful words: “My mother gives me hope because she is so good and kind.” “I took a picture of my mother because my mother says, if you take your medication, you will get well soon, and you can go to school.”    4. Symbols of Recovery  Improving the physical condition “Whenever I compare my current status with the first month of my cancer diagnosis and hospitalization, I feel better. That time I couldn’t get out of bed, I had pain in my legs, and I couldn’t walk out of my room. But now I can walk. It gives me a sense of hope  that I’m getting better.”  Physician as a sign of recovery “This is my physician. I have a special feeling when I see her. I tell myself, she is my physician, and she will heal me.”  Spirituality and recovery “This is the praying room, here mothers pray for our health. It gives me a sense of hope that we will get better.”    5. A Touch of Nature in the Hospital Setting  Exposure to plants, flowers, or nature-themed murals made children feel more relaxed and hopeful.  “I took the photo of this flower painting on the wall because it smells good beautiful and gives me a sense of hope.”    6. Escaping the Hospital Cage  Many children saw the hospital as a prison and looked forward to discharge.  “I took a picture of myself because I’m happy. I’m discharged, and I want to go home.”  “I took a picture of my father because he takes me out of the ward and sometimes, he takes me to the yard because we can’t leave the ward. The guards don’t let us go out. But when my father comes, I can go out and have some fresh air.”  “I took a picture of the door because I’m leaving here. I don’t like the hospital. Every time I go out, it means that one of my hospitalizations’ turns is over, and that’s hopeful because one day, all my hospitalizations will be over.”  “Here I can find storybooks. My mother read me one of these books. I love storybooks.”  “I love pasta. They give me pasta here, but my mother’s pasta is tastier.” | Small sample size (n=20), all from a single hospital in Iran.    Focused mainly on ALL patients, limiting generalizability to other childhood cancers.    Cultural influence: Some parents preferred to hide cancer diagnosis from children, affecting responses.    the limitation associated with conducting lengthy interviews due to both their age and disease | Encourage positive nurse-child interactions to increase hope and reduce pain and discomfort    Integrate nature and art elements into ward design.    digital photos with phones is a simple creative intervention that children are likely already doing, and nurses can encourage to help bring hope to the process the experience of living with cancer |
| Edwards, L. B.    2017    A Descriptive Qualitative Study of Childhood Cancer Challenges in South Africa | N=68 58 patients and caregivers (parents, grandparents, siblings, childhood cancer patients), 10 pediatric oncology workers    Patients' Age Range: 8 months to 18 years  Types of Cancer Diagnosed:  Acute lymphoblastic leukemia (23) Kidney (9) Brain (8) Lymphoma (5)  Cancer of the eye (3)  Myoepithelial carcinoma (2)  Other cancers (Fanconi anemia, sarcoma, liver cancer, thyroid cancer, sinus cancer, rhabdomyosarcoma)  Geographic Representation:  77% of patient families lived more than 50 km away from the nearest tertiary oncology center | 1.Emotional Challenges 67/68  Parental and famiy distress relevant to employment, financial concerns, separation of families, fear and uncertainty about the childhood cancer patient’s health and concern for siblings left at home.  Children struggled with being concerned about family, missing school and friends, and feeling different from peers.  Positive coping factors: Family, peer, and community support, caring medical staff, and spiritual faith.  Example Quote:  *“I feel ill from being so upset all the time.” – Mother of an 8-year-old leukemia patient*    2.Information Challenges  Lack of public awareness about childhood cancer led to delays in treatment. Need for medical care information. Parents and guardians lack of information due to exclusion during medical care, lack of access to doctors, language barriers, and no translation services.  *“Understanding what is happening to your child is very important.” – Mother of a 9-year-old with brain cancer*    3.Physical and Treatment Challenges  Pain, headaches and seizures, chemotherapy side effects, disabilities, and trauma of relapse.  *“I hated having the seizures, it was horrible.” – 11-year-old girl with leukemia*    4.Powerlessness and Exclusion  Parents reported feeling powerless due to lack of communication, lack of information, and threat of cancer.  *“I was not allowed to stay in hospital with my grandchild... I did not know what was happening to her.”*    5.Challenges of Poor Healthcare Services  Misdiagnoses, not trust PCPs, fears about delay in diagnosis and treatment, and inadequate facilities.  Overcrowded hospitals, lack of facilities, treatment, medications, support, palliative care, disrespect for the dignity, and uncaring medical workers made care difficult.  *‘The clinic kept thinking it was worms or HIV and only after 3 months sent him to hospital where he was misdiagnosed with constipation, and eventually referred to the cancer hospital.’ (PvM283, Mother of 4-year-old son; acute lymphoblastic leukaemia)*    6.Financial Challenges  Loss of or threat to work and income; care costs; financial distress, shortages of food and life essentials, lack of assistance  *“I worry about what my family at home will eat because we used all our money for treatment.”*    7.Transportation Challenges  worry and a financial burden with travelling long distances; logistics of travel *“I was not allowed to stay with my child at the hospital so when he was transferred I met the transport and saw that he was on the floor of the ambulance on his knees, the nurse at the regional hospital was very angry to see my child transported in this way.”*    8.Stigma challenges  Some communities saw cancer as a curse, bewitchment, or punishment.  Fear of discrimination and isolation caused families to hide diagnoses.  *‘I was scared that my neighbours would think that I was cursed because my husband died and now my grandson has cancer.’*    9.Schooling Challenges  Many children missed school or dropped out due to treatment.  “My child failed grade one and has to redo it, but there is no schooling at the hospital, I am very worried about her education.” | Small sample size    Uneven geographic distribution of participants    Limited focus on palliative care issues | Improve public awareness and early detection of childhood cancer.    Challenges with a lack of community services, home care and palliative care    Implement better patient-centered care practices.    Expand financial and transport support for families.    Provide on-site education programs for hospitalized children. |
| Edwards 2017  316 | N=316 286 cancer patients and family members (called patient participants) and 30 health care participants    232 Patient participants used the public health system and 54 used private medical care. 138 Patient participants were male, 148 were female | 9 Themes of cancer challenges were identified.  92% of patient participants described emotional challenges such as acute and ongoing anxiety, fear about the future and death, distress of a cancer diagnosis, physical suffering, loss experiences, emotional support problems and concerns about family. Some participants emphasised positive patient coping skills, and some health care participants highlighted the emotionally challenging nature of oncology work.    66% of participants reported physical and treatment challenges which included specific difficulties related to surgery, chemotherapy, radiotherapy, pain and other symptoms, quality of life issues, socioeconomic struggles and the crisis of cancer progression.    61% of participants reported poor service challenges such as low cancer knowledge at primary care clinics and regional hospitals, broken equipment, testing delays, referral backlogs, dirty facilities, and a lack of psychosocial and financial support services.  47% of patient/family participants lived within 50 km of an oncology treatment centre and 22% of this cohort reported transport challenges. 53% lived further than 50 km and up to 1045km from a treatment centre, with 47% of this cohort reporting transport challenges. Transport challenges included the discomfort of long journeys, long waits for transport, logistical problems, separation from family and transport costs.  The finance challenges theme (40%): Financial stress, loss of income, loss of employment, the burden of additional costs, difficulties accessing financial support and medical aid challenges  19% of participants emphasised information challenges which related to insufficient understanding of the early signs of cancer, little information about treatment and support services, language barriers and access to information barriers.  Receiving good information about cancer was noted as reassuring and empowering while 14% of participants indicated feelings of disempowerment due to inadequate discussion with clinicians, poor information about treatment options, lack of patient-centred care and unequal power relations in the medical setting.  Problems of cancer stigma were reported by 31% of participants. Beliefs that provoked stigma included the idea that cancer was contagious, caused by evil spirits, a death sentence and tainted those it affected. It was reported that cancer stigma led to families being reluctant to speak about cancer and contributed to patient-experiences of isolation, shame, fear, discrimination and rejection.  The schooling challenges theme was commented on by 10% of participants and highlighted stigma as a concern in the school environment for children who had cancer. Many children with cancer reported that they missed school life, and most parents were concerned about disruptions to schooling. Schooling concerns were aggravated by lack of hospital school services, shortages of hospital teachers, language barriers and children feeling too ill to learn. |  | Distress screening and psychosocial support services should be a standard of oncology care and subject to quality control;  Standards of patient-centred care in oncology should be improved through training and professional development; Targeted training in cancer-mindedness and clinical skills at the primary and secondary levels of care is strongly indicated; Regionally centralised oncology diagnostic centres would support efficient screening and testing; Public-private partnerships to broaden service networks and make cancer care regionally accessible; Transport services need to be a more extensive and patient-centred service; Financial aid systems should be routinely and easily accessible; African-appropriate public cancer awareness programs need innovative design and implementation; Collaboration with traditional leaders and healers is an appropriate response to cultural patterns of health-seeking behaviour; Vibrant hospital schooling that collaborates with home-school teachers is indicated. |
| Edwards, L.B.,    2018 | N=316 participants    Cancer Patients & Family Members: 286  Healthcare Workers: 30  Age Range: 8 months (represented by a parent) to 86 years old  Gender Breakdown: 138 males, 148 females  Geographic Representation:  157 participants lived >50 km from a treatment center    *Participant Demographics*  Most Common Cancer Types:  Breast Cancer (n=83)  Leukemia (n=29)  Prostate Cancer (n=22)  Cervical Cancer (n=18)  Head and Neck Cancers (n=14)  Kidney Cancer (n=11) | 1. Causes of Emotional Distress and Anxiety   Emotional distress due to acute and ongoing anxiety (163); not knowing enough (18), uncertainty about the future (12) and a fear of death (13); diagnosis (114); cancer stigma (25);  medical setting (8).  *The cancer journey is hard like climbing a mountain. [PvM251, non-Hodgkin lymphoma]*  *I was frustrated that the doctors did not explain what happened. I did not know how to tell the rest of my family how she had died. [Pv74, advanced breast cancer]*  *When I wasfirst diagnosed, it felt like the death sentence. I was very traumatised and worried. [Pv186, prostate cancer]*  *Family don’t like to come to my house, some think they will also contract cancer from me. I feel very hurt and sad. [Pv176, ovarian cancer]*  *With my people if you have cancer of your private parts people in the community sometimes don’t want to visit you and say that you are no good anymore. [Pv161, metastatic breast cancer]*    2. Emotional challenges of the cancer process  Emotional struggle of daily hardships and physical challenges (53); Disruption to sense of a ‘normal self’(20); Emotional distress of loss issues due to cancer; Emotional considerations of advanced cancer (41); Emotional support challenges; Emotional impact of cancer on the family (70).  *Work, school, wife in hospital and two little kids, a daily struggle! [Pv57, wife died from cervix cancer]*  *Supporting a family is a huge weight on the shoulders of cancer patients who are breadwinners. [Pv29, jaw cancer]*  *My cancer has spread to my left lung. It was a shock that hit more than the first shock of being diagnosed with cancer. (Pv55, breast cancer)*  *I needed counselling but I never got that kind of support. [Pv74, breast cancer]*  *My family are not interested in supporting me. Nobody had time to talk or listen to me. [Pv85, colon & liver cancer]*  *My children are living separately back home with different family members. [PvM259, kidney cancer]*    Positive factors in coping with emotional challenges of cancer  Emotionally supportive factors: support of close family and friends, peer-patient, support groups, community, caring medical staff, workplace or school  *I joined the cancer support group at the hospital and never looked back. [Pv65, colon & lung cancer*  Factors nurturing a positive attitude: participating in valued activities, focusing  on the important things in life, caring for others, engaging with personal affirmations, understanding and acceptance of cancer, support from others, and spiritual belief.  *I struggled so much but I just prayed to God, fear can finish you.” [Pv306, breast cancer]*  The value of good medical care and interim home support  *I was diagnosed with cancer at the age of 26 and fortunately the doctors were very good and I was counselled by a great team. [Pv56, breast & colon cancer]*  *The interim care home is a good and safe place to stay while we have to be far from home for so long. [Pv28, jaw cancer]* | Limited representation of end-of-life patients.    Findings may not generalize beyond South Africa. | 1. Stress screening tools appropriate to the South African context should be implemented for the assessment and referral to specially trained nurses, social  workers and community workers, and cancer NGOs should collaborate with the state sector to augment psychosocial services  2. Strategic planning and implementation of ‘best-practice’ psychosocial standards of care in oncology  3. The issue of poverty should be prioritised for attention in underdeveloped countries so that the emotional pressure of socioeconomic crisis can be minimised for cancer patients and their families  4. Training, mentorship and emotional support systems for oncology workers need to be regarded as a priority out of respect for the requirement of health workers to be mentally healthy, competent, motivated and caring professionals  5. Initiatives for the promotion of patient empowerment and rights in the South African health-care context should be put onto political and management agendas and promoted in patient groups  6. Further research in the area of the ‘end of life’ phase of cancer, lack of public knowledge about cancer, social stigma, emotional isolation and lack of community and palliative care services |
| Georgievski, G., | N=6  Gender Breakdown: 4 female, 2 male  Age Range: 13-18 years old  Cancer Types:  Osteosarcoma (2)  Anaplastic large cell lymphoma (1)  Hodgkin lymphoma (1)  Acute myeloid leukemia (1)  Rhabdomyosarcoma (1) | 1. Physical Changes and Symptoms  Adolescents struggled with body image issues, especially hair loss and surgery-related changes. Feeling different from peers was a major concern, especially at school.  *“They don’t get it. They don’t understand what cancer treatment is or like what it’s*  *like to have a prosthetic leg or what it’s like to lose your hair. It’s a big thing for a girl.”*    2. Psychological Impact of Diagnosis  Participants confront fears of death, loss of  normalcy, and feelings of isolation, and psychological distress.  *“I am currently going through depression… But I go to school… I don’t know… sometimes I just go outside on one of the benches and I sit out there for that whole hour and half and I don’t even notice it because I’m so lost and like so lonely like I don’t have nobody to talk to”*    3. Short-Term Social Impacts of Cancer  Family and friends may mitigating or exacerbating distress, while still being the primary support and companionship.  *P5: I thought I was gonna be taking pictures of more friends and family but some family I consider as my best friends so … yea it just ended up being majority of my family than my friends … . in the beginning there’s always people who are like ’let me know if you need anything, I’m always here for you … .’ Then when push comes to shove and you ask them to be around and they’re not really here.*  4. Long-Term Social Impacts of Cancer  Impact on teenagers’ lives, particularly around their ability to engage with academics, extracurricular activities, and peers. Despite challenges in meeting the demands of school, our participants were often hopeful for their future, drawing inspiration and motivation for their careers and future aspirations from their cancer journeys. Fear still exists.  *P5: … hopefully one day I want to become an oncologist and work at SickKids … . I feel like I can relate to [children with cancer] more than just a regular person who hasn’t gone through this but I also want to try to cure it.*  P3: So now because like chemo ruins you and stuff, like I have a fear that I may never be a mom … . And because everything happened so quickly with me, we didn’t have time to save an egg or whatever … .  5. Cancer’s Impact on Holistic Well-Being  Faith, spirituality, and family support helped adolescents find meaning and resilience.  *P6: … this book is called the Qu’ran … . this is my book of hope … ever since I got diagnosed I tried to read it every day, I do read it every day. And one verse that I want to share with you that really gets to me and that … I think of all the time when, you know, I’m in sad times is [recites in Arabic], which is ‘surely after hardship comes ease.’ So every time I’m having a hard time with chemo, every time I’m sad, I just read … if not a page or two, a couple of verses that really strike me.*  6. Information Needs of Adolescents in Cancer Treatment  For many of our participants, meeting their informational needs involved respecting their autonomy and allowing them to decide how and when information is communicated to others.  *P1: I went a really long time without telling people and then one day my phone just started blowing up. Everyone was like ’Oh my God! I’m so sorry.’ I was like how did* you find out? And they’re like ’Oh they announced on the school like speaker thing.’ I was like ‘Ok I’m never visiting there ever again.’ But basically they told my little sister what cancer was and before she didn’t know … . when she finally found out she came to me crying saying that she hopes that I won’t die one day because … yea. I feel like she didn’t really need to go through that. | Small sample size (n=6), all from a single hospital.    Potential self-selection bias (participants willing to engage in Photovoice may already be more open). | For many, the group was an opportunity to meet other teens with cancer and establish important friendships that extended beyond our group. This comradery helped to counter many of our participants’ experiences of isolation. They were able to foster an environment within the group that enabled many participants to openly discuss difficult and often traumatic experiences.  The group also provided an important avenue to explore the ways that clinicians and teachers can  better meet their informational needs. The insights that our teenagers provided will form the basis of many important practice and policy changes that can be implemented within our Oncology Department. |
| Hammond, C. | N= 58 participants  Gender Breakdown: All female  Age Range: 27–81 years old (Mean = 61)  Cancer Types:  Breast cancer (75% of cases)  Colon cancer, kidney cancer, skin cancer, non-Hodgkin’s lymphoma (25% combined) | 1. Suffering Without Support Leads to Cycles of Silence  Many First Nations women concealed their diagnoses due to fear, stigma, and fatalistic beliefs about cancer.  Some avoided discussing their illness due to past trauma (e.g., residential schools) and mistrust of healthcare institutions.  Example Quotes & Visual Representations:  *“When people hear you have cancer, they assume you have six months to live. They don’t even ask.”*  *(Photo of a closed door, symbolizing the silence surrounding cancer in the community.)*  *“I don’t talk to my sisters anymore because our family was ruined by residential schools. We never learned to communicate.”*    2. Community-Based Supports Can Disrupt Cycles of Silence  Cultural practices, safe spaces, and peer support helped women break the silence and seek help.  Events like cancer awareness walks and fundraisers allowed survivors to share their stories openly.  Example Quotes & Visual Representations:  *“Last year, I walked in the Relay for Life. That’s when I finally told people I was a cancer survivor.”* *(Photo of a survivor holding a T-shirt from the event.)*  *“My sister gave me a handkerchief when I lost my hair. That simple gesture gave me strength.”*    *Barriers to Supportive Cancer Care*  Historical trauma (residential schools, displacement, systemic racism) made some women avoid medical institutions.  Limited access to healthcare resources in remote communities made cancer care more difficult.  Financial burden: Some women sold personal belongings to afford travel to treatment centers.  Racism and discrimination: Some participants felt unwelcome in support groups dominated by non-Indigenous women. | Small sample size, limited to four First Nations communities.    Did not include male cancer survivors, limiting perspectives.  Findings may not generalize to all Indigenous groups outside of Canada. | Develop culturally safe cancer care programs that integrate Indigenous healing practices.    Train healthcare providers on historical trauma and First Nations perspectives on cancer.    Increase funding for travel and support services for Indigenous cancer patients.    Expand cancer support groups in First Nations communities.    Encourage open discussions about cancer through community-led awareness programs. |
| Jellema, P.    2018 | N=3    37, 57, 67 years old  Two females, one male  All received/is receiving chemo and radiation therapy. One also received surgery, adjuvant therapy and rehabilitation physiotherapy. | Key Findings on Cancer Care Environments (CCE):  1.Home, Transit, and Hospital  Gaining a sense of the ‘places of importance’  Cancer care environments are not limited to hospitals; they extend to homes, transport routes, and waiting areas. Participants indicate their sensitivity towards the facilities, atmosphere, art in the hospital, nature, lighting, temperature, acoustics, odours and ventilation. The environment gets supportive when patients can show initiative or exert some control there.  *It’s like a boundary you have to step over. And when those doors open … It’s like… they*  *come towards me those doors, as though they say ‘welcome, come in, come into this world’*  Experiences of transit  As familiarity with a space increases initial feelings of fear and stress are replaced with a sense of safety and routine. Hospitals can also be inconvenient. Appreciation (or frustration) is most often expressed about aspects of spatial organisation that relate to convenience and privacy. As the route and place become more familiar, going to hospital becomes certain and new habits even emerge.  *Lisa and her husband stop for coffee: ‘Our moment of relaxation’*  *The hospital for me is always… Yeah, it’s there. I don’t have any problems with it. I don’t know how better to say that. Yes, we go to the clinic. I know my way ‘round there.*  The importance and comfort of the home  While home provided emotional comfort, it also became a medicalized space (self-administered treatments). Patients reported feeling insecure about taking medication at home, away from medical specialists.    2.As a Result of Treatment  Cancer treatments changed how patients perceived spaces:  Fatigue altered mobility: Walking long distances in hospitals became difficult.  Privacy needs shifted: Patients wanted more secluded spaces for personal moments.  Social interaction was selective: Patients preferred peer support over socializing with outsiders. Some hospital settings encouraged peer interactions, while others felt isolating.  Shared treatment spaces provided opportunities for informal support networks.  *“My wife was coming to pick me up. Me and my suitcase. And then I do find the oncology department really far to go on foot. In fact I find it a bit far. With my suitcase, after packing everything I was fine. But then on the way it was like being knocked with a hammer. Just so tired, tired, tired..”* – Walter  *“You saw the other man glancing over, he looked, nodded, hello sir and then I thought yeah, a comment, you know something you say as a joke about what he did and then our conversation was launched … Partly that is about who I am but it’s also a space for that. You’re sitting close to each other the whole time and you can read a book or you can do what you like, it’s the same. But sometimes it’s fun to say something.”* | Limited by small sample size, lack of diverse representation, reliance on participant willingness to take photos. | Improve hospital navigation & signage for easier movement.    Enhance hospital aesthetics (lighting, seating, sound control) to reduce stress.    Design private, calming spaces for emotional support.    Facilitate peer interaction opportunities to enhance informal support in shared treatment areas.    Ensure home-based care feels secure, providing remote access to specialists. |
| Jellema, P.    2020 | N=15 participants:    5 cancer patients receiving treatment.    5 relatives of these patients.    5 care professionals working in oncology departments.    *Participant Demographics*  Patients: 2 males (aged 69 and 71), 3 females (aged 28, 42, and 42).    Relatives: Included spouses and a father.  Care professionals: Included an oncology department chair, nurse practitioners, and head nurses from outpatient clinics and oncology wards. | Containing and Mediating Confrontation  Confrontation refers to a contrast in the experience of cancer care facilities that induced stress and anxiety.  1.1 First Encounters: Initial hospital experiences often caused stress and anxiety. Patients described the abruptness of transitioning into a cancer diagnosis. *P5’s first impression of the outpatient clinic was ‘a hallway of horror’. ... By the time she arrived, the clinic was busy and many patients were already ‘hooked up’ for chemotherapy. She kept her head down but could not avoid a visual confrontation. ‘I first had to go by all the old people, all those drab-looking people who’ve lost their hair […] before I could talk to someone. I found that- I didn’t like that’*  Position of front desk, lighting, contact with the outdoors, and furniture, color, temperature, smells, perceived age or dirtiness of materials affect the experiences  *I wasn’t planning to tell you that but when I came here in the outpatient clinic for my chemo, I was given an infusion stand by my bed of which the bottom part was dirty. Then I thought … You could wonder, “is that going to affect your treatment?” Probably not, probably not. But it didn’t give me a good feeling.*  1.2 Routes and Transitions: The design and organization of hospital spaces influenced how patients experienced their care. Long hallways, unclear signage, and large hospital buildings led to feelings of disorientation and depersonalization. “The entrance had been  determined beforehand such that you immediately came to the right place. You don’t end up in the larger entity of the hospital campus”  1.3 Boundaries: The need for privacy was highlighted, particularly regarding medical procedures and emotional experiences. Care professionals also sought dedicated private spaces to recover from their emotionally taxing work.    Coping: limitations and opportunities  2.1 Routine and Flexibility: Over time, patients established routines and adapted to their spaces, making the unfamiliar feel familiar. Due to their changing physical and mental condition, some patients required an environment that was flexible. P3 could switch between ‘active zones’ when she was still able to work, and ‘quiet rooms’ when she felt so bad that sensory stimulation had to be minimized. For care professionals spatial flexibility was a natural way of responding to care demands: assessing situations and looking for, or negotiating about, available space.  2.2 A Different Atmosphere: Care professionals emphasized the importance of a homelike environment within hospitals, suggesting features like softer lighting, plants, books, and artwork.    Changeability  3.1 Corporeality and Identity: As patients’ bodies changed due to treatment side effects, their interaction with space also evolved. Physical discomfort, fatigue, and sensory sensitivity influenced their hospital experience.  3.2 Changing Environment: Hospitals constantly undergo renovations, which can disrupt patients' sense of stability.  Example: A nurse mentioned that her workplace once had a view of a pond, but temporary buildings obstructed it over time, reducing the pleasantness of the environment. | Small sample size (15 participants), limiting generalizability.    Possible selection bias, as participants were recruited from personal and professional networks.    The choice to include in the study a mix of cancer types and cancer care facilities was  both a strength and a limitation.  the specificity of the participants and their situations makes comparisons difficult    Did not include perspectives of patients who were too ill to participate, which may have influenced findings. | Improve spatial organization to minimize disorientation and stress.    Create homelike spaces in hospitals to enhance emotional comfort.    Increase flexibility in space use while maintaining spatial stability to ensure patients feel a sense of familiarity.    Pay greater attention to entrances, routes, and transitions, as these spaces significantly impact patient experiences. |
| Lopez, E.D.S.,    2005 | N=13 participants  12 were breast cancer survivors and 1 considered herself a survivor because her daughter had recently passed away as a result of breast cancer.  Gender Breakdown: All female  Age Range: 44–82 years old  Education Levels: Varied from 5th grade to a master’s degree  Time Since Diagnosis: 1 to 53 years  4 women had income levels below US$10,000, and 2 women had no health insurance  Cancer Treatment Histories:  All participants underwent mastectomy, except one who had a lumpectomy  Some received chemotherapy, radiation, or tamoxifen  Only one participant opted for breast reconstruction    One woman had already experienced one recurrence of her breast cancer and experienced a second during our study. | Social Forces  1.1 Stigmatizing beliefs about cancer:  cancer always leads to death, cancer is contagious, and Women who have had cancer are rejected by male partners cancer was something to be feared and kept a secret;  *“Mm hm, and they think it’s catching too because, you know, it’s a lot of people have cancer and friends pull away from them like they think it’s contagious [agreement from the group]. I have some friends, yeah, they won’t go visit them because they think just going to see them they will catch, get cancer.”*  1.2 Racial Discrimination: Participants faced racism and medical mistrust in local hospitals and communities  *“We have doctors that come from the North and doctors that come from a foreign state . . . They are real nice to us, you know. They don’t see us as being Black folks or African American people. They see us, look at us like human beings . . . because they [doctors] are sort of prejudiced here..”*  1.3.Cultural beliefs about African American women  Survivors felt pressure to be caregivers despite their illness.  *“To be frank with you, I didn’t want to die leaving my mother. I didn’t have sisters and brothers. And my two kids would have taken care of her, but not like I would..”*    Survivorship QOL Concerns  2.1.Seeking Safe Sources: *He has been real good to me. He don’t slight me because I’m Black.*  2.2 Role adjustment: *There are things that I know I can’t do and the doctor had told me that there would be some things that he would rather me not to do. You know, [lifting] heavy things. And at first I did do as I was always doing. And I learned very quickly that I could not do it. I could not move furniture like I always did.*  2.3 Comfortable with future: *And he [the doctor] said, “Two more years and we’re going to take you off the pill.” “Graduation!” I said, “Yeah, and I’ll be right here for you to tell me.”*  *And [my sister] said “Well sis, what are you doing?” I said “Exactly what the doctor says to do.” And from there I followed the doctor’s instructions . . . but I just knew I’d turn it over to the Lord. And he healed me!*  2.4 Serve community as role models  Participants felt a duty to educate others and dispel myths about cancer.*Because they see you and what you’re doing with your life because a lot of people they fear [cancer]. When they get it, they fear it. “Oh, I’m going to die.” Well, let them know that life don’t stop right there.*    How Survivors Address Their QOL Needs  3.1 Relying on Faith and Spirituality  *The morning that they took me down to the operating room, it was just like going to get my breakfast . . . because I turned it over to The Master.*  *And I’m special, I think of myself “I must be special,” you know. God kept me and I had no pain and no chemo, no radiation, nothing.*  *I would tell people. At first I said, “Well, why keep it [a secret]? I want them to see what God is doing for me and how I’m going on, how I look, keep going, you know.” So, that’s why I would tell it, you know, tell people.*  3.2 Maintaining Social Standing  *It’s the message they send. You can look at their facial expression and tell what message they’re saying to you, whether it’s positive or negative . . . and that doesn’t have to be White. It can be Black too.*  *So, we went to the White people’s support group. And I think they really didn’t want us there in a way. They said the next meeting would probably be down at West End Baptist Church. So I didn’t know no Black people that went down there. So they [the other survivors] said they didn’t want to go back no more.* | mall sample size (n=13), all from rural North Carolina.  Did not include younger survivors or men.  although many patients would like to discuss their religious beliefs with their providers, they are often reluctant to do so | Expand culturally tailored cancer support groups for African American women. The women in this study declined services available through providers and other sources whom they considered to be racist or a threat to their social standing. This was exemplified by the fact that few of the women ever sought support from their local cancer support agency, one of few cancer-related resources available in their rural communities.    Improve racial sensitivity training for healthcare providers.    Develop church-based cancer education programs.    Increase access to financial aid and transportation for rural survivors.    All the women in our study participated in a local self-help group for African American women and viewed their self-help group sessions as a unique time when they could express their concerns and share their experiences with other African American survivors. |
| Morrison, T.L.,    2014 | N= 10 participants  Gender Breakdown: All female  Age Range: 40–69 years old  White 9, Asian 1  Post-cancer reduction in family income  Insignificant reduction, or family income remained above $100,000 8; Less than $20,000 reduction 0; $20,000–29,999 reduction 1; $30,000–39,999 reduction 0; $40,000–49,999 reduction 1;  Education  College 6; University 0; Bachelor’s 3; Doctorate 1  Vocational sector:  Food industry 1  Professional/white collar 9  Time with employer prior to cancer  <2 years 1; 2–5 years 4; 6–10 years 1; 11–25 years 4  Cancer Types:  Breast cancer (7 participants)  Colorectal cancer (1 participant)  Pancreatic cancer (1 participant)  Hematological cancer (1 participant) | Reasons Why Survivors RTW  Return to normalcy: symbolize a return to health and well-being, resilience, being valued at work, meaningfulness of work, distraction from cancer, social connectedness, love of the job, and sense of identity.  *You know that look good, feel better thing? You have to ascribe to it a little bit in that when you go in and people say ‘‘I think you look amazing’’ and that kind of stuff and ‘‘You’re looking great.’’ It makes you feel better, you release endorphins, and then you just keep going. (Lisa)*  *One of my competitors had called all my clients and so I made the decision. ... I spent the whole weekend calling all my customers...[and] said it was going to be business as usual. ... Four or 5 days post-surgery when I still had the drains in place I went to my first ... meeting with one of my clients. ... Nobody knew.*  Timing of RTW  Independent decision-making: Most survivors received little guidance on when to return.Many experienced unexpected fatigue and cognitive challenges.  Gradual RTW was ideal: Those who phased their return had smoother transitions.  Any advice from health care professionals regarding the timing of RTW was perceived as cautionary: ‘‘Everybody told me, ‘Don’t rush, take it gradually.’ My physiotherapist kept telling me, ‘Don’t go too hard, go gradually’’’ (Jose´e)  *I took literally 6 weeks off. I think I probably could have come back after 5, to tell you the truth, but I had plans for that final week, lunches, meeting up with friends that you don’t normally [do] when you’re working, so I came back at 6 weeks. (Aliciya)*  I was thinking about going back to work the first time and then I delayed it. I felt like I had a huge weight on my shoulder [see Figure 2]. ... Once I spoke to the owner, he was totally okay with me delaying coming back and I felt like that weight [was lifted]. ... It was really a phew!  The RTW Process  Supportive employers eased RTW, while social discrimination created stress.  Lack of formal return-to-work programs left many survivors to self-advocate for accommodations. many participants were forced to use a trial-and-error approach to their RTW.  *I was at the foot of the bridge and I had no idea how long or how to get to the other side given the obstacles that were in my path. ... I would have been very happy had [my employer] been prepared to go across the bridge. With me. Follow me ... across the bridge. ... I would have been very, very happy to have led them across the bridge too but they didn’t show up.*  *But the reality of cancer is that you take a step up and then you take a step down. There’s never a clear path to the door; there’s always obstacles. I wanted to kind of represent that in a picture [see Figure 7] and I chose my front steps. ... [Now] I’m standing on the final step going on to the platform. I’m getting there.* | Small sample size (n=10), all female participants.  Findings may not generalize to male survivors or blue-collar workers.  Limited racial and socioeconomic diversity.  it did reflect a lack of consistency  across the sample. Further, the participant sample does not represent survivors who were unable to RTW | Introduce formal RTW policies that include flexible hours and remote work.    Expand access to mental health and peer support programs in workplaces. |
| Morrison, T.L.,    2015 | N= 20 participants  Gender Breakdown: 10 male, 10 female  Age Range: 30–89 years old  30–39yrs 1; 40–49yrs 3; 50–59yrs 9; 60–69 4; 70–79 2; 80-89yrs 1.  White 16, Asian 2, East Indian 2  Post-cancer reduction in family income  Insignificant reduction, or family income remained >$100,000 12; Less than $20,000 reduction 2; $20,000–29,999 reduction 1; $40,000–49,999 reduction 2; Income reduction due to retirement 2; Not reported 1.  Education level  Less than college 0; College 8; Below bachelors 2; Bachelors 7; Masters 2; Doctorate 1.  Vocational sector  Professional/white collar  (e.g., office worker, professor) 17; Food industry 1; Farmer (cash crop) 1; Heavy industry 1.  Time with employer at time of cancer diagnosis  <2 years 1; 2–5 years 5; 6–10 years 3; 11+years 11.  Employer post cancer  Returned to pre-cancer employer 2, Returned to new employer 0  Cancer Types:  Breast cancer (7); Prostate cancer (6); Colorectal cancer (3); pancreatic, melanoma, renal, sarcoma, hematological, 1 for each | 1. Motivations for Returning to Work  Social considerations: a sense of connection, support, and friendship inherent to their workplace 5W 5M  Identity 2W 2M  Financial/provider 3 3  Not financially motivated 1 2  Return to normal 5 4  Feels valued, meaningful position 5 4  Distraction 3 4  RTW is good, healthy 2 4  Show of strength 4 1  Love job and being at work 6 2  Productivity 0 6  *This represents my identity and work was who I was, so not unlike the name of a boat. That boat’s name is Iyega. Work was my identity. So, this kind of represents to me, you know it was truly who I was.*  The most significant difference in WI motivations related to a sense of productivity afforded by work. This was cited by six of the men and none of the women  *This is my basement…I realized that for 3 months…this kind of became my office. You can see my laptop...became my place to go for at least part of the day when I’d check email and just do pretty much nothing but it felt normal…It brought back a bit of the sense of just doing something with your day*  Women Talk, Men Do  Chris, an advanced prostate cancer survivor, reflected: “The first thing women do is talk about it. The first thing men do is decide what they’re gonna do.”  Women sought emotional support from family, friends, coworkers and peers, engaging in conversations about their experiences. Women’s openness to discussion with others thus challenged existing ideas and shaped not only their approach to both survivorship and coping in general but also their decisions related to WI.  Men found the multiplicity of coworkers’ questioning overwhelming, relied on activities rather than verbal communication, finding productivity-based coping mechanisms.  *We were having lunch together here in my office...We’re both kind of sharing this moment but she coming to my office and chatting with me for that hour, we do this every week…She was a colleague and now she’s a friend…These lunches gave me the opportunity to stop, to reflect, to chat, to integrate back into the work place in a really soft way. (Melanie)*  *I was still having difficulty walking…We bought the wood. . .then we had to cut it, shape it, put it all together…after it was assembled, we had to sand it and oil it… I’d sit down there and sand for hours. There was nothing else to do because I couldn’t do anything else, so I’d sit in the living room, dust all over the place, smoothing that clock out and it worked out pretty good, I must say…that was really a big part of my recovery period and I found it was therapeutic as well as it took your mind off things.(Austin)* | Small sample size (n=20), primarily white-collar workers.   challenge of recruiting male participants  Issues raised by the men were unable to be discussed with the female participants.  Limited racial and socioeconomic diversity.    Findings may not generalize to blue-collar labor-intensive jobs. | Develop gender-sensitive work reintegration programs.    Encourage activity-based coping strategies for male survivors.    Enhance social and emotional support networks for female survivors.    Educate employers on cancer-related work challenges and accommodations. |
| Morrison, T. L.    2015 | It seems that same as above? | 1. Privacy and Camouflage  They may choose to maintain complete privacy or share only with those closest to them; camouflaged their social and professional presentations.By camouflaging their appearances and ensuring consistency in their behaviour, Lisa and Mary achieved their objective of being treated normally by others. This reinforced their desire to maintain their ordinary healthy, pre-cancer identities.  *More than half the people I met never saw an IV tube coming out of my purse. It looked goofy to walk around the office with a purse over your shoulder but this is where I hid my antibiotic pump...I would have the IV tube coming out and it would come across my back usually under my sweater... . Most people wouldn’t have known that I was hooked up to antibiotics... . [The pharmacy] gives you this lovely blue fanny pack ... . It just draws attention to the idea that you’ve got something going on there. ... [Using my purse as a pouch bag] was definitely the freedom to go and not look sick, right? If you go in with this tube sticking out of your arm and it’s apparent ... people will think it’s inappropriate that you’re there. [Lisa, Fig. 3]*  *No one ever needed to use my doorbell while I was at home during chemo because I just kept everything open because I couldn’t make it down the stairs anyway. What I wanted it to represent was this difference in privacy. When you become a patient, privacy changes. Your body is not that private entity anymore ... your*  *private struggle ... everybody knows about it ... your private life becomes part of your professional or public life (Fig. 1). (Melanie)*  2. Process of Disclosure  Most participants disclosed their diagnosis to at least some colleagues or supervisors. Some chose selective disclosure, telling only close colleagues.  Others took an open and proactive approach, informing clients and employers widely.  Disclosure is affected by work type and nature.    3. Educating Others  Survivors who openly discussed their cancer often took on an educator role.  Some created online resources (e.g., YouTube videos explaining treatment).  Others provided direct guidance to coworkers about how to interact with them post-diagnosis.  “Crawl out of bed ... go into the  office and greet [your] colleagues and get that barrier down and maybe let them participate more in the treatment ... help them to understand ... don’t go in there looking for sympathy ... go in there with information  to educate them ... . It’s also for me as a person who is not well, it makes me more comfortable walking into a room where I can come in and just say, “Hi,” and carry on.”    Benefits of privacy: maintenance of pre-cancer identity, maintenance of sense of conpetence, treated normally because others are not uncomfortable, work environment can provide a temporary distance from cancer-related concerns, not required to receive others’ questions, concern and physical interactions  Benefits of disclosure: support from others, cathartic for the survivor to discuss, demystifies absence, enhances social ease of others, provides an opportunity to educate others regarding survivors’ needs and expected behaviour,facilitates work integration by allowing collaborative,  customized, creative planning    Cons of Camouflage: limitations are forgotten or unknown by others, lack of recognition/support, inability to accommodate without knowledge    Cons of Disclosure: define limits of disclosure, define limits of requested privacy, repeated questioning/concern can eliminate benefits of RTW | Small sample size limited generalizability.    Participants were mostly professionals with high incomes,  for most a RTW was not necessary for sheer survival and work was not physically demanding.limiting applicability to other job sectors.  inclusion of participants from lower socioeconomic classes, those with lesser education, and from various employment sectors and geographical locations. | Survivors should have access to disclosure coaching to navigate workplace conversations. |
| Mosavel, M.,    2010 | N=17 participants  All African Americans  Gender Breakdown: 13 female, 6 male  Age Range: 23–60 years old (Mean = 45)  7 survivor and 13 caregiver  Employment & Income:  two owned their homes, the others rented  77% unemployed at the time of study  Over 50% had a household income of less than $5,000 per year  Education Levels:  67% completed high school  47% attended some college  Marital Status:  18% married  33% single 23% divorced 5% widowed | 1. Financial Concerns  Cancer treatment costs were overwhelming, with many participants unable to afford medical bills, transportation, or medications.  Survivors skipped doctor’s appointments due to financial strain. Some had to rely on home remedies because they couldn’t afford prescriptions.  2. Lack of Social Support  Survivors experienced isolation from family and friends, often due to stigma surrounding cancer in the African American community.  Religious beliefs sometimes conflicted with seeking medical treatment, as some saw cancer as a test of faith.  Some lost friends and support networks after diagnosis. They talked about the inability to communicate their own needs, which was further exacerbated by the silent response of others.  Several caregivers shared through pictures that the inability to provide for their loved ones caused them to feel guilty and subsequently led to a very difficult emotional time for them.  An important aspect of social support that was reported missing was a social support for children of cancer survivors.  3. LACK OF CONTINUED CANCER CARE  Survivors felt that once treatment ended, they were left without guidance on maintaining their health.  Doctors rarely discussed post-treatment care, such as nutrition, exercise, or mental health.  Some questioned if healthcare providers assumed those living in the inner city wouldn’t be interested in healthy living advice.  4. Perception Adjustment  Breast cancer survivors struggled with body image, particularly hair loss, mastectomy scars, and lack of access to wigs for Black women.  5. Lack of Neighborhood-Based Services  Many services (e.g., support groups, exercise classes, mental health counseling) were not located in their neighborhood. Services that were welcomed were transportation to hospital appointments, hospice service, and meditation. Survivors wanted services closer to home that were culturally relevant and welcoming. | Small sample size (n=20), primarily female participants.    Findings may not generalize to rural Black communities.    Did not focus on a specific cancer type. | Increase financial aid programs for African American cancer survivors.    Develop culturally relevant survivorship support groups in urban Black communities.  Improve access to affordable wigs, makeup, and appearance-related resources.    Provide better education on post-treatment care (e.g., nutrition, exercise, mental health). |
| O’Callaghan, N.    2024 | N=8 participants  Gender Breakdown: 7 women, 1 man  Age Range: 45–59 years old (Mean age: 51)  Education Levels: All participants had higher education (third-level degrees)  Employment Status: Half of participants were employed full-time  Cancer Types:  Breast cancer (7 participants)  Prostate cancer (1 participant)  The majority (n=7) had completed treatment in the last 5 years | 1. Fresh is Best  fresh, organic or locally sourced food,,going ‘back to basics’, explaining, *‘I went back doing what our parents ate, everything fresh and out of the ground—no added preservatives. We eat as natural as we can, and the fewer processes that food goes through, the better it is for you.’*  2. Building Blocks. Be Informed.  Many believed cancer was a "teachable moment" for changing dietary habits. While the group views on dietary choices differed, the cohort did note the importance of being adequately informed and build on nutrition knowledge.  3. Be Kind to Yourself  Food was a source of celebration for life milestones after cancer. One individual shared an image containing ingredients for a family roast dinner highlighting: *‘an old-fashioned Sunday roast, good food and good company’.*  4. Post-Treatment Healing Changes  Participants made specific dietary changes to promote healing.  Figure 4 relates to one participant cutting dairy out of her diet initially post-treatment and now only occasionally eats dairy.  5. Chemo Rituals  Certain foods were linked to chemotherapy experiences and persisted as habits post-treatment. One participant presented an image of coconut water where she shared her chemotherapy experience: *‘every session before chemo, I was drinking litres of coconut water, research in the UK has shown your blood count will never be wrong for chemotherapy’*  6. Food for the Soul–Healthy Mind, Healthy Body  Participants saw exercise and nature as essential "nutrition" for mental health. *‘These things are nutrition in their way as well, nutrition for the soul, it’s not just the food, I mean the food is important, but it can’t be in isolation, you know?’* | Small sample size (n=8), primarily female breast cancer survivors.    Only one male participant,    Higher education levels than the general survivor population. | Enhance access to nutrition counseling for cancer survivors.    Encourage self-directed nutrition education while promoting scientific accuracy.    Acknowledge the emotional and social aspects of food in survivorship care. |
| Pailler, M.E. | N=30 participants (T2: 14+16 partial, T3: 12+13 partial)  Age Range: 17–36 years old Mean(SD) = 28.23(5.29)  Gender Breakdown: 23 female, 7 male  Race: White/Non Hispanic 25, Black 2, American Indian 1, Latino 2  Cancer Types:  Leukemia/Lymphoma (12, 40%)  Papillary thyroid carcinoma (6, 20%)  Cervical cancer (4, 13.3%)  Melanoma (3, 10%), breast cancer (3, 10%), sarcoma (1, 3.3%), and brain (1, 3.3%)  Treatment Histories:  11 (36.7%) combination  10 (33.3%) chemotherapy only  8 (26.7%) surgery only  1 (3.3%) radiation    Distance to center  <15 miles: 13 (43.3%)  >15 and <100 miles: 14 (46.7%)  >100 miles: 3(10%)    Beaupin 2019  N=13    Gender Breakdown: 83% female  Age Range: 17–26 years old (Mean = 23.15, SD=2.76)  Cancer Types:  54% Leukemia/Lymphoma  23% Papillary thyroid carcinoma  15% Melanoma    47% lived beyond 15 miles from the cancer center.  Two most active participants lived 75 and 504 miles away. | 1. Impact of Social Media-Based Photovoice on Mental Health  Participants experienced a decrease in depressive symptoms from pre- (57.23 ± 10.35) to post-intervention (51.85 ± 8.53), t(12)=2.48, p=.03; and from pre- to 2mo after intervention (52.00 ± 10.14, t(12)=2.94, p=.01). Similar findings for QoL (T1 74.42 (13.71) vs T2 81.73 (14.88), p=.02, T1 74.42 (13.71) vs T3 80.83 (14.11), p<.01). No significant changes in spiritual well-being.    Themes: a sense of connectedness with others who have shared experiences, enjoyment of the weekly themes, and knowledge that  others struggle with similar issues. The most consistent negative feedback involved  the functionality and flexibility of the application itself, with 71% of respondents reporting challenges with the application. The two noncompleters who provided feedback cited health concerns and not enough time as reasons for not participating. Regarding preferences for typing versus audio-recording the weekly assignments, 41% of participants indicated a preference for texting responses, and 47% described being comfortable or very comfortable with audio-recording narratives. Of note, during the semi-structured interviews, several participants indicated that they found the audio recordings more meaningful, but they were more likely to read the text than listen to the audio given the time involved and the necessity of privacy or headphones for the audio narratives.  Engagement with peers through social media helped reduce isolation and improve emotional well-being.  Photovoice helped AYAs process their emotions through self-expression and storytelling.  Example Quotes & Visual Representations:  *“Sharing my pictures and stories helped me realize I wasn’t alone.”*  *“I didn’t have a support group near me, but this program gave me a virtual one.”*    2. Social Connection and Community Through Photography  Posting photos and receiving comments from other AYAs created a sense of belonging.  Participants felt understood without needing to explain themselves in traditional therapy.  Engagement rates were higher among female participants, with men less likely to complete the intervention.  Example Quotes & Visual Representations:  *“It was easier to express my thoughts through photos than talking about them.”*  *“Seeing others’ posts made me feel seen. I wasn’t the only one struggling.”*    3. Meaning-Making and Coping with Cancer  Weekly themes (e.g., *Living with Cancer, Coping with Bad Days, Hope and Uncertainty*) helped participants reframe their experiences.  Many participants found new meaning in their journey through photographic reflection.  Themes of resilience and identity transformation were common in participants’ photos.  Example Quotes & Visual Representations:  *“Cancer changed me, but I am learning to redefine myself.”*  *“There’s light at the end of the tunnel, even if you can’t always see it.”*      Beaupin 2019  Qualitative responses in the follow‐up interview were also positive. Feedback statements included terms such as “pretty awesome, I was really happy to be a part of it” and expressed an appreciation for the opportunity to connect and share experiences with others. The only negative feedback came from frustration associated with technical problems recording their narratives    *Week 1: Who am I?* → *“Happy … I wasn't going to let cancer define me. I was going to continue to be happy”*  *Week 2: Living with cancer, how has*  *cancer changed who I am?* → *“My scars from melanoma and it's a daily reminder of what I went through ….”*  *Week 3: What matters most?* → *“… just being happy. Waking up and being happy every day …. I choose this picture because I was extremely happy. I left all of my problems at the shore.”* | Small sample size (n=30), predominantly female.    the lack of control group limits conclusions about the role of the intervention in causing changes in outcome variables, although the stability of changes from T2 to T3 is encouraging.    eligibility requirements such as access to a smartphone or web browser may have limited study participation.  Limited long-term follow-up beyond 2 months.    Relied on self-reported measures, subject to bias. | reaching out to AYAs through  SM and other online resources    Expand online psychosocial programs for AYA cancer survivors.    Encourage social media-based support groups for AYAs in rural areas.    Integrate Photovoice into broader survivorship care models.    Develop gender-specific engagement strategies to encourage male participation. |
| Park, J.S. | N=9 participants  Age Range: 53–65 years old  Cancer Stages:  Stage 1 (3 participants)  Stage 2 (4 participants)  Stage 3 (2 participants)  Treatment Histories:  8 total mastectomies, 1 subtotal mastectomy  5 participants received chemotherapy only  4 participants received both chemotherapy and radiation therapy  Tamoxifen Therapy Duration: 5–8 years  Weight Gain Post-Treatment: 2–10 kg (Average = 4.8 kg) | 1. Willingness to Live  Struggle to live a day without pain  Willingness to live for family  *“To me, weight management means to live a healthy life even if I just have one day left to live. My goal is to live a healthy, pain-free life for at least a day rather than living long.”*  2. Trying to Change Daily Routine  Finding low-cost physical activities  Accessing healthy food  Showing interest in one’s weight  *“The scale helps me maintain proper weight. I weigh myself once a day. While weighing myself, I also check the daily amount of food eaten or amount of exercise, along with plans for tomorrow’s weight management.” (Participant 5)*  3. Individual Difficulties in Weight Management  Frequent patients’ meetings  Difficulty in coping with stress  Limitations of self-planned weight management  *“It is the stress from my in-laws that prevents me from getting my weight under control. I turn to binge eating when stressed out.”*  4. Support System for Weight Management  Social support for weight management  Family support for weight management  *“Energy exercises worked just great for me in managing my weight. But, there is no detailed information about which public healthcare center provides this program. I wish it was made easily accessible.”(Participant 5)* | Small sample size (n=9), limiting generalizability.    Focus on Tamoxifen-treated patients only—excludes experiences of those on other hormone therapies.    Did not account for long-term outcomes of weight management interventions. | Long-term support for weight management    Enhance access to community fitness and nutrition programs tailored for survivors.    Provide psychological support for stress-induced eating behaviors.    Encourage family involvement in weight management efforts. |
| Poudrier, J. | N=12 Aboriginal women  Age Range: 42 to 75 years old  Residence:  3 lived in urban settings  9 lived in rural areas or First Nations communities  Family Roles: Many were mothers and grandmothers    *Participant Demographics*  Breast cancer survivors who had completed treatment.  Varied cultural backgrounds, representing different Indigenous communities.  Some had strong ties to traditional Aboriginal spirituality, while others identified more with Christian beliefs. | 1. Spirituality and Aboriginal identity  in the experience of breast cancer  Traditional and Spiritual Healing:  Some women integrated sweat lodges, smudging, and traditional medicines into their healing process.  Others combined Christian faith with Aboriginal traditions.  Symbolism of Hair Loss:  For some, hair loss was more than a physical change—it symbolized mourning and death in Indigenous traditions.*‘the only time that, people cut their hair is when they lost loved ones, and that’s what I believe today.... When I lost my hair, I cried. I cried. ‘‘Well now, maybe I’m gonna die’’ I said to myself.’*  *“The sweat lodge ceremony is a big part of my life and that’s where I did most of my healing ... Anything and everything in there is safe. Everything in there is equal because when that door is closed its black. So nobody, there’s no color. There’s no racism in there. There is nothing in there. It’s really like a positive experience. (Fig. 4)”*  it would be very important for healthcare providers to have an awareness that the  experience of hair loss is may be profoundly frightening for some Aboriginal women and that it may also be linked to the uptake of chemotherapy and western medicines generally    2. Multidimensional Support  Support groups and services  existing breast cancer support groups did not necessarily meet their needs; Cheryl was heavily involved in an exercise group for women with breast cancer and felt that support was really about individual experience and attitude, rather than connections to Aboriginal identities and experience; they felt it was important to have support groups  for Aboriginal women or those which reflected their lived realities; and they felt strongly about offering that support Aboriginal women, or men, who are newly diagnosed with cancer.    Support from healthcare professionals  we see a range of medical encounters which are both affirming (the ability to actively participate in healthcare decisions through respectful communication, the affirmation of personal and cultural identity and the development of positive, long-term relationships), and invalidating (experiences of racism, dismissal, vulnerability and a lack of understanding).  their relationships with healthcare professionals were important in two areas: (i) understanding Aboriginal women’s lived experiences, and (ii) communicating in ways that are supportive, straightforward and reflect some sensitivity to racism.    Support for information  women needed information that was relevant to their lived reality, including their socioeconomic situation. Many women indicated that they did not always understand what was being explained and they did not seek clarification for fear of looking unintelligent.  *‘The surgeon who speaks to you, the doctors, the medicines you’re taking — the words they use are commonplace in their daily life. But in ours, they’re not.’* | Small sample size, limiting generalizability.    Findings specific to Saskatchewan, may not reflect other Indigenous communities. | Train healthcare professionals in cultural competency to reduce racism and improve patient communication.    Develop Aboriginal-specific breast cancer support groups to create safe spaces for survivors.    Implement patient navigation programs led by Indigenous healthcare workers.    Relevant support could take the form of (i) support groups  specifically for Aboriginal women; (ii) respectful communication strategies with healthcare professionals; (iii) and the ongoing availability of information that is relevant to their  lived experiences |
| Power 2022 | Forty-five patients and ten carers  No separate demo info for the photovoice group | 3.1 Living With Minority Stress: Discrimination, Exclusion, and Fear of Hostility  3.1.1 Homophobia, Transphobia and Prejudice When We Were Younger: An Era of “Open Discrimination and Hostility”  *It’s taken me a long time [to embrace my sexual identity] because I grew up in the 70s seeing my friends who were gay being targeted. One guy I worked with took his own life. He had a record because he was involved in a sexual act and that was illegal at that stage. So, seeing all of these things, all of these repercussions, I sort of thought I need to squash the gay side of me.*    3.1.2 The Legacy of Discrimination: Fear of Hostility and Self-Loathing  *Used to carry around our marriage certificate everywhere [during cancer care]. We changed our names as soon as we could so if we had to, we could pass off as sisters. We did what we could so that we could make sure that everything was ok. It was a safety concern.*    3.1.3 “It Still Goes On”: Living With Discrimination and Prejudice Today  *I’ve been assaulted seriously in the last 24 months, six times. Last year, someone attacked me in my own front yard in daylight. Bathroom use has also been ‘problematic’. I have been verbally abused and physically assaulted on several occasions inside my local shopping center.*    3.1.4 “The Possibility of Being Judged”: Fear of Exclusion and Discrimination in Cancer Care  *The world we live in - read the news - affects our health. The more we know we are hated and feared (religious freedoms act; trans hate; The Australian [a News Corporation newspaper]), the less likely we are to access care or feel safe when we do. One of my specialists was showing me an app on her phone and it was surrounded by Christian apps and Bible apps, and I was instantly terrified and will not go back. (Survey, 40, intersex, queer, medical intervention)*    3.1.5 “Treated Abominably”: Family and Community Rejection Exacerbates Distress  *There just aren’t many people, in general, around that area. You know, you could go into town and there’ll be a few people, but none of them would be gay. So, meeting other gay people, that wasn’t really an option, where I lived.*    3.2 Resisting the Margins: Social Support and Activism Buffer the Effects of Minority Stress  3.2.1 “Help in the Most Magnificent Ways”: Family, Friends, and Community Support  *It’s really shown what an incredibly resilient couple we are. It’s really brought forward all of the beautiful things that have underpinned our relationship over the last 24 years. They suddenly light up large in big print. The reasons that we have worked so well as a couple.*    3.2.2 Collective Action to Resist LGBTQI Discrimination and Exclusion in Cancer Care  *I came out to so many people in the hospital – staff and other ‘cancer families’ – often for the reason of increasing visibility of rainbow families in the hope that this would reduce homophobia, and increase people’s awareness of rainbow families and just how similar/normal we are when compared with families headed by a heterosexual couple or heterosexual single parent (Survey, parent, 47, lesbian, leukemi*a) |  | professional education and training to create environments in which LGBTQI patients and carers access care and support during cancer need to be culturally safe and inclusive  The inclusion of partners and chosen family in consultations and support services is also essential (27). There is also a need for investment in peer-led initiatives that provide connection and support to LGBTQI people with cancer and carers to help to overcome barriers to social support. Minority stress is pervasive in the lives of many LGBTQI people. We have a duty of care to ensure that the impact of minority stress on wellbeing and on interactions with HCPs is recognized and addressed within professional cancer care. |
| Wong, S.S.,    2019 | N=20 participants (13 patients, 7 caregivers)  Pancreatic cancer  Age Range of Patients: 40–79 years old  40-49:1; 50-59: 6; 60-69: 11; 70-79:2.  Caucasian 19, other 1  Gender Breakdown: 11 female, 9 male  14 partnered, 6 unpartnered  Tumor Resection Status:  Resectable tumors 13  Non-resectable tumors 7  Treatment Status:  In active treatment 15  Under surveillance 5 | 1. Diagnosis of an Unexpected Advanced Cancer  Patients and caregivers struggled with the shock of the diagnosis, as pancreatic cancer is often detected at an advanced stage.  Anger was the predominant emotion rather than sadness, as patients felt betrayed by their bodies.  Medical professionals' focus on end-of-life care at diagnosis reinforced feelings of hopelessness.  Worry about prognosis and treatment outcomes  *The fear of the unknown, the fear of not being sure of how it’s going to happen and how I’m going to react…I’m afraid of losing him…I’m worried about how I’m going to feel…What the hell is gonna happen to me? I have to stay here (Jacklyn).*  2. Struggles with Weight Loss and GI Symptoms  Managing gastrointestinal (GI) issues was a major source of distress for patients.  Severe nausea, diarrhea, and difficulty eating left patients feeling physically weakened and emotionally drained.  Patients were frustrated due to loss of control. Caregivers felt helpless watching their loved ones struggle.  *“I would take whatever meal I wanted to eat and I would put it in a blender. I would add water or chicken broth or beef broth and I would drink it. And you know, if that’s the way I have to survive, then so be it.”*  3. Changes in Roles and Identity  Patients struggled to maintain their identity; for some, cancer made them dependent on caregivers.  Caregivers experienced burnout, feeling overwhelmed by medical responsibilities while trying to maintain normal life.  *“I’m nobody’s hero, I’m nobody’s idol. I’m not anyone to be looked up to. I’m just a guy trying to survive” (Brandon).*  *“Even when you go in and see Dr. X or any of the other providers, it’s about them [the patients]. Nobody says how are you doing?” (Gretchen)*  4. Fear of the Future  Even patients with curable tumors feared recurrence. Feel insecure without weekly check-ups with medical care team.  Caregivers lived in a constant state of hypervigilance, monitoring every symptom for signs of relapse.  *Jacklyn presented a photograph of a dark cloud (Fig. 4) to signify the constant*  *overshadow of doubt and worry:*  *“No matter what kind of wonderful day you’re having, you know that these black*  *clouds are there and on any day, life could change again in a minute. So you never*  *ever really are without feeling that.* “ | Sample may have a better prognosis than general pancreatic cancer population. Primarily Caucasian participants, limiting generalizability to diverse populations.  additional themes may have been missed for  caregivers who were unable to attend clinic visits. | Improve how prognosis is communicated to reduce initial emotional distress.    Develop nutrition-focused interventions to help patients manage weight loss and GI symptoms.    Provide caregiver support programs to prevent burnout and role strain.    Create long-term survivorship plans for patients with resectable tumors to address fear of recurrence.    Encourage peer-support programs for patients and caregivers to share coping strategies. |
| Wong, C.L.    2024 | N=34 participants (17 survivors, 17 parents)  Gender Breakdown:  Survivors: 10 female, 7 male  Parents: Predominantly mothers  Age Range of Survivors: 8–17 years old (mean 14.2 yrs)  Cancer Types Represented:  Leukemia (ALL, AML) 10  Neuroblastoma 1  Rhabdomyosarcoma 2  Wilms tumor 1  Osteosarcoma 1  Medulloblastoma 1  Astrocytoma 1  Age of diagnosis 3 months -9 yrs (mean 3.3 yrs) | 1. Indelible Marks  Survivors and parents carried emotional scars from the cancer journey.  Parents supremely concerned with the treatment and cannot leave the children alone. The concern carried over time even after cure.  Parents struggled with emotional flashbacks over years. Survivors may not.  *P17: After the cancer treatment, I felt like I had fallen into another abyss. Whenever she comes home late, I get scared, worrying that the worst will happen to her and the possibility of losing her.*  *P12: It feels like it was a long time ago, but sometimes these emotions pop up. I always think back to the days when I was sitting in a hospital chair in despair(Figure 1c).*    2. Struggling With Late Effects  Survivors experienced ongoing physical effects, including fatigue, pain, growth issues, and weakened immunity.  Fear of recurrence was a persistent concern for both survivors and parents.  *P10: My child frequently feels ill, often missing several days of school each month. His immune system is a bit weak. When his classmates were doing activities like skipping rope, he often stood there looking listless, lacking energy and initiative.*  *S5: I always afraid of having another seizure, not sure if the cancer will come back.*  3. Striving for Normalcy  Some survivors considered cancer as opportunities for personal growth, facilitating reintegrating into normal life.  Some embraced physical changes, while others hid their past to avoid stigma.  longing for returning to school and social life  *P10: Although his legs can no longer move due to the disease, he has quickly adapted to using a wheelchair and bravely races against fate with his hands (Figure 3a).*  *S2: Even though my legs are different lengths, I try not to use any assistive devices and live a normal life (Figure 3b).*    4. The Strength of Support  Religious faith provided emotional strength during and after treatment.  Healthcare providers, particularly nurses, were key sources of comfort.  family members, fellow parents in the same ward, friends, and non-governmental organizations dedicated services to childhood cancer    5. Living in the Moment and Hope for the Future  Survivors and parents cherished life more deeply and set ambitious goals for the future.  Empowered by their experiences, survivors hold a strong belief in their ability to fight for anything and to confront further challenges and difficulties.  Many wanted to give back by mentoring other young survivors.  Parents prioritized family time, appreciating each moment together.  *S13: We can all go through the most difficult experiences of fighting cancer, and there’s nothing we can’t go through (Figure 5c).*  S13: I hope to become a doctor in the future so I can share my experiences and encourage others who are still battling cancer (Figure 5e).  P10: My little boy has transformed into a young man. I hope he can take off with his sports dream! (Figure 5f). | Small sample size (n=40), specific to Hong Kong.    no further comparative analysis was conducted to explore any thematic differences based on age    survivorship challenges and priorities could evolve over time, the feelings, experiences, and perspectives of childhood cancer survivors may vary | Develop structured survivorship programs to address late effects and emotional well-being.    Promote peer mentorship programs to help new survivors navigate post-treatment life.    Integrate faith-based and cultural support systems into survivorship care. |
| Yi, J.,    2010 | N= 12 participants (6 survivors, 6 family members)  Gender Breakdown:  Survivors: 4 female, 2 male  Family members: Mostly parents, one spouse  Age Range of Survivors: 18–39 years old  Cancer Types Represented:  Acute lymphoblastic leukemia (ALL)  Osteosarcoma  Brain tumor | Themes from survivors  1. Lost Childhood  Remembering hospital days  Missed experiences  Inside and outside     1. My culture   United family, Healing food, Religion, Rituals, Traditional remedies     1. Health   Impact of cancer on family  Lingering cancer  Thinking about death and life     1. WHAT KEEPS ME GOING/SACRIFICES   Family, people, future     1. Who am I 2. Survivor, I am morethan what I look, family and people     Themes from the Family Member Group  1. How cancer affected survivors’ hopes and dreams  Sacrifices to cancer, still a kid, survivors’ altruism   1. Positive impact of cancer   Religion, faith, and hope helped, give and take   1. Importance of information   Information needs   1. Barriers to self-care   Caregivers’ health, lack of self-care, other family issues   1. What we learned and what we can do   Therapeutic effects of the projects, what we can do | Small sample size (n=12), primarily Latino participants.  Sample was were relatively healthy volunteers and actively involved in the local community-based agency’s (PADRES) activities. | Develop structured programs for young adult cancer survivors to support career development and social reintegration.  Provide caregiver-focused interventions addressing stress and emotional exhaustion. |
| Yi, J.,    2016 | N=7 participants  Gender Breakdown: 4 female, 3 male  Age 20-27 yrs, mean 23.71 yrs, SD 2.75  Education:  2 current university, 5 at least some college  Cancer Types Represented:  Acute lymphoblastic leukemia (ALL) 2  Malignant lymphoma 1  Osteosarcoma 1  Neuroblastoma 1  Brain tumor 1  Ovarian cancer 1    *Participant Demographics*  Age at Diagnosis: 5 to 15 years old, mean 11.43, SD 3.36  Time Since Diagnosis: 8 to 17 years post-diagnosis, mean 12.29, SD 3.15  Recurrence: Two participants had relapsed  Living Situations: All participants resided in Seoul or nearby metropolitan cities  Marital Status: All were unmarried | 1. Relationships With Others  Parents:  Survivors expressed deep gratitude for parental support but also felt a sense of obligation to pay back what they received from their family  Romantic Relationships:  Many struggled with self-esteem and feared rejection due to their cancer history.  Some avoided relationships altogether due to concerns about genetic risks for future children.  Friendships:  Participants had difficulty forming friendships due to missed social experiences during treatment.  Many felt isolated and only confided in other cancer survivors.  Example Quote:  *“My relationships were complex and tangled like a maze. I had a gap in my life.”*    2. Stigma  Inadequate: Men exempt from military service (due to cancer) felt excluded from male social circles.  Gloomy: Survivors were often seen as physically weak.  Being less competent: The participants agreed that people do not have high expectations of cancer survivors.  Looking and being different: The survivors were hurt by gossip about their physical appearance.    3. Overcoming Difficulties  People: Many survivors got support from their family, friend, teacher, and other cancer survivors.  Coming to terms with cancer: . Making sense of cancer experiences helped survivors get through the difficult time of treatment and survivorship  Focusing on favorite things: Food was  one of the most important things that helped them cope, and some even said food saved and/or cured them.  4. The Future  Changes in perspectives:  appreciating relationships with loved ones and abandoning materialistic thoughts  Career: Many wanted to pursue careers helping others, such as social work, medicine, and research.  Picturing the future: Survivors were determined to prove their abilities despite societal stigma. | Small sample size, limited generalizability.    Participants were were relatively well adjusted to society, not representative of all survivors.    our study participants were relatively long-term  survivors    Urban only | Education programs to combat cancer stigma in schools and workplaces.    Support groups for young adult survivors to develop social skills.    Career counseling and workplace advocacy to ensure fair treatment.    Mental health services to address survivor self-esteem and relationship concerns. |
